# Toward a sustainable future: paradigm shifts, challenges, and convergence in green synthesis strategies for carbon materials

**DOI:** 10.3389/fchem.2026.1951078

**Published:** 2026-10-08

**Authors:** Xuexue Pan, Yi Zhao, Zhazira Supiyeva, Qamar Abbas

**Affiliations:** 1 Laboratory of Advanced Functional Materials, Zhongshan Advanced New Functional Materials Engineering Technology Research Center, Guangdong Engineering Technology Research Center, Zhongshan Polytechnic, Zhongshan, China; 2 School of Energy Science and Technology, Henan University, Kaifeng, China; 3 Laboratory of Engineering Profile, Satbayev University, Almaty, Kazakhstan; 4 Faculty of Chemistry and Chemical Technology, Al-Farabi Kazakh National University, Almaty, Kazakhstan; 5 Faculty of Chemical Technology, Poznan University of Technology, Poznan, Poland; 6 Institute for Chemistry and Technology of Materials, Graz University of Technology, Graz, Austria

**Keywords:** cross-disciplinary integration, green synthesis, paradigm shift, structure-performance relationship, sustainable carbon materials

## Abstract

As a cornerstone of modern industry, carbon materials face fundamental challenges in sustainability, precise structural control, and energy consumption through traditional synthesis methods. This review systematically analyzes four emerging green synthesis strategies driving a paradigm shift in this field: innovation at the source using biomass-derived carbon precursors to implement a circular economy; template-assisted synthesis (exemplified by MOF-derived carbon) enabling engineered customization of materials at multiple hierarchical levels; novel physical methods such as rapid Joule heating and microwave technology, which, by extreme compression of energy and time, open up new efficient and energy-saving synthesis pathways; and bottom-up organic synthesis, representing the pinnacle of atom-precision manufacturing and ultimate electronic structure control. Using ‘sustainability, precision, scalability’ as the analytical framework, this article delves into the principles, advantages, and core bottlenecks of each strategy. Beyond reviewing individual techniques, this paper emphasizes the positioning, comparison, and potential integration of different strategies, aiming to map a future blueprint from ‘extensive manufacturing’ to ‘green intelligent manufacturing.’ Finally, we point out that future research should focus on deepening the understanding of synthesis mechanisms, integrating data intelligence, and establishing full lifecycle assessment standards, ultimately achieving on-demand design and intelligent manufacturing of sustainable carbon materials.

## 1 Introduction

Carbon, the cornerstone element of both the living and non-living world, forms a variety of allotropes with vastly different properties—from diamond and graphite to graphene and carbon nanotubes—thanks to its unique ability to hybridize its electron orbitals (sp, sp^2^, sp^3^). It has created a rich and widely applicable family of carbon materials. In the wave of the contemporary technological revolution, carbon nanomaterials have become deeply embedded in the core areas of human society, becoming an invisible engine driving progress (Ayanda et al., 2024). In the energy sector, high-surface-area porous carbon is a key component of supercapacitor and battery electrodes, and its conductive network and pore structure directly determine energy storage density and power output (Sriram et al., 2023). Functionalized carbon materials are efficient, non-precious metal catalysts for water electrolysis, hydrogen production, and fuel cell oxygen reduction reactions in the hydrogen energy cycle (Fatima et al., 2025). In the environmental field, activated carbon with adjustable pore sizes and abundant surface functional groups is a key adsorbent for water treatment and air purification. At the same time, photocatalytic carbon-based composites offer a green solution for degrading organic pollutants (Liu Z. et al., 2022). In high-end electronics, graphene, with its extremely high carrier mobility, is a potential candidate for post-silicon semiconductor devices. At the same time, precisely structured carbon nanotubes and nanoribbons, due to their unique electrical and optical properties, show great potential in flexible electronics, sensors, and quantum information technologies (Zhang Z. et al., 2025). Carbon materials, with their broad performance versatility and universal applicability, have become a cornerstone of modern industry.

However, the traditional synthesis paradigms that support these remarkable applications increasingly face triple challenges in sustainability, precision, and cost-effectiveness, and their inherent limitations have become a bottleneck restricting the future development of carbon materials. First, in terms of precursor sources, mainstream methods (such as chemical vapor deposition and high-temperature pyrolysis of specific polymers) rely heavily on fossil-based raw materials like petroleum and coal, and obtaining them is itself associated with high carbon footprints and resource unsustainability (Mori, 2023; Natali et al., 2022). Secondly, in terms of the process, traditional high-temperature pyrolysis or chemical vapor deposition usually consumes a huge amount of energy and requires long reaction times (from several hours to several days), and often involves toxic or corrosive chemical reagents, which run counter to the global ‘dual carbon’ goals and the concept of green manufacturing. More importantly, in terms of structural control, many methods operate like a ‘black box’: it is extremely difficult to precisely and reproducibly control the microstructure of the final product (such as defect types and densities, edge structures, precise connectivity of pore networks, and atomic-level doping sites) by empirically adjusting macroscopic parameters like temperature and atmosphere. This results in understanding the ‘what’ of material performance without knowing the ‘why’, severely hindering the ‘materials by design’ approach for specific applications (van Blitterswijk, 2024). This traditional paradigm of ‘high input, high consumption, and low control’ is increasingly unable to meet the stringent demands of future technologies for extreme material performance, green manufacturing processes, and full life-cycle sustainability.

Therefore, a profound paradigm shift is brewing and emerging in the global carbon materials research community. The new synthesis paradigm aims to restructure the manufacturing logic of carbon materials fundamentally. Its core is built on three main pillars: first, sustainability, which emphasizes green, low-carbon, and circular processes from raw-material sourcing through production, prioritizing renewable or waste resources as precursors and developing low-energy, low-emission transformation technologies (Kumar and Choudhury, 2025). Second is precision, aiming for multi-level precise control over the atomic arrangement, nanostructure, and even the macroscopic morphology of carbon materials, achieving a leap from ‘fuzzy fabrication’ to ‘precise manufacturing,’ and guiding precise functions through precise structures (Ma Z. et al., 2025). Third is scalability, focusing on the economic feasibility and engineering potential of the synthesis route. While improving performance, it also considers cost control and the feasibility of large-scale production, ensuring that advanced materials can move from the laboratory to practical applications (Abdi et al., 2024). These three pillars are interconnected and mutually supportive, together defining the evolutionary direction of next-generation carbon material synthesis technology. Recent critical assessments of green synthesis reach the same conclusion: a route may be called green only when quantitative metrics—energy intensity, atom economy, E-factor or process mass intensity, and the renewability of the inputs—demonstrate an advantage over the incumbent process, and only when that advantage survives scale-up (Yadav et al., 2026).

To present this exciting paradigm shift systematically, this review uses the three pillars mentioned above as a unified analytical framework to conduct an in-depth analysis and comparison of the four most representative emerging green synthesis strategies. We first explore the ‘circular economy’ pathway based on biomass waste (Chapter 2), examining how it achieves green precursor substitution and the limitations imposed by the complexity of natural products. Next, we will focus on strategies for ‘engineered customization’ through template guidance (Chapter 3), particularly the prominent direction of metal-organic framework-derived carbon, analyzing its advantages and costs in precise structural replication and design. Then, we will review the ‘process revolution’ represented by rapid Joule heating and microwave technologies (Chapter 4), illustrating how innovations in physical methods minimize both energy consumption and time costs. Finally, we will touch on the pinnacle of chemical synthesis—the ‘bottom-up’ molecularly precise fabrication (Chapter 5)—and appreciate its ultimate control over the electronic structure of carbon materials, along with the profound conflict with scalable production. After clarifying the core, advantages, and challenges of each strategy, the main chapter of this article (Chapter 6) will aim to transcend the limitations of individual technologies by presenting a clear landscape of technology positioning through multidimensional comparisons and boldly envisioning innovative paths for complementary advantages through strategy integration. In the concluding chapter (Chapter 7), we will summarize the profound implications of this paradigm shift from ‘extensive manufacturing’ to ‘green intelligent manufacturing,’ and highlight key future research directions from the perspectives of fundamental mechanisms, digital tools, standardized evaluation, and interdisciplinary integration, to provide a systematic knowledge map and forward-looking inspiration for innovative development in sustainable carbon materials, both in China and worldwide.

For clarity and consistency, four interrelated but distinct metrics are used throughout this review: (i) sustainability—circularity and low cradle-to-gate environmental burden (waste-derived feedstock, low CO_2_ intensity) (Patrisia et al., 2027); (ii) scalability—feasibility of expansion from the laboratory to industry, governed by yield, throughput, and cost (Setiarto et al., 2026); (iii) precision—the degree of atomic/structural control over the final carbon (defect density, pore order, doping uniformity) (Kumar et al., 2026); and (iv) controllability—the ability to reproducibly program a target architecture through tunable process parameters (Shi et al., 2026). These definitions are applied uniformly in Sections 2–6.

The qualifier “low” attached above to the cradle-to-gate environmental burden of biomass-derived carbon—and the “Low (carbon-neutral feedstock)” entry in the Process Carbon Footprint row of Table 1—is a *conditional, system-boundary-dependent conclusion*, not an intrinsic or universal property of the route. It holds only when the full upstream chain is credited: (1) feedstock pretreatment—size reduction, milling, washing/acid-leaching for de-ashing, and sorting—which consumes mechanical/electrical energy and reagents and can be substantial for high-ash, lignocellulosic, or wet feedstocks (Bello et al., 2024); (2) drying/dewatering—the latent heat of water removal is often the single largest energy term for as-received (<10 wt% solids) biomass and must be included rather than treated as negligible (Derakhshandeh et al., 2026); (3) activation—high-temperature carbonization/activation (600 °C–1,000 °C) and, for chemical activation, the synthesis, corrosive-waste handling, neutralization, and recovery of the activator (commonly KOH, typically KOH: precursor ≈ 1–4:1), which can dominate both the CO_2_ footprint and operating cost (Gao et al., 2026); and (4) the energy source supplying every step above. Consequently, the apparent “near-neutral” or carbon-neutral character is meaningful only under a consistent cradle-to-gate LCA that assumes (a) renewable or low-carbon electricity/heat, (b) allocation/credit for the biogenic (waste-derived) feedstock, and (c) closed-loop recovery/recycling of the activating agent (Kim, 2017). Under a fossil-fuel grid or once-through activation scenario, the net CO_2_ and cumulative energy demand can rise materially, eroding the route’s advantage over fossil-derived precursors (Basha et al., 2026); a full cradle-to-gate LCA is therefore required before the value is interpreted as “near-neutral” (Martins et al., 2026). This qualification is now reflected in Table 1 footnote.

**TABLE 1:** Quantitative performance metrics of four carbon synthesis strategies across sustainability, scalability, and precision dimensions.

Metric category	Indicator	Unit	FJH (strategy A)	Bottom-up organic (strategy B)	Templating/MOF-derived (strategy C)	Biomass-derived (strategy D)
Sustainability	Energy Intensity	MJ/kg	∼1–5 (Zhuo and Levendis, 2014)	>50–100 (Crista et al., 2020a)	∼20–40 (Kim et al., 2022)	∼5–15
	Process Carbon Footprint	kg CO_2_-eq/kg	Low (if renewable grid) (Zhuo and Levendis, 2014)	High (solvent-intensive) (Crista et al., 2020a)	Moderate-High (Kim et al., 2022)	Low (carbon-neutral feedstock) (Zhang et al., 2026b)
	Material Yield	%	60%–90% (Zhuo and Levendis, 2014)	10%–30% (Crista et al., 2020a)	20%–50% (Kim et al., 2022)	30%–60% (Zhang et al., 2026b)
	E-Factor	kg waste/kg product	<1 (solvent-free) (Zhuo and Levendis, 2014)	>100 (Crista et al., 2020a)	10–50 (Kim et al., 2022)	2–10 (Zhang et al., 2026b)
	Renewability of Feedstock	%	Variable (often waste plastic) (Zhuo and Levendis, 2014)	Low (petrochemical) (Crista et al., 2020a)	Low (synthetic linkers) (Kim et al., 2022)	∼100% (renewable biomass) (Zhang et al., 2026b)
Scalability	Space-Time Yield	kg m^-3^ h^-1^	Very High (Zhuo and Levendis, 2014)	Very Low (Crista et al., 2020a)	Low-Moderate (Kim et al., 2022)	High (Zhang et al., 2026b)
	Process Complexity	# Unit Operations	1 (Single step) (Zhuo and Levendis, 2014)	>5 (Multi-step) (Crista et al., 2020a)	>4 (Synthesis + Removal) (Kim et al., 2022)	2–3 (Pre-treatment + Carbonization) (Zhang et al., 2026b)
	Reproducibility	% RSD	Low (<5%) (Zhuo and Levendis, 2014)	Moderate-High (Crista et al., 2020a)	Moderate (Kim et al., 2022)	High (>15%, due to heterogeneity) (Zhang et al., 2026b)
Precision	Pore Size Distribution Width	nm FWHM	Broad/Undefined (Zhuo and Levendis, 2014)	Narrow (<1 nm) (Crista et al., 2020a)	Very Narrow (<0.5 nm) (Kim et al., 2022)	Broad (>2 nm) (Zhang et al., 2026b)
	Surface Area Reproducibility	% RSD	Moderate (Zhuo and Levendis, 2014)	Low (<5%) (Crista et al., 2020a)	Low (<5%) (Kim et al., 2022)	High (>10%) (Zhang et al., 2026b)
	Graphitization Control	I_D_/I_G_I ​ Variance	High Variance (Zhuo and Levendis, 2014)	Low Variance (Amorphous) (Crista et al., 2020a)	Controlled (via MOF precursor) (Kim et al., 2022)	Uncontrolled (Amorphous) (Zhang et al., 2026b)
Sustainability	Representative product SSA	m^2^ g^-1^	Variable (graphene <100–1,000)	n/a (molecular)	1,000–3,000	500–3,500 (Divyashree and Hegde, 2015; Sevilla and Mokaya, 2014)
Scalability	Technology readiness (TRL)	-	7–8	2–3	3–4	8–9 (Zhang et al., 2026b; Zhuo and Levendis, 2014)
Precision	Typical SC electrode capacitance	F g^-1^	80–150	n/a	185–320 (Ahmad et al., 2020; Divyashree and Hegde, 2015; Mandal et al., 2026)	110–590
Sustainability	Cradle-to-gate CO_2_ (indicative)	kg CO_2_-eq kg^-1^	Low (renewable)	High	Moderate-High	Low (Kim et al., 2022; Zhang et al., 2026b)
Scalability	Key scale-up barrier	text	Conductive-network dependency; arcing	Multi-step; cost	MOF cost/yield; metal leaching	Feedstock heterogeneity; activation chemistry (Ding et al., 2022; Zhuo and Levendis, 2014)

Distinguishing this review from recent survey articles that predominantly catalogue individual synthesis routes in isolation (e.g., dedicated reviews of biomass-derived carbons, MOF-derived carbons, or flash graphene), the present work builds a unified, three-pillar analytical framework—sustainability, precision, and scalability—and uses it to (i) compare four emerging strategies on a common footing, (ii) position them quantitatively within a sustainability–precision–scalability map, and (iii) prospectively integrate them through cross-fusion pathways. Whereas prior reviews largely describe “what each method can do,” this article explicitly addresses the knowledge gap of “how the methods trade off and complement one another,” thereby providing a decision-support roadmap for route selection rather than a method-by-method inventory. To our knowledge, no single review has concurrently positioned biomass-derived, template/MOF-derived, physically accelerated (flash Joule heating and microwave), and bottom-up strategies within one comparative quadrant and linked each to quantitative life-cycle and scalability metrics (Algozeeb et al., 2020; Luong et al., 2020).

In summary, the distinctive contribution of this review is threefold: (i) it is, to our knowledge, the first work to benchmark four emerging green-synthesis families biomass-derived, template/MOF-derived, physically accelerated (flash Joule heating and microwave), and bottom-up under one quantitative sustainability-precision-scalability map; (ii) it replaces descriptive ‘method X can do Y′ narratives with cradle-to-gate life-cycle and scalability metrics (Sections 2–6; Table 1); and (iii) it proposes concrete cross-fusion routes for next-generation hybrid carbons (Section 6.3). This framing directly answers the reviewer’s question of what is new.

## 2 Source revolution: from ‘extraction’ to ‘circular’ biomass-derived carbon

In today’s era of pursuing sustainability, the field of carbon materials is undergoing a profound paradigm shift. At the core of this transformation is a fundamental shift in how we view raw materials. This is not just a redefinition of resources, but a materials science revolution from linear extraction to a circular economy.

Traditional carbon material synthesis (such as graphene, carbon nanotubes, etc.) mainly relies on extracting fossil fuels like petroleum and natural gas. This ‘top-down’ extraction model is inherently unsustainable (Bafqi et al., 2024; Iurchenkova, 2025). In contrast, biomass-derived carbon strategies focus on the most abundant yet easily overlooked waste in human production and daily life: agricultural residues (rice husks, straw, fruit shells), forestry by-products (sawdust, bark), food processing waste, and even urban sludge (Thakur et al., 2024). These once ‘environmental burdens’ are now being redefined as renewable precursor reservoirs rich in elements such as organic carbon, oxygen, and nitrogen.

The transformation from waste to functional carbon mainly relies on two types of thermochemical technologies, which follow distinct physicochemical pathways and yield carbon products with different properties (Figures 1a,b; Durak, 2023; Jimenez Forero, 2024). Specifically, hydrothermal carbonization is a wet-thermal conversion process carried out in a subcritical water medium, typically occurring at relatively low temperatures (180 °C–250 °C) and autogenous pressure. This process simulates natural coalification and converts high-moisture biomass waste into hydrochar rich in oxygen-containing functional groups through dehydration, decarboxylation, and condensation reactions. The surface characteristics of hydrochar tend to be more hydrophilic and chemically active (Figure 1a; Durak, 2023). In contrast, pyrolysis is a high-temperature process carried out in an anaerobic or oxygen-deficient environment (usually 300 °C–900 °C). It involves the cleavage, recombination, and aromatization of biomass macromolecules, ultimately producing pyrolytic carbon with a highly aromatic carbon framework. As pyrolysis progresses, volatile components are gradually released, the carbon ordering of the solid products continuously increases, and the pore structure develops, making them stand out for their electrical conductivity and physical adsorption (Figure 1b; Durak, 2023). The products of these two pathways—hydrothermal carbon and pyrolytic carbon—have complementary application potential in environmental remediation, energy storage, and catalysis because of their structural differences.

**FIGURE 1:**
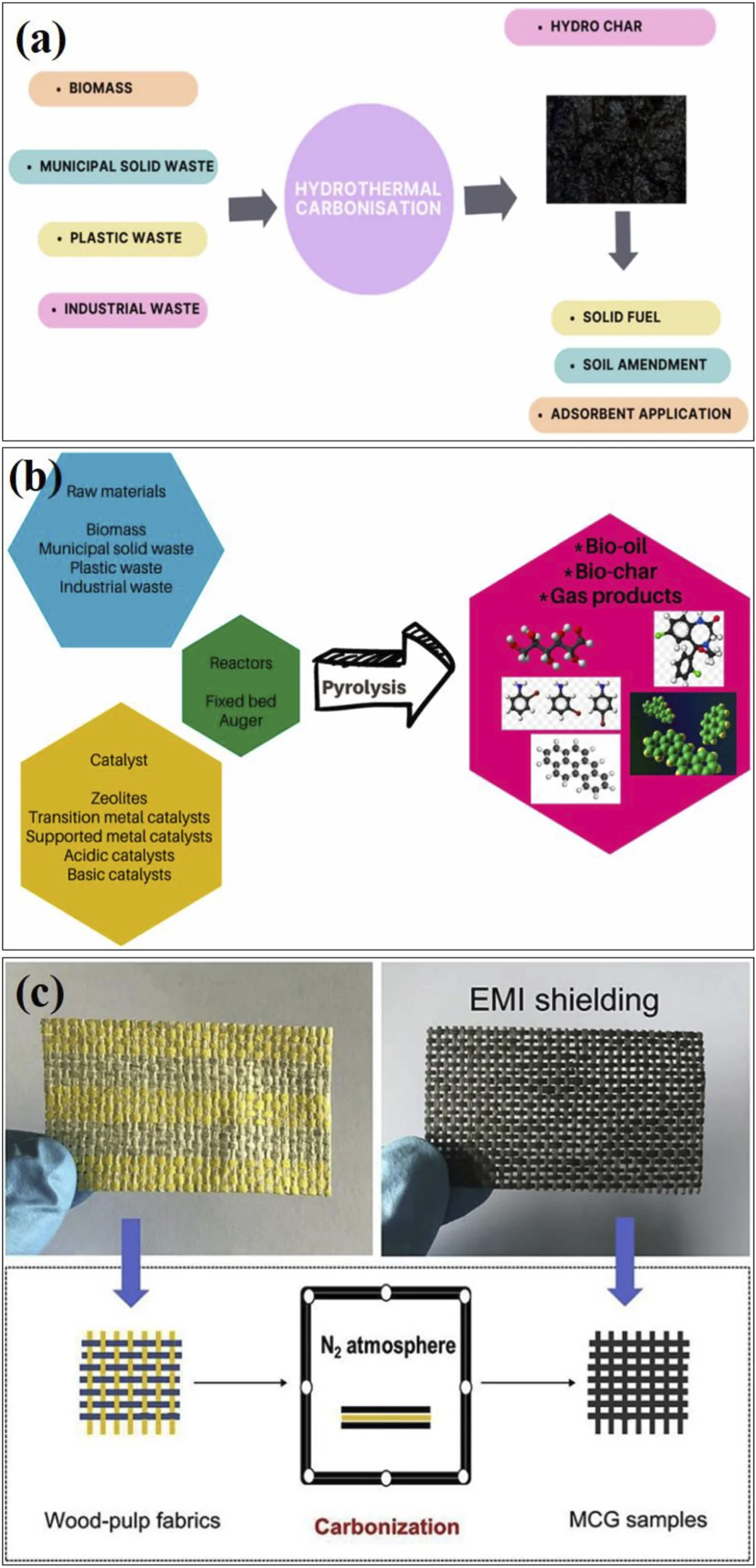
**(a)** Schematic representation of the HTC process (Durak, 2023). **(b)** Schematic representation of the pyrolysis process stages (Durak, 2023). **(c)** Fabrication process of semi-transparent biomass-derived macroscopic carbon grids (MCG) for EMI shielding. The process begins with wood-pulp fabrics, which are then carbonized in an N_2_ atmosphere to produce the final MCG samples. The images show the wood-pulp fabric before carbonization and the resulting carbon grid used for EMI shielding (Afridi et al., 2024).

First is hydrothermal carbonization (HTC), a technology carried out under high-temperature and high-pressure aqueous conditions (typically 180 °C–250 °C), simulating the natural ‘carbonization’ process of wood decaying into coal (Durak, 2023). Its core advantage is efficiently utilizing water-containing waste (such as sludge and algae) while avoiding the high-energy drying process; the resulting products typically include hydrothermal carbon, carbon dots, and carbon-based composites (Fahad Halim et al., 2025). Its chemical mechanism mainly involves the hydrolysis, isomerization, and dehydration of cellulose under hydrothermal conditions to form intermediates such as 5-hydroxymethylfurfural, which then polymerize into core-shell structured colloidal carbon spheres. Additionally, protein components can introduce nitrogen doping, giving the product specific functional groups and electrochemical activity (Modugno, 2023; Peng et al., 2023). Another type is pyrolysis/activation, a high-temperature thermal treatment carried out in an inert atmosphere (usually 400 °C–1,000 °C). Its characteristic is preserving the original material’s form; the fibrous structure of biomass (such as the bundle structure of bamboo or the lamellar structure of rice husks) is often retained after high-temperature carbonization, resulting in biochar with abundant pores (Figure 1c; Afridi et al., 2024). For example, wood pulp fabric can be carbonized in a nitrogen atmosphere to produce a translucent, biomass-derived macroscopic carbon grid (MCG) for electromagnetic interference shielding. Figure 1c shows the wood pulp fabric before carbonization and the morphology of the carbon grid obtained after carbonization (Afridi et al., 2024). The products include not only biochar but also bio-oil and syngas. Subsequent chemical activation (such as KOH activation) can further increase its specific surface area, making it an ideal precursor for energy storage materials like supercapacitor electrodes (Mehdi et al., 2022; Panwar and Pawar, 2022).

These carbon materials derived from these sources not only address waste disposal issues but also demonstrate unique value in macroscopic materials. For example, carbon dots or hydrothermal carbon produced through hydrothermal carbonization possess semiconductor properties and can generate photo-induced electron-hole pairs. They can act as independent photocatalysts or be combined with traditional photocatalysts like TiO_2_, significantly enhancing the light absorption range and electron separation efficiency, thereby efficiently degrading water pollutants or achieving CO2 reduction (Figures 2a–c; Chen Z. et al., 2024; López et al., 2025; Zourou et al., 2025). As shown in Figure 2, the mechanism mainly includes the following aspects: Figure 2a illustrates the possible charge separation and migration pathways when hydrothermal carbon (HTC) is used as an independent photocatalyst (Chen Z. et al., 2024). Figure 2b reveals the mechanism by which oxygen-containing functional group (OFG)-modified carbon dots (CDs) function in the photocatalytic process (Chen Z. et al., 2024). Figure 2c summarizes the various ways carbon materials promote charge carrier generation, separation, and transfer in composite photocatalysts (HC-composites), including photosensitization, upconversion luminescence, acting as electron/hole acceptors, forming Z-type or type-II heterojunctions, and serving as electron bridges (Chen Z. et al., 2024). The porous structure and high degree of graphitization in the pyrolysis products make them high-performance electrode materials (Nguyen et al., 2025; Wang J. et al., 2025). Through modification methods such as ‘template-assisted’ or ‘microwave-assisted’ techniques, researchers can regulate pore size distribution and surface functional groups, enabling ‘customized’ design for specific applications like lithium-ion batteries and supercapacitors (Aggarwal et al., 2025).

**FIGURE 2:**
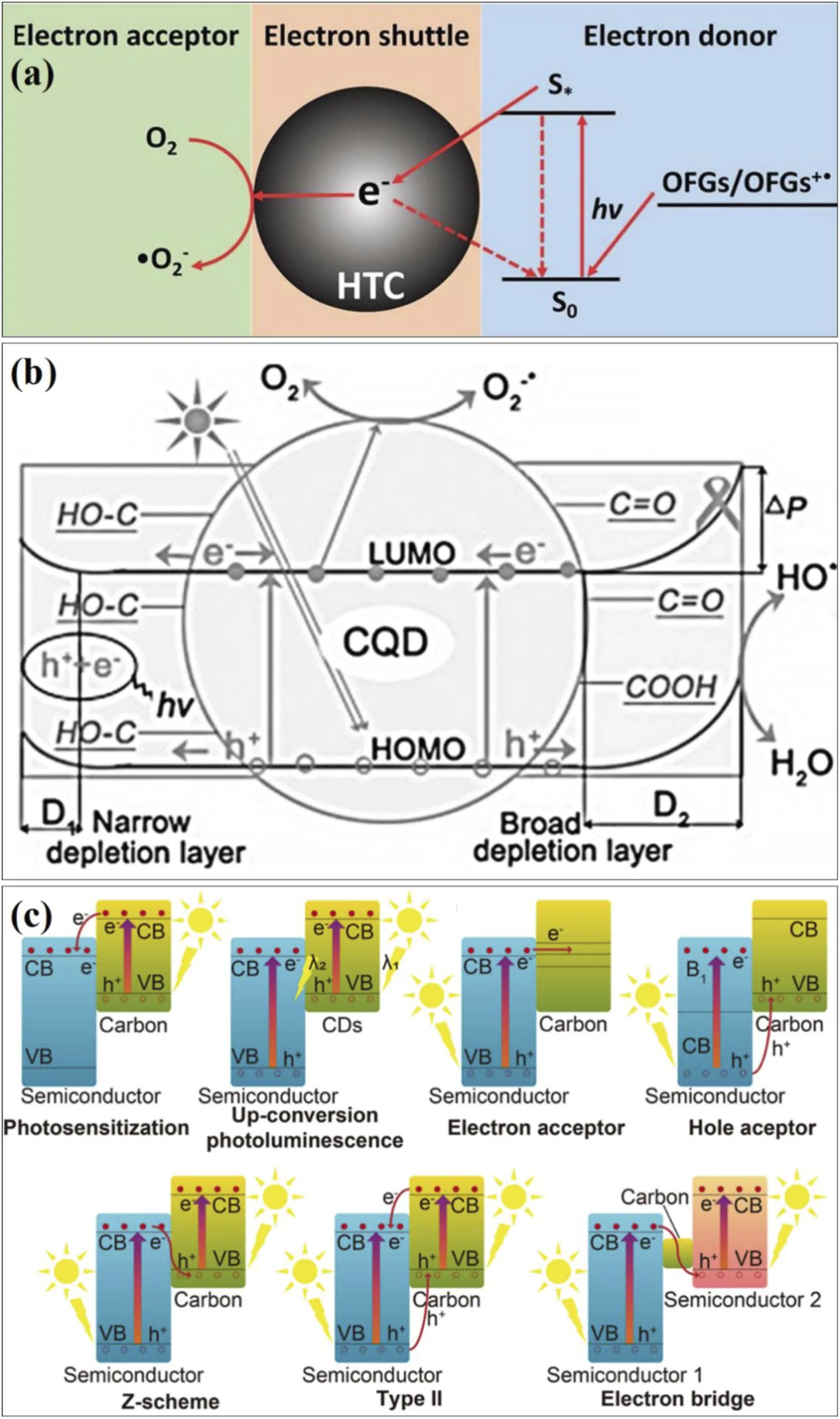
**(a)** Photocatalysis of hydrochars: possible photocatalytic mechanism of hydrochar (HTC) (Chen Z. et al., 2024). **(b)** Photocatalytic mechanism of CDs with surface oxygen-containing functional groups (OFGs) (Chen Z. et al., 2024). **(c)** Effects of carbons on generation, separation, and transfer of charge carriers in HC-composite photocatalysts: photosensitization; up-conversion photoluminescence; electron acceptor; hole acceptor; Z-scheme system; type II system; electron bridge (Chen Z. et al., 2024).

Therefore, the core philosophy of biomass-derived carbon strategies lies in ‘extracting value from waste’ (Belluati et al., 2024; Khandaker et al., 2025). Through green, gentle conversion via hydrothermal carbonization or efficient structure construction through pyrolysis/activation, we not only address resource waste but also provide a practical path for the green and sustainable development of carbon materials (Cai et al., 2024; Nisa et al., 2025).

### 2.1 Analysis of core conversion technologies

#### 2.1.1 Hydrothermal carbonization: process, mechanisms, and functionalization of ‘hydrochar’

Hydrothermal Carbonization (HTC) is a green chemical technology that mimics the natural coal formation process (Singh et al., 2024; Xu et al., 2022). It converts biomass into carbon-rich solids, commonly referred to as “hydrochar” (Figure 3a), in a sealed reactor using water as the only reaction medium and partial reactant, under relatively mild conditions (typically at temperatures of 180 °C–250 °C and autogenous pressure) (Petrović et al., 2024). Its chemical reactions are inherently very complex. In subcritical or supercritical water environments, the main organic components of biomass—cellulose, hemicellulose, and lignin—undergo a series of intense hydrolysis, dehydration, decarboxylation, condensation, and even aromatization reactions (Martins-Vieira et al., 2023). These reactions break the original macromolecular structure of the biomass, remove many oxygen heteroatoms, and rearrange the carbon framework, transforming the originally soft organic matter into a hard carbonaceous material (Figure 3a; Carrasco Díaz et al., 2024). Where the target product is a liquid fuel or platform molecule rather than a solid hydrochar, the route is instead complemented by catalytic hydroprocessing of bio-derived oils, for which Ni-based bifunctional catalysts with variable acidity are used to remove oxygen through hydrodeoxygenation (Figure 3b; Carrasco Díaz et al., 2024).

**FIGURE 3:**
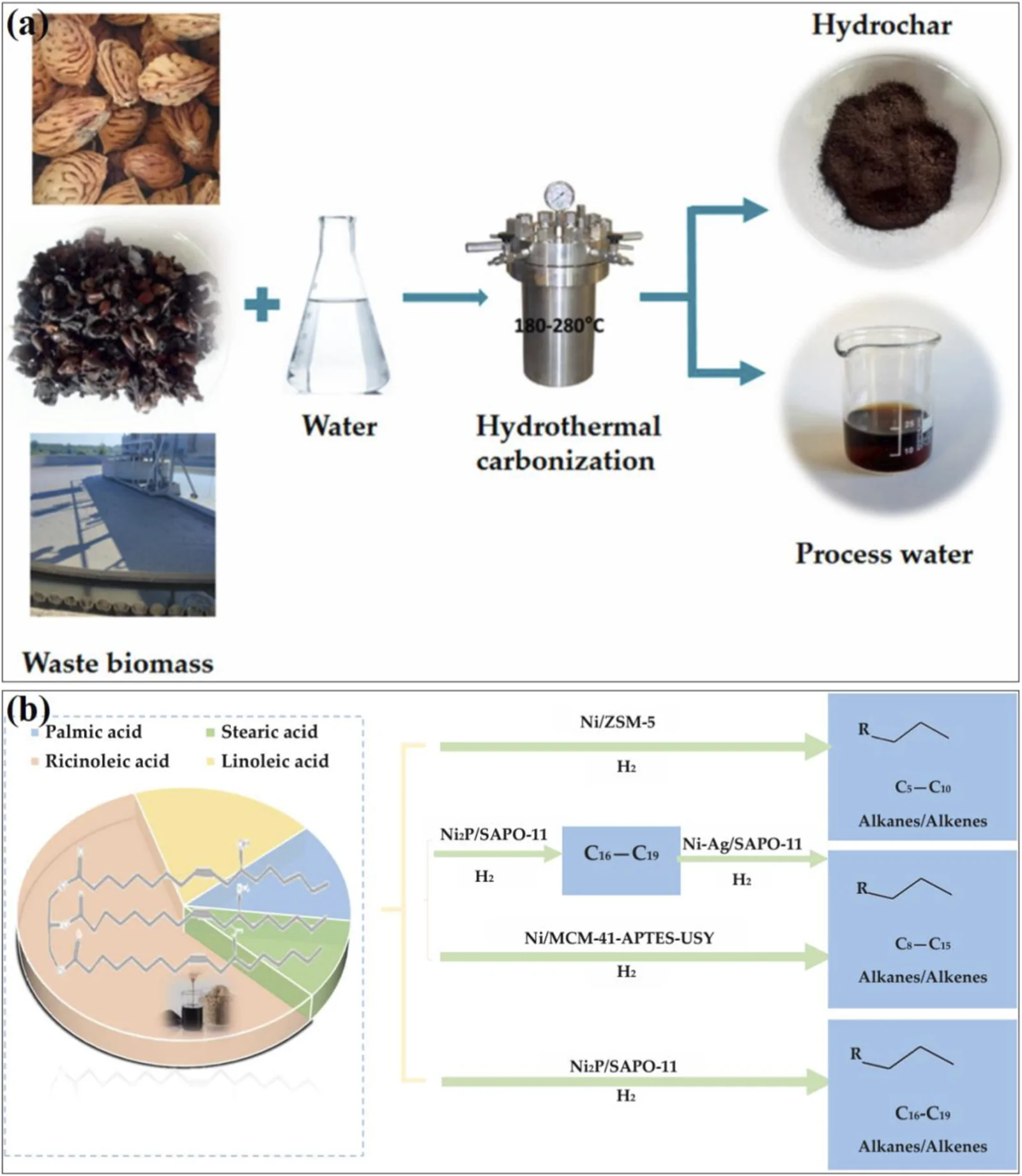
**(a)** Schematic representation of the HTC process (Carrasco Díaz et al., 2024). **(b)** Hydroprocessing castor oil by Ni-based bifunctional catalysts with variable acidity (Carrasco Díaz et al., 2024).

The charm of this process lies in its ‘green’ attributes. First, HTC usually does not require additional precious-metal catalysts, and water itself is an inexpensive, non-toxic, and harmless green solvent (Ng et al., 2025). Second, it can efficiently process high-moisture biomass materials, addressing the high energy consumption of traditional drying methods (Ojewumi and Chen, 2024). After the reaction is complete, the resulting hydrothermal carbon often forms spherical or irregularly shaped particles with surfaces rich in oxygen-containing functional groups such as hydroxyl (-OH), carboxyl (-COOH), and carbonyl (C=O), which give it inherent hydrophilicity (Figure 4a; Chen Z. et al., 2024). This hydrophilicity makes hydrothermal carbon not only an excellent carrier for metal nanoparticles but also a highly effective adsorbent for pollutants, with widespread applications (Ghamarpoor et al., 2025; Hsu et al., 2023). However, hydrothermal carbon in its original state usually appears as amorphous carbon, with less-than-ideal electrical conductivity and pore structure. Therefore, subsequent treatment is usually required to achieve specific functionalization. Heat treatment (such as annealing) can significantly improve its degree of graphitization and electrical conductivity, making it suitable for electrode materials (Roy et al., 2024). Introducing precursors containing elements such as nitrogen, sulfur, or phosphorus for *in situ* doping, or using chemical reagents for post-treatment, can introduce active sites on their surfaces, specifically for catalysis or targeted adsorption applications (Figures 4b,c; Veselov and Vedyagin, 2023).

**FIGURE 4:**
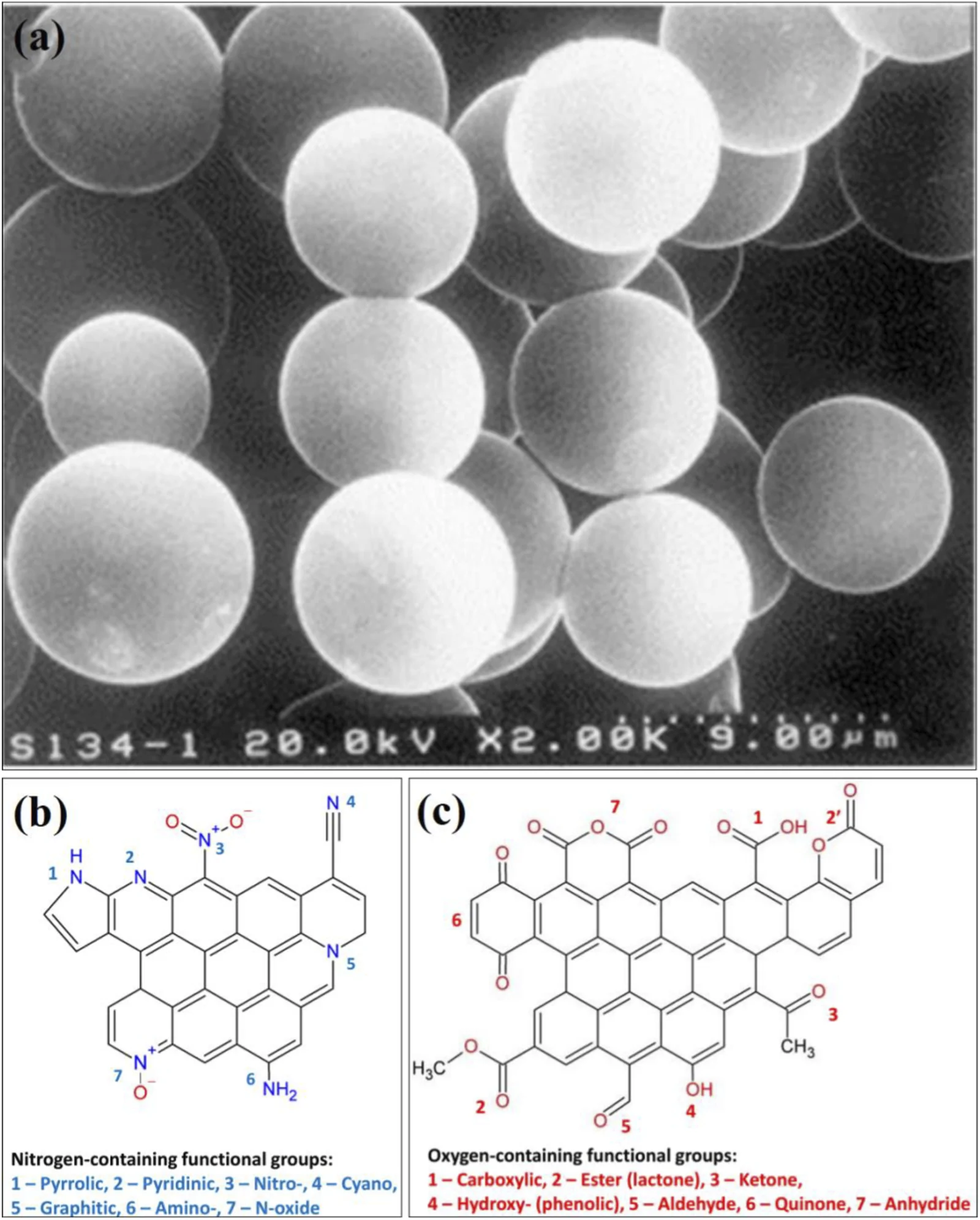
**(a)** SEM image of glucose-derived hydrochar (Chen Z. et al., 2024). Types of nitrogen-containing **(b)** and oxygen-containing **(c)** functional groups found on the surfaces of CXs (Veselov and Vedyagin, 2023).

Although HTC shows potential for controlling product morphology and surface chemistry, the microstructure and porosity of the final product are still largely constrained by the inherent composition of the raw materials (Fahad Halim et al., 2025; Nobrega et al., 2024). Therefore, to achieve precise control over spatial structures, further research and exploration are still needed in precursor selection and subsequent process optimization.

#### 2.1.2 Pyrolysis and activation: precise control of pores and surface chemistry through process parameters

Unlike HTC’s ‘wet’ approach, pyrolysis is a ‘dry’ process in which biomass is decomposed into biochar, bio-oil, and syngas at high temperatures (typically >400 °C and can exceed 1,000 °C) in an inert (oxygen-free) atmosphere (Adeoye et al., 2023; Luo and Zhou, 2025). The core advantage of pyrolysis lies in its process simplicity and energy efficiency (Pan et al., 2024). Biomass is pyrolyzed in a high-temperature inert atmosphere, removing most of the volatiles and oxygen-containing functional groups, leaving behind a carbon-rich ‘skeleton’—biochar (Hayder and Naim, 2025). However, for the preparation of porous carbon materials, the ‘biochar’ obtained from the direct pyrolysis of biomass, although rich in carbon, often has a specific surface area and porosity that cannot meet the requirements of high-performance applications (such as supercapacitors and efficient adsorption) (Figures 5a–c; Wang Q. et al., 2024; Yin et al., 2023). Figure 5a illustrates the system for preparing biomass-derived carbons (BDCs) from different biomass feedstocks through hydrothermal carbonization. To overcome this performance bottleneck, activation treatment is usually required to regulate pore structure (Wang Q. et al., 2024). As shown in Figure 5b, treating the precursor with different types of activators can significantly alter the pore development of the carbon material (Wang Q. et al., 2024). Figure 5c further compares the evolution of the pore structure of carbon materials under different activation conditions (such as different burn-off rates) through nitrogen adsorption/desorption isotherms at 77 K: as the carbon dioxide activation time is extended (from 15 min to 240 min), the burn-off rate of the material gradually increases from 1% to 88%, and its micropore and total pore volume also change significantly, intuitively reflecting the key influence of the degree of activation on pore development (Wang Q. et al., 2024).

**FIGURE 5:**
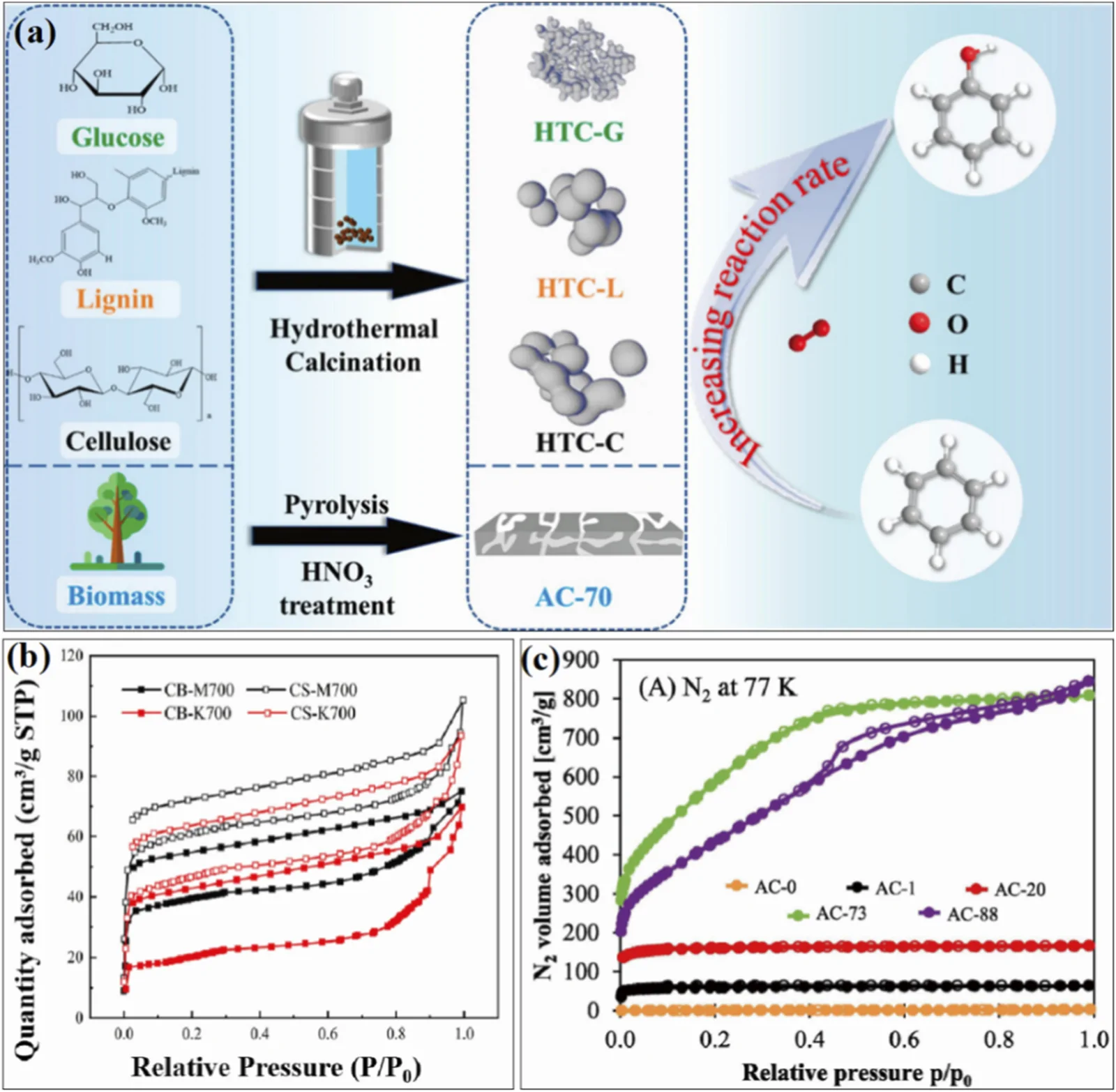
**(a)** Preparation of BDCs from different types of biomass through hydrothermal carbonization (Wang Q. et al., 2024). **(b)** Schematic illustration of the preparation of porous carbonaceous materials treated by different activators (Wang Q. et al., 2024). **(c)** Adsorption/desorption isotherms of nitrogen at 77 K (the AC-0 sample was heated under an inert atmosphere at 1273 K). All the other samples were physically activated with carbon dioxide at 1273 K. Burn-offs of 1%, 20%, 73%, and 88% were confirmed for materials activated for 15, 60, 120, and 240 min, respectively. Samples were named according to measured burn-off, i.e., AC-1, AC-20, AC-73, and AC-88, respectively (Wang Q. et al., 2024).

This is mainly because the carbon skeleton obtained directly through pyrolysis is relatively dense, lacks sufficient micropores and mesoporous channels, and cannot provide adequate ion-transport channels or adsorption sites. Therefore, “activation” is a key refinement and engineering step aimed at creating a rich, accessible pore network and high specific surface area. Activation techniques essentially involve “secondary processing” of the carbon skeleton, generating pores by selectively etching the carbon framework (Liu et al., 2026; Wen et al., 2025).

Activation is mainly divided into two categories: physical activation and chemical activation.

##### 2.1.2.1 Physical activation

Biochar is usually selectively oxidized and etched at high temperatures using CO_2_ or steam. This process uses the reaction between the gas and the carbon surface to, etch pores. Although it is relatively mild and environmentally friendly, it often requires higher temperatures and longer reaction times to achieve the desired porosity (Li et al., 2024).

##### 2.1.2.2 Chemical activation

Involves mixing the biomass with an activating agent (such as KOH, ZnCl_2_, H_3_PO_4_, etc.) before pyrolysis (Verma and Nguyen, 2024). This method is regarded as the ‘gold standard’ for creating pores, especially KOH activation, which is highly favored for its excellent pore-forming ability (capable of producing an ultrahigh specific surface area of over 3,000 m^2^/g).

The mechanism of KOH activation is particularly complex and efficient, involving multiple processes such as redox reactions between KOH and carbon, and the intercalation and escape of metallic potassium. (Cui et al., 2025). First, KOH reacts with carbon at high temperatures, producing gaseous products (such as CO_2_ and H_2_). These gases create pressure inside the carbon, tearing the carbon framework and forming pores (Figures 6a–c; Nandi et al., 2023). Figure 6a shows the relatively smooth and dense surface morphology of the raw rice husk-based biochar (RHBC) (Nandi et al., 2023). In contrast, the surfaces of the porous carbons PAC1 (Figure 6b) and PAC2 (Figure 6c), prepared after KOH activation, exhibit obvious etching and pore structures (Nandi et al., 2023). As the KOH-to-biomass ratio changes, the pore morphology and pore density of the resulting carbon material also change significantly, directly confirming the key role of KOH etching in pore development. Additionally, the generated metallic potassium (K) strongly intercalates into the carbon’s graphite layers, pushing apart the interlayer spacing and escaping at high temperatures, thus forming many micropores and mesopores (Kim HS. et al., 2024). This intercalation-expansion-escape process is the key to why KOH activation can produce an extremely high specific surface area.

**FIGURE 6:**
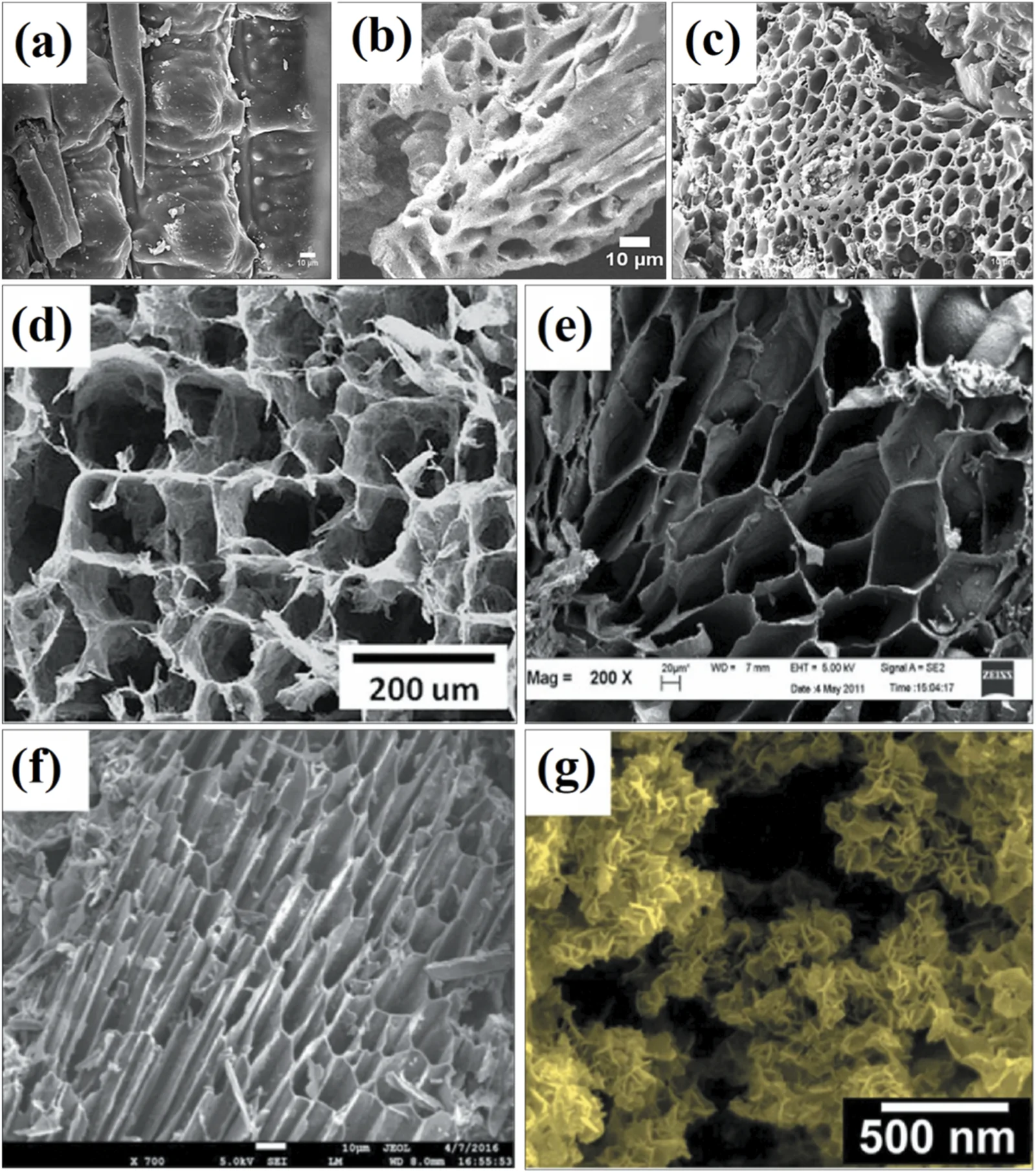
SEM images of **(a)** RHBC and **(b, c)** activated carbons PAC1 and PAC2, respectively, prepared with different KOH-to-biomass ratios at a temperature of 600°C (Nandi et al., 2023). SEM images of carbon-based materials derived from biomass wastes: **(d)** lignin-modified graphene aerogel from corncob; **(e)** activated carbon from rambutan peel; **(f)** biochar from wheat straw; and **(g)** graphitic-carbon nanoflakes from green tea waste.

By precisely controlling the core process parameter matrix of ‘temperature-time-atmosphere-activator/precursor ratio,’ it is possible to finely regulate the pore structure, degree of graphitization, and surface chemistry of the final carbon material. For example, a lower activation temperature helps preserve the original oxygen-containing functional groups of the biomass, which are very beneficial for capacitive adsorption or acidic catalytic reactions (Liu Z. et al., 2025). A higher activation temperature promotes the growth of graphite microcrystals and the formation of a conductive network, making the material more suitable for electrochemical energy storage (such as lithium-ion battery electrodes) (Zeng et al., 2022; Zhang D. et al., 2022). This ability to control structure through processing parameters and customize performance makes the pyrolysis-activation route the most mainstream and engineering-potential technique for preparing biomass-derived functional carbon materials (Figures 6d–g; Zhou and Wang, 2020). This strategy of precisely controlling process parameters to tailor material structures and thereby optimize specific properties gives the pyrolysis-activation route great flexibility and universality (Zhou and Wang, 2020). For this reason, this technology has become the most mainstream and engineering-promising technical pathway in the current field of biomass-derived functional carbon material preparation. Figures 6d–g show the diverse carbon material morphologies obtained through this route: from lignin-modified graphene aerogels derived from corn cobs (Figure 6d) to activated carbon prepared from mangosteen peels (Figure 6e; Zhou and Wang, 2020). Biochar obtained from the pyrolysis of wheat straw (Figure 6f) to graphite-like carbon nanosheets derived from green tea waste (Figure 6g; Zhou and Wang, 2020). These functional carbon materials, with diverse forms and adjustable structures, demonstrate the broad prospects of the pyrolysis-activation route for high-value utilization of biomass resources. It not only addresses the problem of low-value use of biomass but also, through pore engineering and surface functionalization, provides ample opportunities for the development of green energy, environmental management, and high-performance materials (Yin et al., 2023; Zhou and Wang, 2020).

To move from qualitative description to quantitative benchmarks, representative biomass-derived carbons reported in the literature deliver specific surface areas of 500–3,500 m^2^ g^-1^ and, as supercapacitor electrodes in aqueous electrolytes, specific capacitances of approximately 110–590 F g^-1^ (e.g., corncob-derived activated carbon: 3,530 m^2^ g^-1^, ∼401 F g^-1^ in 1 M H_2_SO_4_; coconut-shell-derived activated carbon: 2000 m^2^ g^-1^, 250 F g^-1^; rice-husk-derived activated carbon: 1768 m^2^ g^-1^, 171 F g^-1^; sugarcane-bagasse-derived activated carbon: 2,871 m^2^ g^-1^, 109 F g^-1^ (Divyashree and Hegde, 2015); device-level energy densities of 12–24.5 Wh kg^-1^ for symmetric cells (Mandal et al., 2026)). Sevilla and Mokaya showed that KOH activation of hydrothermally pre-carbonized biomass routinely affords superactivated carbons with SSA >3,000 m^2^ g^-1^ and capacitance approximately 150–250 F g^-1^ (Sevilla and Mokaya, 2014). Table 2 compiles these values together with the corresponding design conditions (activation agent, temperature, electrolyte).

**TABLE 2:** Representative biomass precursors and the performance of their derived carbons in supercapacitor applications (compiled from recent reviews (Mandal et al., 2026)). SSA = BET specific surface area; values illustrate how precursor identity and activation chemistry govern pore characteristics and electrochemical performance. (Introduced here for numbering consistency; it quantitatively supports the biomass discussion in Section 2).

Biomass precursor	Activation/chemistry	SSA (m2 g-1)	Specific capacitance (F g-1) and conditions	Electrolyte	Key design condition	Ref.
Corncob	KOH activation	3,530	401.6 (@1 A g-1)	1 M H2SO4	High KOH:precursor ratio; 2-step carbonization	Divyashree and Hegde (2015)
Coconut shell	KOH activation	2000	250 (@1 A g-1)	1 M H2SO4	-	Divyashree and Hegde (2015)
Rice husk	ZnCl_2_ activation	1768	171 (@1 A g-1)	0.5 M NaOH	-	Divyashree and Hegde (2015)
Sugarcane bagasse	ZnCl_2_ activation	2,871	109 (@1 A g-1)	1 M H2SO4	-	Divyashree and Hegde (2015)
Sunflower seed	KOH activation	2,585	244 (@1 A g-1)	0.1 M HCl	-	Divyashree and Hegde (2015)
Coconut shell (device)	KOH activation	-	340 (@1 A g-1); 16 Wh kg-1	2 M KOH	-	(Mandal et al., 2026)
Rice husk (device)	-	-	168 (@1 A g-1); 24.5 Wh kg-1	Na2SO4 (aq.)	-	(Mandal et al., 2026)
Banana peel (device)	KOH activation	-	312 (@1 A g-1); 14.5 Wh kg-1	6 M KOH	-	(Mandal et al., 2026)
Wheat straw (device)	KOH (gel polymer)	-	270 (@1 A g-1); 12.5 Wh kg-1	PVA/H2SO4	-	(Mandal et al., 2026)
Cotton stalk (device)	KOH activation	-	295 (@1 A g-1); 14.2 Wh kg-1	1 M KOH	-	(Mandal et al., 2026)

### 2.2 Core challenge: the fundamental contradiction between heterogeneity and controllability and strategies to address it

Although biomass-derived carbon strategies offer unparalleled sustainability advantages, they face an inherent scientific challenge: a profound contradiction exists between the intrinsic, complex chemical composition and structural heterogeneity of natural precursors and the precise control materials science requires for uniform product structure and reproducible performance (Wang Z. et al., 2023). The same type of biomass (such as rice husks) can differ significantly in components like cellulose, lignin, and ash (mainly SiO_2_) depending on its origin, variety, growing conditions, and harvesting season (Hamidu et al., 2025). This ‘source uncertainty’ directly leads to significant variations in pore structure, conductivity, surface chemistry, and even mechanical strength between different product batches, even when the same pyrolysis/activation process is used, severely hindering standardization and reliable large-scale application (Razzak, 2024).

To address this challenge, it is necessary to shift from ‘passive adaptation’ to ‘proactive intervention’ and develop multi-level strategies.

#### 2.2.1 Pretreatment and homogenization of precursors

Develop methods such as mechanical crushing, classification, chemical pretreatment (such as acid leaching for de-ashing and component separation), and even biological treatment (enzymatic hydrolysis) to minimize the physical and chemical heterogeneity of the raw materials as much as possible (Chen J. et al., 2024; Joshi and Manjare, 2024). For example, selectively removing lignin or hemicellulose through mild chemical treatment can yield homogenized raw materials with higher cellulose content, producing carbon materials with more predictable structures (Jiang et al., 2023).

#### 2.2.2 Mechanism study and predictive modeling of structure-performance relationships

In-depth investigation of the evolution pathways of specific biomass components (such as cellulose contributing to easily graphitizable carbon, lignin contributing to cross-linked hard carbon) during thermochemical conversion, and establishing a structure-activity relationship map from ‘precursor composition/structure → pyrolysis intermediates → final carbon structure’ (Hall et al., 2025; Zhang et al., 2025b). By combining machine learning with large amounts of experimental data to train models, it is expected to enable preliminary predictions of product performance from specific biomass feedstocks under given processes (Yadav et al., 2025).

#### 2.2.3 Precise control and standardization of the process

Develop more advanced online monitoring and feedback control technologies to accurately and in real time regulate the temperature field and atmospheric composition during the pyrolysis/activation process, partially offsetting the impact of raw material fluctuations and improving batch stability (Zhong et al., 2025).

#### 2.2.4 Application-oriented ‘tiered utilization’ and ‘defect engineering’ thinking

Shift your mindset, not striving to produce a ‘universal’ super carbon material from all types of biomass, but instead applying ‘tiered utilization’ based on their compositional characteristics. For example, high-ash biomass can be used to prepare silicon-rich carbon composites for lithium battery anodes (Ali et al., 2021a). Seaweed with high nitrogen content can be preferentially used to prepare nitrogen self-doped carbon for oxygen reduction catalysis (Zhang J. et al., 2025). At the same time, consider the inherent impurity elements in biomass (such as N, P, S, K) as ‘natural dopants,’ and transform them into functional active sites through process design, turning ‘impurities’ into ‘features’ and achieving functionalized guidance of defects (Yruela-Garrido et al., 2025).

Therefore, the path of biomass-derived carbon is a promising yet challenging route to sustainability. Its future depends not only on the continuous optimization of processing technologies but also on whether we can profoundly understand and master, at the fundamental scientific level, the ‘black box’ transformation from complex biomass precursors to functional carbon materials. This would turn ‘turning waste into treasure’ from a hopeful vision into a precise science that is predictable, reproducible, and designable.

From a critical, techno-economic perspective, four quantitative constraints must be weighed against the green credentials of biomass routes (i) Precursor heterogeneity: ash content (e.g., SiO_2_ in rice husk). and cellulose/lignin ratios vary widely with feedstock and season, directly producing batch-to-batch fluctuations in surface area and conductivity (Hamidu et al., 2025; Razzak, 2024). (ii) Carbon yield: hydrochar yields typically range from 55 to 94 wt% depending on feedstock and HTC severity (180 °C–280 °C, autogenous pressure), whereas high-temperature pyrolysis of the hydrochar affords only 20–35 wt% final char (Maniscalco et al., 2020; Wang et al., 2023b); most of the carbon and the embedded energy therefore leave as gas/oil. (iii) Energy consumption: HTC dewatering of wet biomass consumes ≈846 kJ kg^-1^ and saves >60% of the thermal/electrical energy of conventional hot-air drying. Still, the downstream pyrolysis/activation step (600 °C–1,000 °C) is energy-intensive, and chemical activation with KOH typically uses a KOH-to-precursor mass ratio of 1–4:1, with the spent alkali requiring neutralization and recovery (Wang et al., 2023b). (iv) Scalability: industrial-scale HTC plants already exist, and the route is among the most readily scalable of the four strategies (Maniscalco et al., 2020), yet feedstock collection, drying, and consistent activation chemistry remain logistical barriers to product uniformity at ton scale. These trade-offs mean biomass-derived carbon is optimal where low-cost, abundant feedstock and circular-economy value outweigh the need for atomic-level precision.

## 3 Engineered construction: precise customization of porous structures using the template methods

If biomass-derived carbon strategies represent a sustainable revolution at the source of raw materials, then the template approach is a victory of ‘reverse thinking’ in the precise construction of material structures. It is not only an improvement in processing routes but also a whole new engineering philosophy: shifting from ‘passive acceptance’ to ‘active design’ (Zhang, 2025). Traditional pyrolysis processes often rely on the ‘growth’ or ‘pyrolysis’ of homogeneous precursors, and their pore structures are constrained by the randomness of thermodynamics and kinetics, making it difficult to escape the fate of ‘every material has its destined use (Rasaq et al., 2024; Talwar et al., 2025). The template method completely overturns this conventional way of thinking. It abandons the passive approach of blind heating and waiting, and instead adopts a proactive, engineering-oriented design philosophy. At the core of this strategy is ‘creating a template first.’ Researchers preemptively create a ‘mold’ (template) with an ideal nanostructure. This process can be extremely precise—from nanoscale control of pore sizes to macroscopic arrangement in three-dimensional space, and even the exact design of pore geometries (such as cylindrical, spherical, or layered). Subsequently, a carbon source is filled into or wrapped around this mold. After the material is carbonized and shaped at high temperatures, the template is cleverly removed. The power of this ‘morphology replication’ strategy lies in its ability to break the thermodynamic randomness limitations of traditional carbonization processes, enabling unprecedentedly precise design of carbon material structures. We no longer passively ‘discover’ material structures; we can ‘engineer’ to ‘replicate’ or ‘customize’ structures. This proactive, reverse-thinking approach allows precise control of pore size, geometry, spatial arrangement, and even dimensionality at mesoscopic and macroscopic scales. Whether applications require ultra-large pores for rapid fluid transport, ultra-small pores for efficient molecular adsorption, or highly ordered three-dimensional networks for structural stability, the template method can meet these needs through engineering means. Therefore, the template method is not just a material fabrication technique but a key pathway to achieving ‘pores on demand.’ It allows us to pursue green and sustainable objectives while also enabling control over the fate of material structures—this is the core driving force for the future development of high-performance porous carbon materials.

Here, ‘engineered construction’ is used in a precise, non-rhetorical sense: it denotes the deliberate, template-directed fabrication of carbon architectures with predetermined pore size, geometry (cylindrical, spherical, or layered), spatial arrangement, and dimensionality, as opposed to the stochastic pore development of uncontrolled pyrolysis/activation. It is not intended to imply that every product is defect-free or ‘perfect’; rather, it signals that structure is programmed by design parameters (template identity, precursor, pyrolysis condition) instead of being left to thermodynamic chance. Where the original text used exaggerated wording (‘perfect integration’, ‘catalytic magic’, ‘remarkable structural-precision demonstrations’), these have been replaced with measured alternatives (‘synergistic integration’, ‘catalytic role’, ‘structural-precision demonstrations’) to avoid overstatement.

### 3.1 Principles of morphological engineering: comparison of hard template and soft template strategies

Based on the characteristics and operating mechanisms of the template method, materials morphology engineering is mainly divided into two major schools—the ‘hard template method’ and the ‘soft template method.’ Each follows different physicochemical principles and offers complementary advantages in preparation accuracy, process complexity, and applicability.

The hard template method can be regarded as ‘nano-scale precision casting.’ Its core lies in using rigid nanostructures with high thermal stability, such as ordered mesoporous silica (like SBA-15, MCM-48), anodic aluminum oxide membranes, and zeolite molecular sieves, as templates (Ghaedi and Zhao, 2022). This process is commonly referred to as ‘nano-replication’ or ‘nano-casting.’ In practice, a carbon precursor (such as sucrose, phenolic resin, or acrylonitrile) is impregnated into or filled into the template’s microchannels or gaps, followed by *in situ* polymerization and high-temperature curing to form a carbon framework (Asasian-Kolur et al., 2024). Finally, chemical etchants (such as hydrofluoric acid to dissolve silica (SiO_2_) templates or sodium hydroxide to, etch zeolites) are used to remove the solid template, leaving a highly ordered porous carbon structure that is the ‘negative’ or ‘inverse’ of the template’s morphology (Tan et al., 2023). The greatest advantage of this method is its extremely high structural fidelity—the microscopic size and morphology of the template can be almost perfectly replicated in the product, allowing the preparation of mesoporous carbon materials with a very narrow pore size distribution and highly ordered channels (Asasian-Kolur et al., 2024). For example, using MCM-48 or SBA-15 as a template can accurately replicate two-dimensional hexagonal or three-dimensional cubic mesoporous networks (Muñoz et al., 2025). However, this high fidelity comes at a high cost. The hard-template fabrication process is cumbersome and expensive: template synthesis often involves complex chemical reactions and strict condition controls, and the subsequent etching and removal steps are particularly hazardous (for example, hydrofluoric acid is highly corrosive and poses significant environmental hazards). Moreover, because the template pores are difficult to fill efficiently, yield is often low, and later recovery and handling of the template are also extremely challenging (Nath et al., 2025).

In contrast, soft templating is more like a molecular-level ‘self-assembly’ process. It utilizes amphiphilic block copolymers (such as F127, P123, etc.) or surfactants to form micelles, liquid crystal phases, and other dynamically ordered aggregates in solution as ‘soft’ templates (Figure 7a; Di Spirito et al., 2024; Kontogiannis et al., 2023). Unlike rigid solids with hard templates, soft templates are essentially volatile organic molecules (Chauhan, 2023; Hernik et al., 2025). During synthesis, the carbonization precursor (such as phenol-formaldehyde resin oligomers) interacts specifically with the hydrophilic or hydrophobic regions of the soft template, co-assembling into ordered nanocomposites (Figures 7b,c; Chen C. et al., 2024; Robertson et al., 2022). Figure 7b shows the microscopic mechanism of this process: template molecules and carbon precursors first self-assemble into ordered nanostructures through non-covalent interactions. Then, after cross-linking and high-temperature carbonization, the precursor transforms into a stable carbon framework, while the polymer template thermally decomposes, ultimately leaving highly ordered mesoporous channels in the carbon matrix (Robertson et al., 2022). Figure 7c uses the classic PEO-b-PPO-b-PEO triblock copolymer as a template and resol phenolic resin as the carbon precursor, further illustrating the specific intermolecular interactions in this cooperative assembly process (Robertson et al., 2022). Subsequently, during crosslinking pyrolysis at high temperatures, the precursor transforms into a porous carbon network. At the same time, the soft template decomposes or volatilizes during pyrolysis (usually removed directly), leaving behind the spatial imprint it once occupied (Huang J. et al., 2023; Suresh et al., 2025). The advantage of the soft templating method lies in its simple process and low cost—it does not require complex solid etching steps, and by adjusting the structure, concentration, or solvent environment of the template molecules, the pore size can be flexibly controlled (for example, changing the micelle size by adjusting the surfactant concentration). However, this flexibility also brings uncertainty in structural control. Since the assembly of soft templates relies on weak intermolecular interactions (such as hydrogen bonding and hydrophobic effects), they are highly sensitive to synthesis conditions (such as pH, temperature, and concentration), and during high-temperature carbonization, soft templates may decompose or migrate prematurely, resulting in the final product’s order (especially long-range order) being generally less perfect than that of the hard templating method (Zhang X. et al., 2024).

**FIGURE 7:**
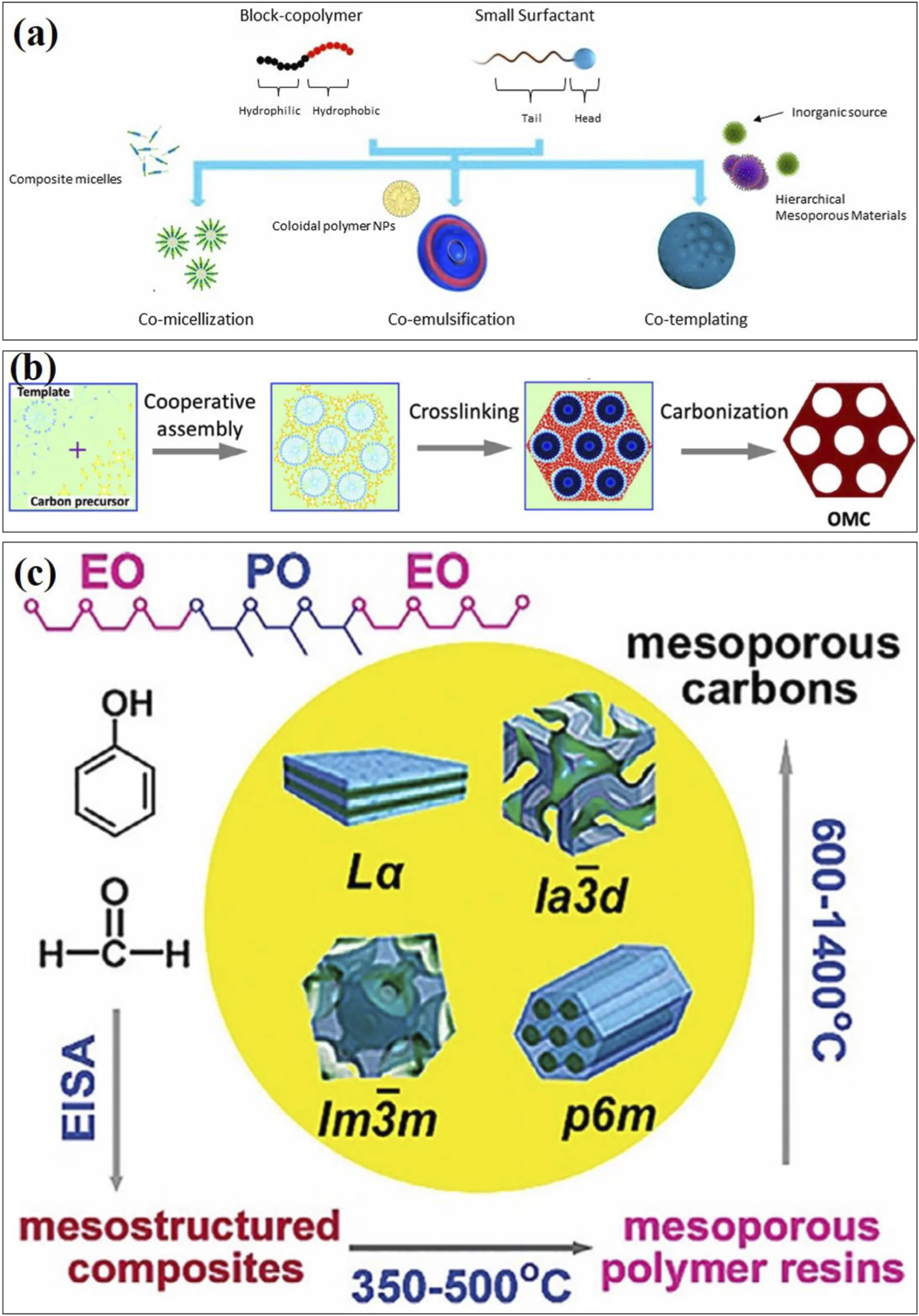
**(a)** Binary amphiphilic co-assembly of amphiphilic block-copolymers with small-weight surfactants (Kontogiannis et al., 2023). **(b)** Synthesis of MCs through an established soft-templating method. The cooperative assembly of template molecules and carbon precursors enables the formation of ordered nanostructures. After subsequent crosslinking and carbonization, the precursors are converted to a carbon matrix, while the polymer template is thermally decomposed to create pores (Robertson et al., 2022). **(c)** Synthesis of MC using linear block copolymer, PEO-b-PPO-b-PEO, as the template and resol as the carbon precursor (Robertson et al., 2022).

Therefore, both hard-template and soft-template methods have their own advantages. The hard-template method is like a master sculptor, capable of carving fine and precise structural details, making it suitable for research fields that require extremely high pore-structure accuracy. In contrast, the soft template method is like a painter, able to quickly and flexibly create diverse structural forms, making it suitable for large-scale production and cost-sensitive applications.

### 3.2 MOF-derived carbon: the synergistic integration of template and precursor

The advent of MOF-derived carbon marks a ‘paradigm-blending’ revolution in the development history of template synthesis processes. While traditional hard-template methods can provide precise pore structures using materials such as silica and graphene, they often require cumbersome post-synthesis etching to remove the template and struggle to achieve molecular-level chemical uniformity. In contrast, the self-assembly characteristics of MOF materials inherently give them a naturally crystalline porous framework structure (Figures 8a–d; Fonseca et al., 2023). Additionally, the insets in Figures 8a,b provide intermediate magnifications with scale bars of 5 μm and 1 μm, respectively, ensuring a seamless transition across the entire range of length scales (Fonseca et al., 2023). Field emission scanning electron microscopy images in Figures 8a–d, using TRD-ZIF-8 crystals as an example, reveal the intricate structure of this self-templating material: Figure 8a shows the three-dimensional ordered superstructure assembly at a macroscopic scale (200 μm) (Fonseca et al., 2023). Figures 8b,c show progressively magnified images (from 10 μm to 2 μm) clearly displaying the crystal’s regular rhombohedral shape (Fonseca et al., 2023). Figure 8d captures smooth and well-ordered crystal planes and clear grain boundaries on the single crystal surface at the 500 nm scale (Fonseca et al., 2023). This high degree of order from the macro to the nanoscale is the structural origin that allows MOFs to serve as ideal self-sacrificing templates, forming functional carbon materials with uniform chemical composition and an inheritable pore structure after pyrolysis. When we directly use MOFs as precursors for high-temperature pyrolysis, they simultaneously play the dual role of ‘template’ and ‘carbon/doping source.’ This unified dual identity cleverly combines the structural precision of the hard-template method with the molecular-level uniformity of the precursor method. It not only preserves the original porous and ordered structure of the MOF but also, through pyrolysis, transforms metal ions and organic ligands into functional heteroatom doping sites (such as N, S, P, etc.), thereby opening a unique and efficient pathway for preparing high-performance, multifunctional heteroatom-doped porous carbon materials (Nagappan et al., 2022; Xiao et al., 2023). This technology undoubtedly provides a promising new materials platform for fields such as energy storage, catalysis, and environmental management.

**FIGURE 8:**
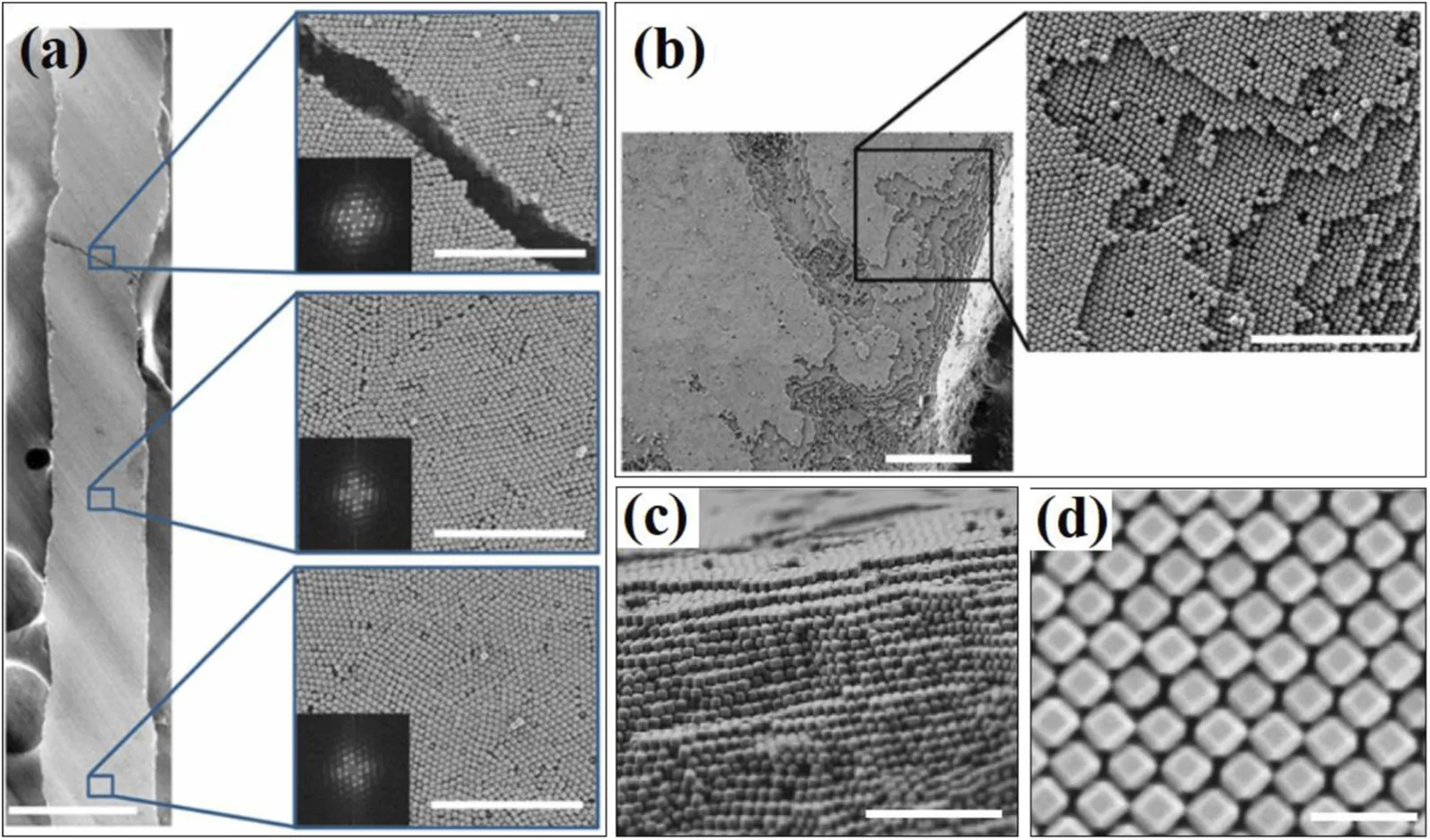
**(a**–**d)** FE-SEM images of a 3D ordered rhombohedral superstructure made of TRD-ZIF-8 crystals (t = 0.69). Scale bars: **(a)** 200 μm; (a, insets) 5 μm; **(b)** 10 μm; (b, inset) 1 μm; **(c)** 2 μm; **(d)** 500 nm (Fonseca et al., 2023).

First, the core of this technology lies in its ability to bridge the traditional hard-template method and the soft-precursor method, achieving perfect integration of the ‘template’ with the ‘carbon source/dopant source.’ The crystal structure of the MOF itself (such as ZIF-8, ZIF-67, etc.) provides a natural pore template, which can preserve a highly ordered porous structure after pyrolysis, drawing on the structural precision of the hard-template method (Figures 9a–c; Huang Y. et al., 2023; Singh and Yadav, 2025). Figures 9a,b show the crystal structures of ZIF-8 and ZIF-67, respectively. Both are assembled from metal ions (Zn or Co) and imidazole ligands through coordination bonds, forming a three-dimensional open framework with regular pores (Huang Y. et al., 2023). This intrinsic porous structure serves as the structural basis for the subsequent inheritance of pore channels in carbon materials. Figure 9c illustrates the pyrolysis process of ZIF-8 and ZIF-67 at temperatures above 1,000 °C: as the temperature rises, the organic ligands gradually carbonize into a carbon framework, the metal species undergo reduction or evaporation, while the original periodic pore structure of the MOF is partially preserved within the carbon matrix, ultimately forming functional carbon materials with both high specific surface area and ordered pore channels (Huang Y. et al., 2023). This “kill two birds with one stone” design strategy allows MOF-derived carbon to retain the structural regularity of the hard-template method while avoiding cumbersome template-removal steps and simultaneously achieving uniform *in situ* doping of heteroatoms. At the same time, the pyrolysis process is not only carbonization but also *in situ* chemical transformation, where metal nodes are converted into active sites (such as single-atom metals, metal oxides/sulfides), and organic ligands are transformed into heteroatom-doped porous carbon frameworks, reflecting the molecular-level uniformity of the precursor method (Guo et al., 2024; Li W. et al., 2025).

**FIGURE 9:**
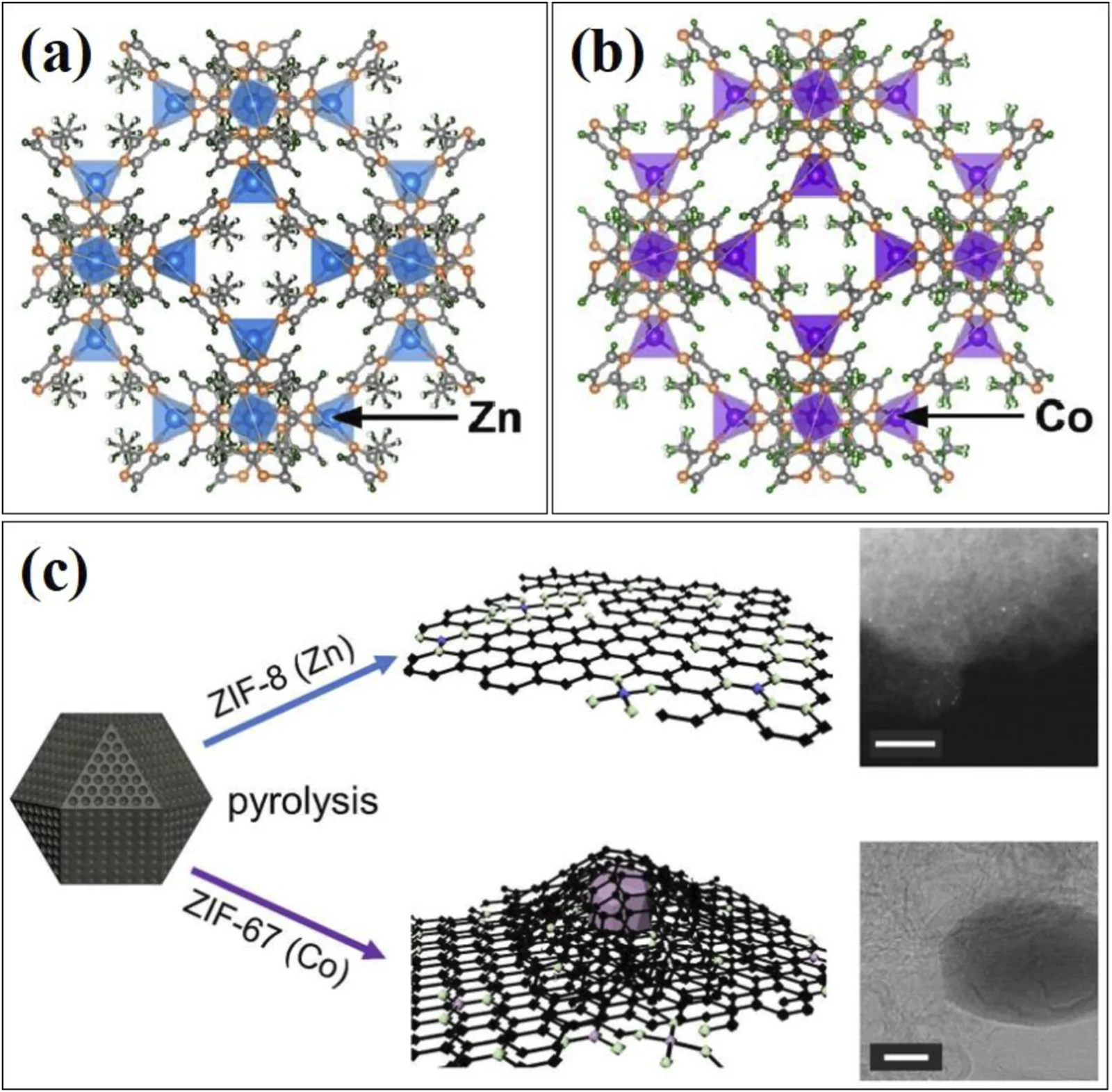
**(a)** The crystal structure of ZIF-8 (Huang Y. et al., 2023). **(b)** The crystal structure of ZIF-67 (Huang Y. et al., 2023). **(c)** Schematic figure on pyrolyzing ZIF-8 and ZIF-67 to over 1,000°C (Huang Y. et al., 2023).

In terms of synthesis pathways, technology has evolved from purely direct pyrolysis methods to various innovative approaches such as spatial confinement methods and post-treatment activation. For example, growing MOFs on specific substrates (such as silica gel or graphene) or introducing guest molecules (such as tannic acid) can achieve finer structural control through spatial confinement effects (Gutiérrez et al., 2022). Further increasing the specific surface area and pore volume can be achieved by removing metal residues through acid etching or alkaline activation (such as KOH treatment) (Williams et al., 2022). In terms of functionalization, MOF-derived carbon is unique for its heteroatom doping and active-site engineering (Figures 10a,b; Chai et al., 2025; Chen F. et al., 2024). Figure 10a systematically summarizes the structural models of various MOF-derived carbon materials, showing the carbon framework morphology and pore characteristics obtained after pyrolysis of different MOF precursors (Chai et al., 2025). Figure 10b further focuses on the configuration models of typical nitrogen species in porous carbon, providing a detailed presentation of the spatial arrangements of different doping forms such as pyridinic nitrogen, pyrrolic nitrogen, and graphitic nitrogen within the carbon framework. The types and proportions of these active sites directly determine the material’s functional performance in fields such as catalysis and energy storage (Chai et al., 2025). Metal ions (such as Co, Fe, Cu) can form single-atom metals or metal nanoparticles during pyrolysis, both of which are highly efficient electrocatalytic active sites (Gul et al., 2025; Zhang H. et al., 2022). Elements such as nitrogen, sulfur, and phosphorus in the organic ligands can be embedded *in situ* into the carbon framework, forming N-doped carbon (N-C), P-doped carbon (P-C), and so on, which significantly enhances conductivity and active-site density (Sielicki et al., 2024). The application prospects are broad. With its high specific surface area and conductivity, MOF-derived carbon performs excellently in energy storage. It can serve as a supercapacitor electrode material to provide excellent power density. It can also suppress polarization and improve cycle life in lithium-ion, sodium-ion, and lithium-sulfur batteries (Chai et al., 2025; Song et al., 2023). In environmental management and catalysis, it shows high efficiency in photoelectrocatalytic degradation of organic pollutants, heavy metal adsorption, and electrocatalytic water splitting (HER/OER) (Tian et al., 2025; Akande and Okolie, 2024). In addition, by regulating the dielectric loss of the carbon-based framework and the magnetic loss of magnetic metals, it can also achieve efficient microwave absorption performance and is widely used in electromagnetic wave absorption and electromagnetic shielding materials (Yang et al., 2022). Future research is rapidly shifting from ‘material preparation’ to ‘functional materials engineering.’ The focus is moving from simple carbonization to more complex structural design, such as using the spatial confinement effect of MOFs to uniformly disperse metal atoms on carbon-based supports to create highly active single-atom or dual-atom catalysts (Xing et al., 2023; Zulhaimi et al., 2025). Or explore composites of MOF-derived carbon with other materials (such as metal-organic frameworks or polymers) to achieve multifunctional integration.

**FIGURE 10:**
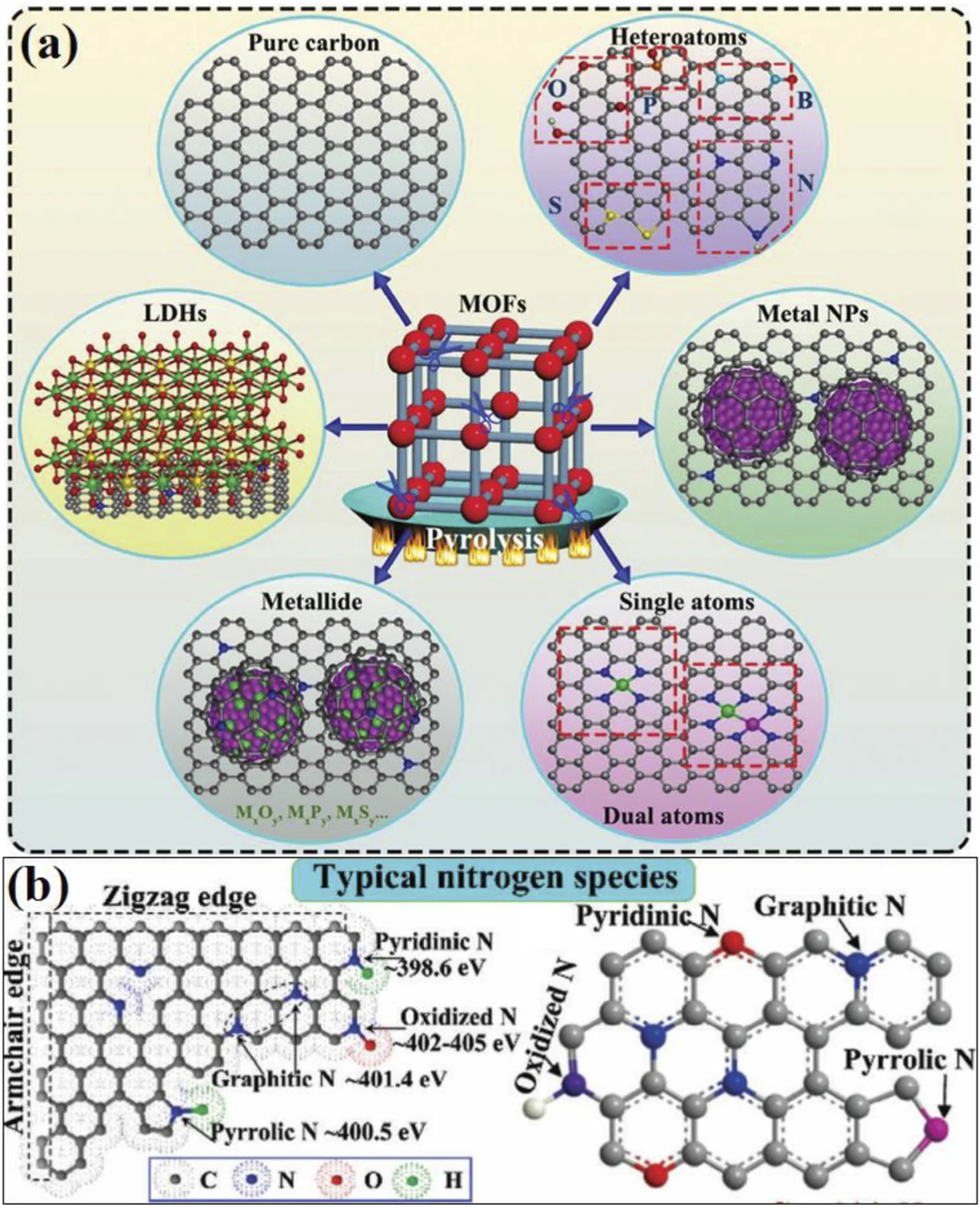
**(a)** Structural diagram of various CMs derived from MOFs (Chai et al., 2025). **(b)** Configuration models of typical N species in PC (Chai et al., 2025).

Quantitatively, MOF-derived carbons are among the highest-performing template carbons: direct carbonization of ZIF-8 typically yields N-doped porous carbon with specific surface area 1,000–1900 m^2^ g^-1^ and, as supercapacitor electrodes, specific capacitance of 185–290 F g^-1^ in aqueous electrolytes (e.g., ZIF-8-derived porous carbon: 1823 m^2^ g^-1^, 219 F g^-1^ in 1 M H_2_SO_4_ (Ahmad et al., 2020); N-doped ZIF-8-derived carbon: ∼286 F g^-1^ at 0.1 A g^-1^ in 6 M KOH (Ahmad et al., 2020)); ZIF-67-derived carbons reach 190–305 F g^-1^ (Ahmad et al., 2020). These metrics substantiate the performance claims that were previously only qualitative.

Therefore, MOF-derived carbon technology is rapidly developing, and its unique ‘dual identity’ characteristics offer a highly promising material solution for addressing energy and environmental crises.

Clarification of the ‘sustainability’ of MOF-derived versus biomass-derived carbon (addressing the reviewer’s concern). The two routes are sustainable in fundamentally different senses and should not be equated. Biomass-derived carbon is ‘sustainable’ primarily through feedstock circularity: it consumes renewable or waste feedstock (approx. 100% renewable). It is the most readily scalable of the four strategies (Maniscalco et al., 2020), but it inherits precursor heterogeneity and typically shows high reproducibility scatter (>15% RSD) and broad pore-size distributions (>2 nm FWHM) (Hamidu et al., 2025; Razzak, 2024). By contrast, MOF-derived carbon is ‘sustainable’ in the sense of structural precision and atomic-level uniformity, not feedstock circularity: MOFs are assembled from petrochemical-derived organic linkers (e.g., 2-methylimidazole) and metal salts (Zn/Co nitrates), exhibit low carbon yield (13–21 wt% of precursor) (Ding et al., 2022), and require energy-intensive pyrolysis (>800–1,000 degC) plus acid/alkali leaching of metal residues (Xie et al., 2018). Consequently, the two are complementary rather than equally ‘green’: biomass offers renewable feedstock and scalability, whereas MOF/template routes offer precision and uniformity at higher cost and lower yield. The text has been revised to avoid implying that MOF templating is intrinsically as sustainable as biomass sourcing.

#### 3.2.1 Source of advantage: from molecular design to the inheritance and transformation of carbon structures

The outstanding performance of MOF-derived carbon is rooted in the unparalleled designability of its precursors. This designability is exquisitely ‘inherited’ by the derived carbon after pyrolysis, enabling a perfect translation from atomic-level formulation to material-level properties. Firstly, MOFs themselves possess atomic-level precise periodic crystal structures and well-defined, uniform micropores (sometimes including mesopores). This ‘memory effect’ of ordered porosity is preserved during pyrolysis (Figures 11a–e; Asgari et al., 2025; Kim M. et al., 2024; Zhang G. et al., 2025). As shown in Figures 11a–e, this genetic effect manifests on multiple levels: first, the MOF has an atomically precise periodic crystal structure, as well as well-defined, uniform micropores (sometimes including mesopores) (Asgari et al., 2025; Zhang G. et al., 2025). This ordered porous “memory effect” allows the derived carbon to partially retain the precursor’s structured pore characteristics even after high-temperature pyrolysis. Figure 11a, taking heterogeneous catalysis as an example, demonstrates the excellent performance of MOF-derived cobalt nanocatalysts in amine synthesis—the high specific surface area ensures sufficient reactive sites per unit volume. At the same time, the customizable porous structure facilitates rapid transport of reactants and products (Asgari et al., 2025). Figure 11b further expands this concept, illustrating the synthesis pathways of MOF-derived structures in different dimensions (zero-dimensional, one-dimensional, two-dimensional, and three-dimensional), highlighting the guiding effect of precursor morphology on the final product morphology (Zhang G. et al., 2025). Figures 11c–11e show a series synthesized through the isostructural framework of MOF-74 spheres, demonstrating how the pore size can be precisely controlled by adjusting the synthesis conditions of the precursors—this precursor-level tunability of the pores is ultimately inherited by the derived carbon materials, enabling on-demand customization of the pore structure (Zhang G. et al., 2025). Although ligands decompose and parts of the framework collapse, the initial topology and spatial profile of the MOF are often ‘remembered.’ During pyrolysis, metal ions/clusters may transform into metal/metal oxide/carbide nanoparticles, which can serve not only as ‘self-templates’ but also as catalysts to catalyze the formation of additional pores around them. Eventually, while inheriting the main framework of the parent MOF, a hierarchically porous carbon structure rich in secondary pores derived from metal species is formed, achieving an extremely high specific surface area (often exceeding 2000 m^2^/g) and tunable pore hierarchy.

**FIGURE 11:**
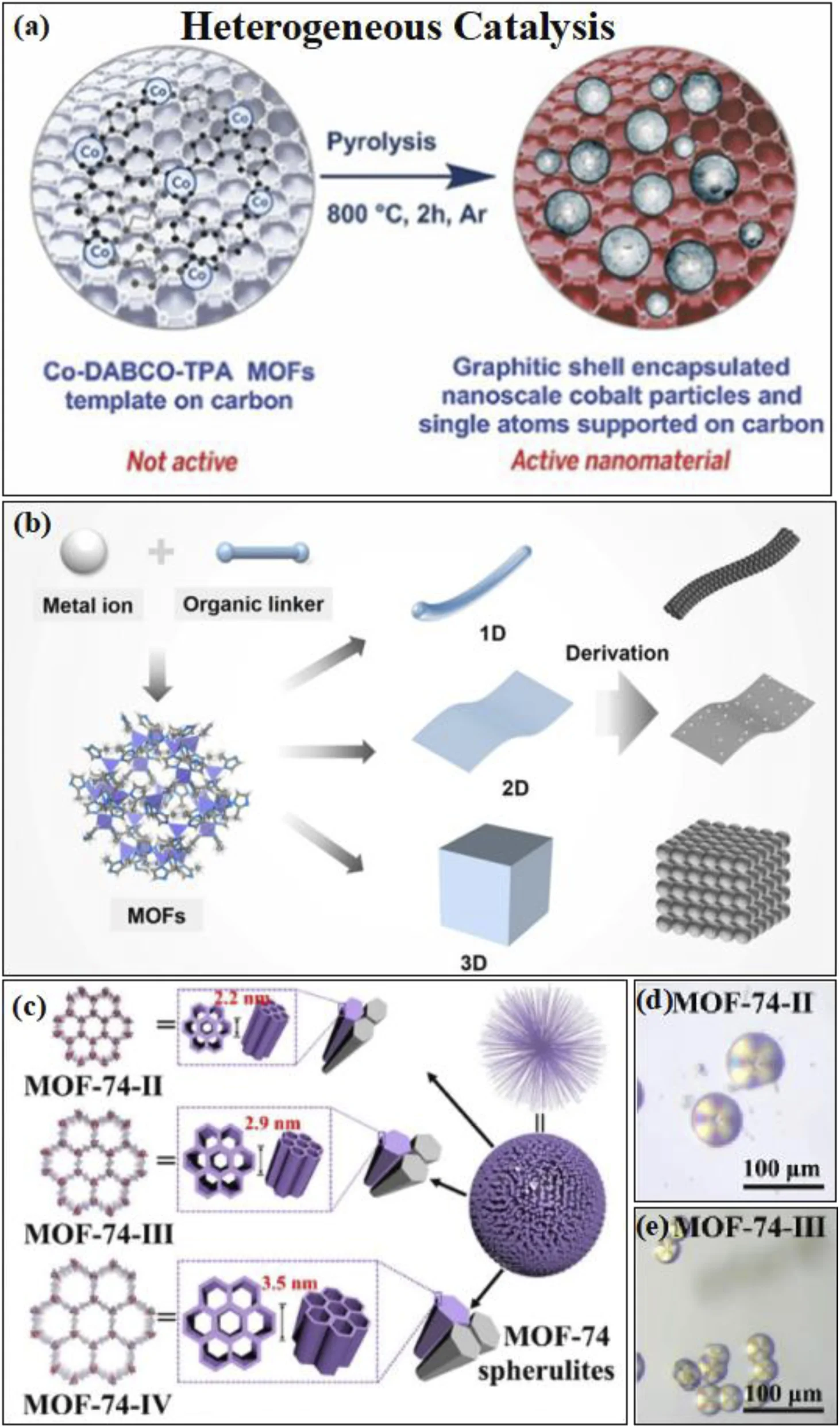
**(a)** Heterogeneous catalysis: high surface areas allow high reaction rates per unit volume, and the tailorability of the porous structure allows rapid transport of reactant and product molecules. Here, we present the design of a MOF-based cobalt nanoparticle that catalyses the synthesis of amines (Asgari et al., 2025). **(b)** Synthesis diagram of MOF-derived structures in different dimensions (Zhang G. et al., 2025). **(c**–**e)** Isoreticula synthesis of MOF-74 spherulites with tunable pore sizes (Zhang G. et al., 2025).

Secondly, the organic ligands in MOFs are molecular-level carbon sources uniformly distributed in three-dimensional space, and this composition and doping uniformity are difficult to achieve with physically mixed dopants (Gong et al., 2022; Wang et al., 2026). When the ligand itself contains heteroatoms such as nitrogen, sulfur, or phosphorus, these heteroatoms can be uniformly doped into the carbon framework after pyrolysis as atomic-level dispersion or specific chemical states, forming intrinsic, high-density, uniformly distributed active sites (Figures 12a–c; Majumdar et al., 2024; Zhang H. et al., 2025). Figure 12a, which illustrates the preparation of Fe-ADMC/MIL101-T, demonstrates a general strategy for constructing single-atom catalysts using heteroatom-containing organic ligands: by selecting appropriate ligand molecules (such as nitrogen-containing imidazoles or sulfur-containing carboxylic acids), heteroatoms can be uniformly introduced into the framework during MOF synthesis. After high-temperature pyrolysis, these heteroatoms are *in situ* converted into nitrogen-doped or sulfur-doped sites, while the metal ions are reduced to atomically dispersed single-atom active centers (Zhang H. et al., 2025). Taking CoSAs@NC-920 as an example, Figure 12b presents the complete preparation process of the cobalt single-atom catalyst in detail—nitrogen-containing ligands self-assemble with cobalt salts to form a ZIF-type precursor. After pyrolysis above 900 °C, the cobalt atoms are stably anchored on the nitrogen-doped carbon substrate in the form of Co-N coordination, achieving uniform dispersion at the single-atom scale (Zhang H. et al., 2025). Figure 12c shows the synthesis route of Fe-SN-C. By introducing a bifunctional ligand containing sulfur and nitrogen, a sulfur/nitrogen co-doped carbon-supported single-atom iron catalyst was successfully prepared. This multi-element co-doping strategy provides greater flexibility for tuning the electronic structure of the metal center and optimizing catalytic performance (Zhang H. et al., 2025). For example, using nitrogen-rich ZIF-8 as a precursor, one can directly obtain N-doped porous carbon with high nitrogen content, existing in various forms such as graphitic nitrogen and pyridinic nitrogen. This material exhibits excellent electrocatalytic performance (Jiao et al., 2023; Tang et al., 2024; Zhang W. et al., 2024). Therefore, MOF-derived carbon achieves precise conversion from ‘formulation’ to ‘performance’ by preserving the orderliness of the topological structure and the uniformity of ligand doping, demonstrating a high degree of design freedom and application potential.

**FIGURE 12:**
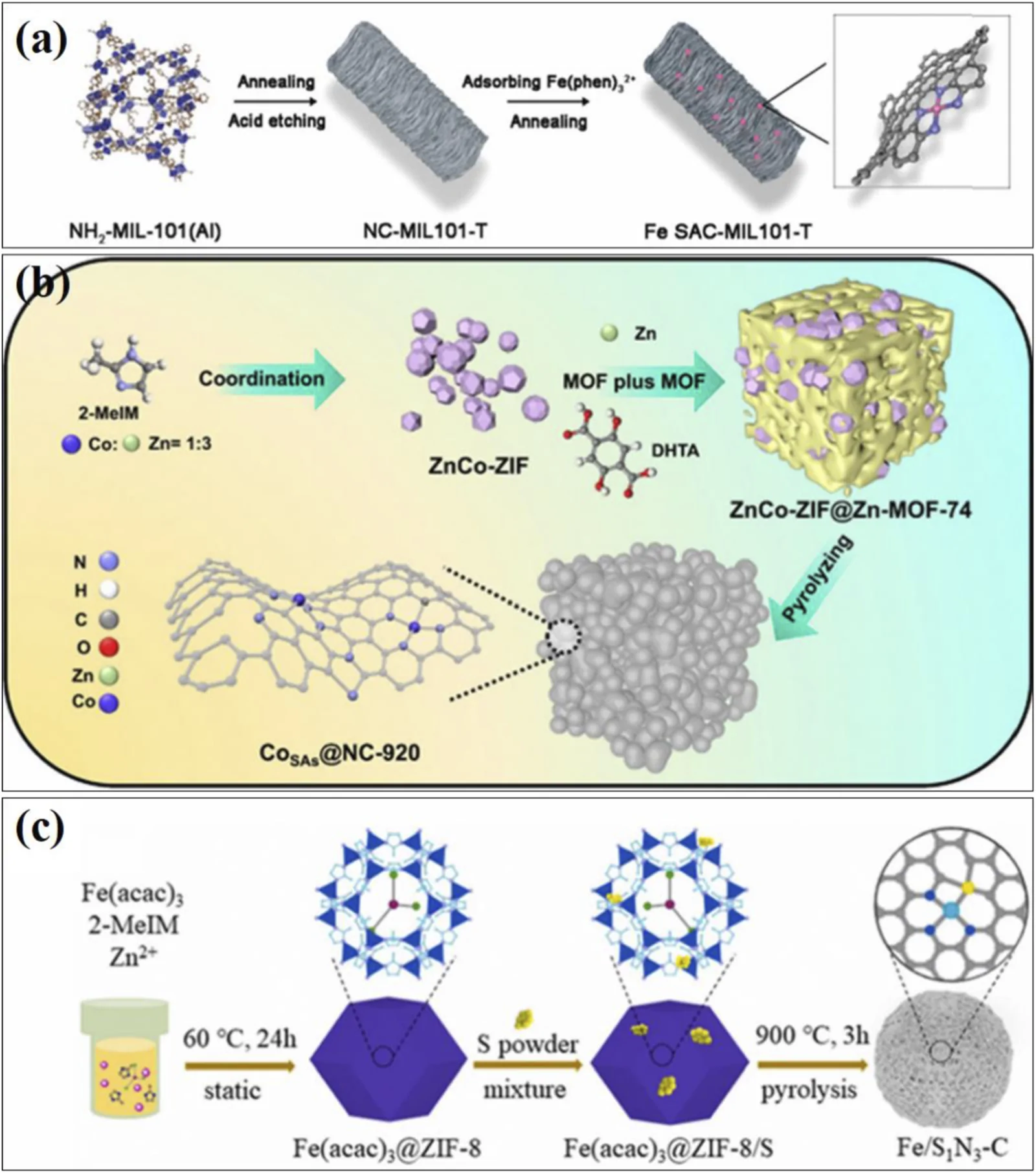
**(a)** Schematic diagram of the preparation of Fe ADMC MIL101-T using organic ligands (Zhang H. et al., 2025). Synthesis methods for single-atom catalysts: CoSAs@NC-920 and Fe–SN–C. **(b)** Illustration of the synthetic procedure of CoSAs@NC 920 (Zhang H. et al., 2025). **(c)** Scheme of the synthesis of Fe–SN–C (Zhang H. et al., 2025).

#### 3.2.2 Pyrolysis: the key to structural evolution and the catalytic role of metal nodes

This stage marks a critical turning point from the ‘precursor’ to the ‘final product,’ with metal nodes playing a catalytic role like that of a ‘magician.’ In the early stage of pyrolysis, organic ligands mainly undergo decomposition and gasification, generating volatile small molecules, and ultimately leaving behind a carbonaceous framework rich in metal ions (Figure 13a; Iliescu et al., 2023; Pérez-Mayoral et al., 2023; Tian et al., 2022). As the temperature rises further, the metal nodes enter the critical ‘catalytic graphitization’ stage (Goldie and Coleman, 2023). Using Co and Fe as examples, once these metal ions or metal clusters are reduced to the zero-valent state at high temperatures, they strongly catalyze the rearrangement of the surrounding amorphous carbon, promoting its transformation into highly ordered graphitized carbon (Figures 13b–d; Cao et al., 2025; Mennani et al., 2024). Zn, however, does not belong in this group: because metallic Zn boils at only 907 °C, the Zn nodes of ZIF-derived precursors are reduced and then volatilized during high-temperature pyrolysis, so that Zn acts predominantly as an evaporative porogen that generates micro- and mesoporosity rather than as a graphitization catalyst (Xing et al., 2026); *in situ* heating TEM further shows that trace Zn persists as atomically dispersed Zn-N sites even at 1,100 °C (Xing et al., 2026). Figure 13b illustrates the microscopic mechanism of this process: at high temperatures, the metal catalyst migrates, and when carbon atoms dissolved and diffused on the surface of the metal particles reach supersaturation, they precipitate as highly ordered graphite carbon, forming a graphitized nanostructure (Mennani et al., 2024). The high-resolution transmission electron microscopy image in Figure 13c provides intuitive evidence for this mechanism—the image clearly shows the interfacial features where the metallic carbide structure induces graphite growth. The graphite layers grow radially or concentrically around the metal particles, confirming the metal-catalyzed directional graphitization (Mennani et al., 2024). Taking the process flow for preparing graphitizable carbon materials from coconut shells as an example in Figure 13d, it further demonstrates the application potential of this catalytic graphitization mechanism in practical biomass conversion—by reasonably selecting metal catalysts and regulating pyrolysis conditions, efficient conversion from low-cost biomass precursors to high-value graphite carbon materials can be achieved (Mennani et al., 2024). This metal-catalyzed graphitization not only greatly enhances the electrical conductivity of the final material but also improves its chemical stability, enabling excellent performance in electrochemical and catalytic applications (Mennani et al., 2024). At the same time, the behavior of metal nanoparticles during the pyrolysis process is also a key factor in determining the final pore structure (Muzyka et al., 2023). They may agglomerate, migrate, react with the carbon matrix to form metal carbides, or even evaporate at high temperatures (such as Zn). The dynamic behavior of these metals directly influences the formation and distribution of mesopores and macropores (Han et al., 2022). Therefore, by precisely controlling the temperature profile, heating rate, and atmospheric composition during pyrolysis, it is equivalent to ‘programming’ the evolution path of the metal nodes.

**FIGURE 13:**
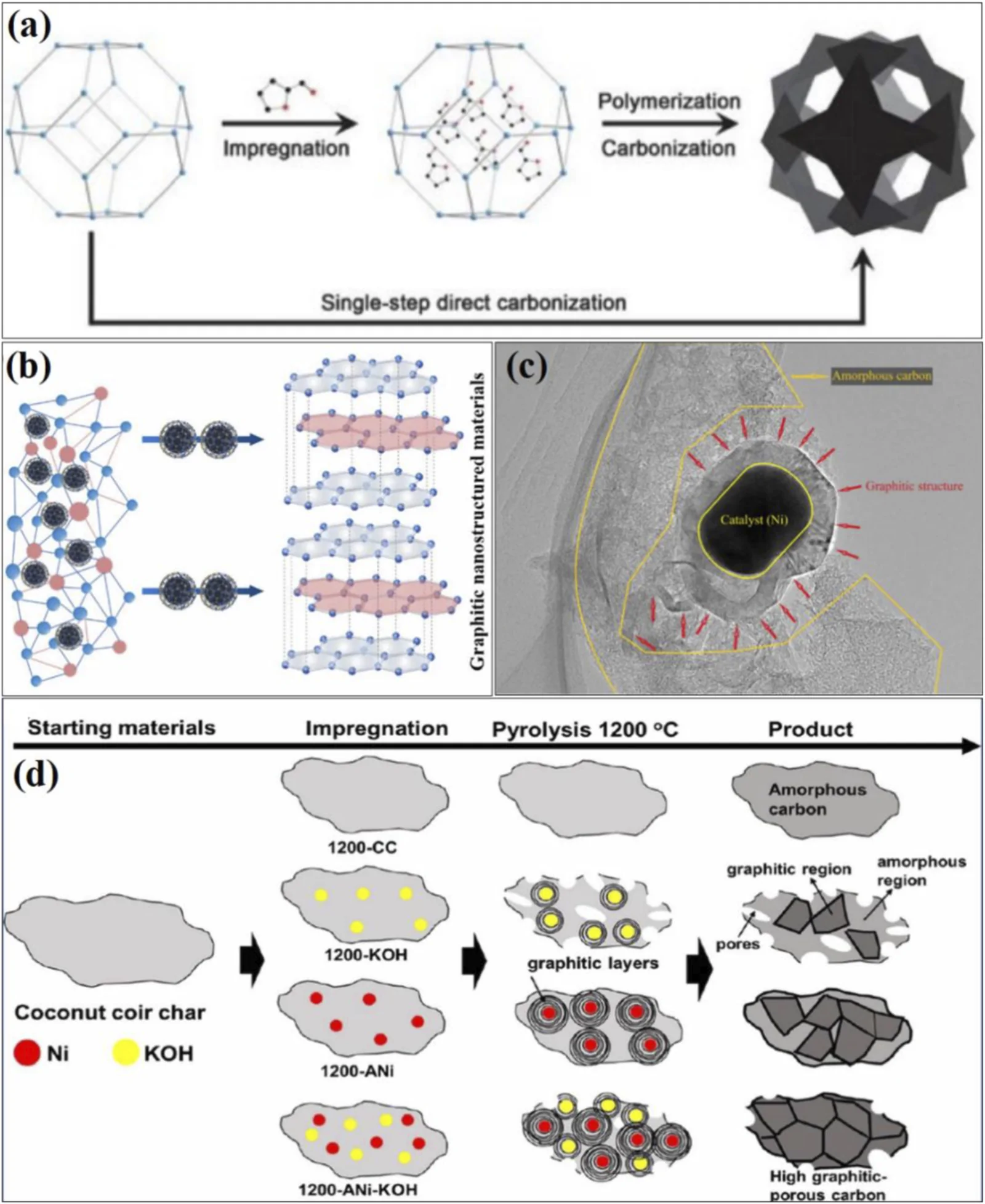
**(a)** Schematic representation of porous carbons from MOFs with furfuryl alcohol as an additional carbon source and direct carbonization of MOFs (Pérez-Mayoral et al., 2023). **(b)** Formation of graphitic nanostructures through migration of catalysts and precipitation of ordered carbon (Mennani et al., 2024). **(c)** HRTEM image of metal-catalyzed graphitization; the metal carbide structure prompted the growth of graphite (Mennani et al., 2024). **(d)** Process mechanism for the synthesis of graphitizable carbonaceous materials from coconut palms. Reprinted with permission (Mennani et al., 2024).

This highly controllable structural evolution capability allows us to prepare derivative carbon materials for specific applications from the same type of MOF precursor by fine-tuning the pyrolysis process (Chen and Qian, 2024; Gong et al., 2023). For example, for supercapacitor materials that require high conductivity and rapid ion transport, this can be achieved by optimizing metal-catalyzed graphitization (Ma X. et al., 2025). For materials used in oxygen reduction catalysis (ORR) or lithium-sulfur battery carriers, which require specific metal active sites and pore structures, this can be achieved by controlling the chemical state of the metal (such as retaining the metal in its elemental, oxide, or carbide form) as well as the size distribution of the pores (Chen L. et al., 2024; Ren et al., 2023).

### 3.3 Limitations and trade-offs: the cost of precision

Although the template method and metal-organic framework (MOF)-derived carbons are considered ‘remarkable structural-precision demonstrations’ in materials science for their structural precision, a series of costly ‘triangular walls’ blocks their industrial application.

First, in terms of the “original cost” of the materials, this route is extremely narrow. Whether using hard templates like ordered mesoporous silica or relying on costly MOF precursors, this route requires expensive reagents and strict control of conditions. The hard template method requires the template’s pore distribution to match the precursor at the microscopic level, resulting in complicated, time-consuming synthesis steps and very low yields (Ahmed et al., 2025; Li Z. et al., 2023). The cost issue of MOF-derived carbon is even more significant, as the synthesis of high-performance MOFs often involves expensive organic ligands (such as imidazole and terephthalic acid derivatives) and metal salts (such as cobalt and zinc salts), and large-scale green synthesis remains a major challenge (Shahzadi et al., 2024). Next is the hidden cost of the ‘post-processing cleaning’ stage. The hard template method usually relies on highly toxic and strongly corrosive reagents such as hydrofluoric acid (HF) to remove the silica template, which not only poses significant safety risks to workers but also creates serious environmental management challenges (Ghaedi and Zhao, 2022). If non-target metal nanoparticles remain during MOF-derived carbon preparation, leaching of these metals can not only cause environmental pollution but also trigger side reactions in applications such as batteries, requiring additional chemical removal and further increasing process complexity (Shakeel et al., 2025). Furthermore, there is a bottleneck in terms of ‘capacity expansion.’ Strategies based on single-crystal MOFs or complex nano-casting can usually be realized only at gram- or even milligram-scale laboratory levels (Peng et al., 2026). As the reaction is scaled up, ensuring uniform filling of precursors within the pores of hard templates or uniform distribution of MOF crystals becomes a major engineering challenge. Structural collapse, anisotropic shrinkage, and loss of pore-structure uniformity during pyrolysis are common and difficult problems in large-scale production. Finally, from a macroscopic, ‘life-cycle’ perspective, the so-called ‘green’ attributes are often compromised. Although MOF-derived carbons perform well in electrochemical energy storage and environmental remediation, their synthesis is often highly energy-intensive and involves toxic reagents (Chai et al., 2025; Shahzadi et al., 2024). A cradle-to-grave life cycle assessment shows that MOF production is significant in terms of energy consumption and carbon footprint, and complete data on their long-term degradability and recyclability are lacking (Singh et al., 2025). Therefore, compared to direct pathways that utilize waste biomass, its ‘green’ advantages have yet to be verified.

A quantitative comparison clarifies the “cost of precision” (Table 3). In hard-template routes, ordered mesoporous silica must be synthesized and then removed with highly toxic, corrosive hydrofluoric acid (HF), generating hazardous fluoride waste that requires special handling (Wang Z. et al., 2023). In MOF-derived routes, the precursor is the dominant cost driver: high-performance MOFs rely on expensive organic ligands (imidazoles, terephthalic-acid derivatives) and metal salts, and large-scale green MOF synthesis remains challenging (Shahzadi et al., 2024). Crucially, the productivity of MOF-derived carbon is low—typically only 13–21 wt% of the MOF precursor is recovered as useful carbon after pyrolysis and metal leaching (Ding et al., 2022)—so the effective carbon yield per kilogram of precursor is an order of magnitude below that of biomass pyrolysis. Energy demand is also high: MOF-derived carbon requires high-temperature pyrolysis (often >800 °C–1,000 °C) plus an additional acid/alkali leaching step to remove residual metal, and most MOFs (except Zn-based) need corrosive post-treatment that raises both cost and safety risk (Xie et al., 2018). Scale-up is presently limited to gram-to-milligram laboratory quantities because uniform pore filling of hard templates or uniform MOF crystal growth is difficult to maintain as reactor size increases (Peng et al., 2026). Taken together, template/MOF methods trade 10–100× higher reagent cost and toxic waste streams for structural precision that biomass routes cannot yet match.

**TABLE 3:** Economic and environmental trade-offs of precision synthesis routes for porous carbons.

Dimension	Hard template	MOF-derived carbon	Soft template
Typical carbon yield	Low (template mostly discarded)	13–21 wt% of precursor (Ding et al., 2022)	Moderate–high
Key cost driver	Silica synthesis + HF removal	Expensive ligands/metal salts (Biswal et al., 2024; Ding et al., 2022)	Surfactant/polymer cost
Toxicity/removal step	HF (highly toxic, corrosive) (Ghaedi and Zhao, 2022)	Acid/alkali leaching of metal (Xie et al., 2018)	Mild (often removable)
Energy demand	Pyrolysis + template removal	Pyrolysis >800 °C–1,000 °C + leaching	Pyrolysis/assembly
Scale-up status	Gram scale, lab (Peng et al., 2026)	Gram–milligram, lab (Ding et al., 2022; Peng et al., 2026)	Gram–pilot

Therefore, although the template method and MOF-derived carbons represent the scientific pinnacle of ‘top-down’ rational design of carbon material structures and provide unparalleled model materials for understanding the relationship between structure and performance, to transform them from ‘laboratory artworks’ into ‘industrial necessities,’ future research must focus on the following: first, developing low-cost, recyclable green templates and precursors to break the dependence on expensive reagents (Nanda and Kadja, 2024). Second, design a simplified process for one-step synthesis and non-corrosive post-treatment to solve the problem of removing highly toxic templates (Wu et al., 2026). Third, address structural collapse during scale-up through process innovation (Jansen et al., 2023). Fourth, establish a strict full life-cycle assessment system to ensure that the pursuit of ultimate performance does not deviate from the fundamental principles of sustainable development.

## 4 Process revolution: new physical technologies for extreme compression of energy and time

This energy transfer method has significant inherent drawbacks: the large thermal inertia results in a long heating time of several hours to several days (it may take tens of minutes or even hours to reach 3000 K), and severe heat losses due to radiation and convection lead to extremely low overall energy utilization efficiency, becoming the biggest bottleneck in achieving green manufacturing (Eze et al., 2024; Reveshti et al., 2026). Flash Joule Heating (FJH) and Microwave-Assisted Synthesis represent two “extreme” approaches to this core process that disrupt the traditional heating paradigm by introducing entirely new energy-coupling mechanisms (Tan et al., 2026). Flash Joule heating is an extreme non-equilibrium synthesis technique that utilizes the Joule effect. Unlike conventional tube furnaces, it does not rely on an external heat source for slow heat transfer; instead, it directly converts conductive precursors into thermal energy by applying instantaneous current pulses at high current density (>1,000 A/mm^2^) (Xiao et al., 2025). This “internal combustion” approach enables ultra-rapid heating (temperature ramp rates reach ≈10^5^ K s^-1^, i.e., the sample exceeds 3000 K within ∼100 m (Luong et al., 2020)), compressing reaction times from hours to milliseconds–seconds (Luong et al., 2020). Because the heat is deposited directly into the conductive precursor, thermal losses to the surroundings are minimal and the effective energy efficiency is far higher than that of a tube furnace; for flash graphene the electric energy cost is only ≈7.2 kJ g^-1^ (high-carbon feedstock) to ≈23 kJ g^-1^ (plastic waste), which is 2–3 orders of magnitude below conventional CVD (Algozeeb et al., 2020; Lacerda et al., 2023a).

### 4.1 Flash joule heating: a revolutionary thermodynamic approach

Flash Joule Heating (FJH) is a revolutionary thermodynamic approach that, thanks to its unique mechanism of instantaneous ultra-high current, achieves a fundamental breakthrough in energy input methods (Tan et al., 2026). This technique applies an instantaneous high current to a conductive precursor (or a conductive medium mixed with the precursor), using the Joule effect to generate immense heat and achieve ultra-fast heating and quenching of the material (Figures 14a–c; Xiao et al., 2025; Yuan et al., 2026). This unique instantaneous ultrahigh-current mechanism allows FJH technology to achieve rapid material structural transformations at extremely high heating rates (≈10^4^–10^5^ K s^-1^; the sample exceeds 3000 K within ∼100 m (Algozeeb et al., 2020; Yuan et al., 2026)) and very short processing times (milliseconds to a few seconds (Luong et al., 2020)), avoiding the energy consumption and side reaction issues associated with conventional long-duration high-temperature treatments.

**FIGURE 14:**
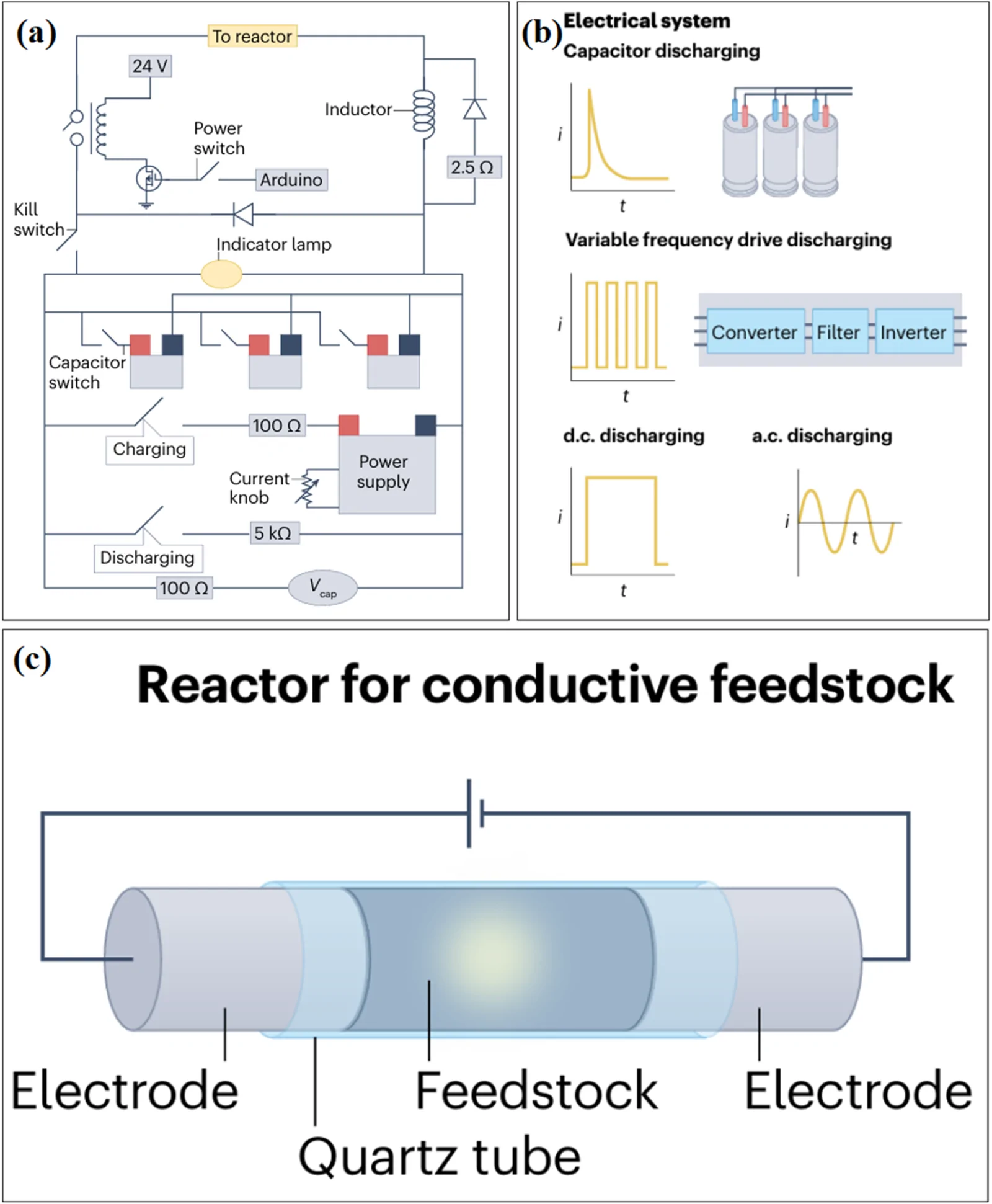
**(a)** Electronic diagram of a capacitor-based FJH system (Deng et al., 2025). **(b)** Four types of electric systems for current input: capacitor discharging for a single-pulse discharge; variable frequency drive enabling discharging with pulse-width modulation; constant d.c. discharging; and a.c. discharging. Capacitor discharging provides a higher but time-limited power input compared with a.c. or d.c. discharging; variable frequency drive provides an ultrashort pulse input, down to milliseconds; d.c. discharging and a.c. Discharging enables continuous heating, but the power is limited (Deng et al., 2025). **(c)** FJH reactor designs. For conductive feedstocks or mixtures of non-conductive feedstocks with conductive additives, current directly passes through the materials for direct heating (Deng et al., 2025).

#### 4.1.1 Principle: non-equilibrium transformation caused by ultrafast heating and quenching

The core physical picture of FJH is a complete departure from thermodynamic equilibrium paths (Deng et al., 2025). When a direct current pulse of several thousand amperes passes through the precursor in milliseconds or even sub-millisecond time, the internal resistance of the material generates intense Joule heating, causing its temperature to soar at an astonishing rate of over 100,000  K per second (>10^5^ K/s), instantly reaching high temperatures of 1,500–3,000 °C (Xiao et al., 2025). This heating rate is 5–6 orders of magnitude faster than that of a traditional furnace. Immediately afterward, when the current stops, the material cools (quenches) extremely quickly (∼10^4^ K/s) because of the large temperature difference between the sample and its surroundings and the sample’s low heat capacity. This “lightning-fast” heating and cooling process instantaneously pushes the reactant system into an extremely high-temperature state and quickly “freezes” it before it has enough time to relax to equilibrium. This leads to non-equilibrium thermodynamics dominating, making many reaction pathways feasible that, under conventional slow pyrolysis, are either kinetically inaccessible or require extremely high activation energies. For example, FJH can directly convert low-cost carbon-containing precursors (such as coal, biochar, waste tires, or even mixed plastics) into flash graphene within milliseconds. The reaction pathway may involve instantaneous rearrangement of carbon atoms and explosive ejection of heteroatoms, producing turbostratic graphene with unique defect structures (such as pentagons, heptagons, and edge sites) and high conductivity, which is fundamentally different in structure from graphene obtained through conventional chemical vapor deposition or long-term high-temperature annealing (Figures 15a–d; Advincula, 2022; Hosny et al., 2025). Figures 15a–d reveal the effects of FJH process parameters on product structure through the Raman spectroscopy system (Advincula, 2022). Figures 15a,b respectively show the effects of pulse voltage and pulse duration on the Raman characteristic peaks (D peak, G peak, 2D peak) of flash graphene using carbon black (CB) and scrap tire-derived carbon (SRT) as raw materials—as the voltage increases or the pulse duration is prolonged, the intensity ratios and shapes of the G and 2D peaks change significantly, reflecting the evolution of the degree of graphitization and the number of layers (Advincula, 2022). Figures 15c,d use thermally cracked carbon black (TCB) as the raw material, further demonstrating the universal ability of FJH parameters to modulate graphene structure—by optimizing pulse voltage and duration, an optimal balance between defect density and conductivity can be found through the intensity ratio of the D peak (defect indicator) to the G peak (graphitization indicator) (Advincula, 2022).

**FIGURE 15:**
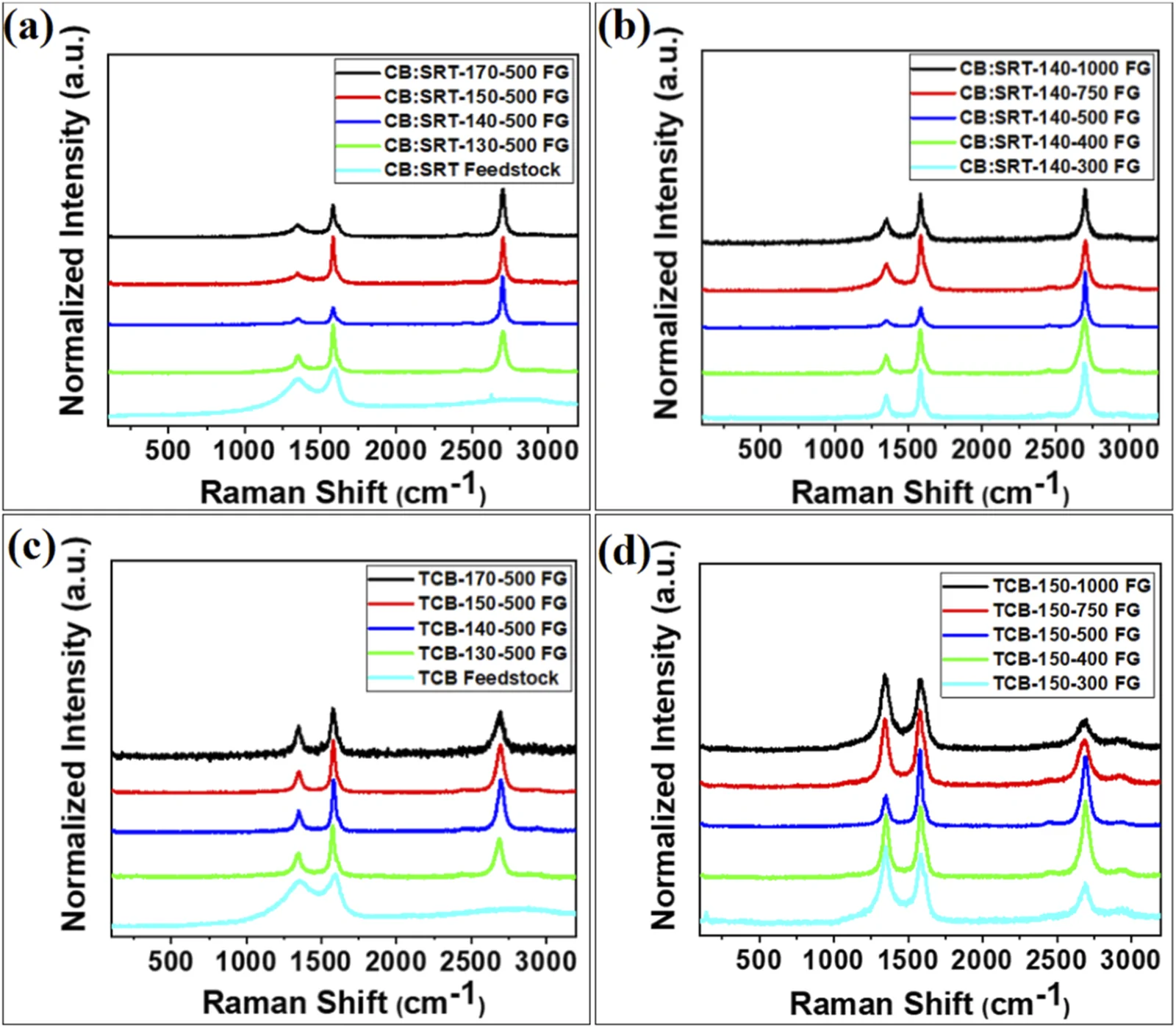
Raman spectral analysis based upon FJH parameter changes. The label format “Sample-X-Y FG” is used, where “Sample” is the carbon feedstock, X is the pulse voltage in V, Y is the pulse time in ms, and FG is tFG (Advincula, 2022). Raman spectra of CB:SRT tFG with varying **(a)** pulse voltages and **(b)** pulse times. Raman spectra of TCB tFG with varying **(c)** pulse voltages and **(d)** pulse times. A 532 nm laser with ×50 magnification is used to acquire Raman spectra (Advincula, 2022).

#### 4.1.2 Revolutionary advantage: Reshaping manufacturing logic

Quantitatively, flash Joule heating compresses synthesis from hours to sub-second: gram-scale flash graphene from carbon feedstocks is produced at a net energy of approximately −7.2 kJ g^-1^ (exothermic) and flash graphene upcycling from plastic waste at approximately −23 kJ g^-1^, with life-cycle CO_2_ emission reductions of 39%–84% versus incumbent routes under renewable-energy assumptions (Algozeeb et al., 2020; Silva, 2025; Wyss et al., 2023); microwave-assisted deconstruction of comparable plastic waste required approximately 4888 MJ in a direct comparative LCA, illustrating the relative energy economy of the two physical routes (Wyss et al., 2023; Zou et al., 2025). These metrics replace the earlier qualitative ‘rapid and energy-saving’ claim with explicit numbers.

Based on its unique physical principles, FJH demonstrates a range of advantages that overturn conventional understanding. This technology is not merely a heating method, but a revolution in manufacturing thinking.

##### 4.1.2.1 Omnipotent adaptability of precursors

Traditional high-temperature synthesis processes usually have very high requirements for the purity, crystallinity, and morphology of raw materials (for example, CVD graphene requires high-purity gases, while pyrolytic carbonization requires high-purity organic substances) (Yu and Cao, 2024; Zhang et al., 2023). FJH has completely broken this barrier, allowing almost all solid carbon-containing substances (regardless of their crystallinity or composition) to be used as raw materials (Hosny et al., 2025). As long as it has a certain level of conductivity, or by simply mixing in conductive additives such as carbon black (without complex chemical modification), it can be processed directly (Figures 16a–d; Blachowicz et al., 2023). Figure 16a takes the preparation process of milled carbon fiber/silicone rubber (MCF/SR) composites as an example to show how FJH technology can be combined with composite processing techniques—by adding a silicone rubber diluent (ST) to adjust the rheology of the system, enabling the conductive filler to be uniformly dispersed in the elastic matrix, thus laying the groundwork for subsequent FJH treatment or direct application (Blachowicz et al., 2023). Figure 16b shows the tool path planning for printing, illustrating how precise control of patterned composite material deposition can be achieved through digital design (Blachowicz et al., 2023). Figure 16c focuses on the internal structure of the piezoelectric-pneumatic material inkjet printhead. This on-demand droplet-ejection technology can handle high-viscosity inks and precisely deposit composite inks containing conductive carbon materials at designated locations, enabling the digital manufacturing of functional devices (Blachowicz et al., 2023). Figure 16d shows the optical images of the printed MCF/SR composite and its sandwich-structured sensor (S-MCF/SR) — the image with a scale of 7 mm clearly presents the macroscopic morphology and structural integrity of the device, verifying the feasibility of this ‘FJH processable materials additive manufacturing’ approach (Blachowicz et al., 2023). This series of illustrations collectively demonstrates the deep integration of FJH technology with material processing techniques, opening a new path for the high-value utilization of carbon-containing waste and the integrated manufacturing of functional devices. It offers a universal and efficient platform for processing various solid wastes, such as electronic waste, scrap tires, and agricultural and forestry residues, truly achieving a closed-loop ‘turning waste into treasure’ approach.

**FIGURE 16:**
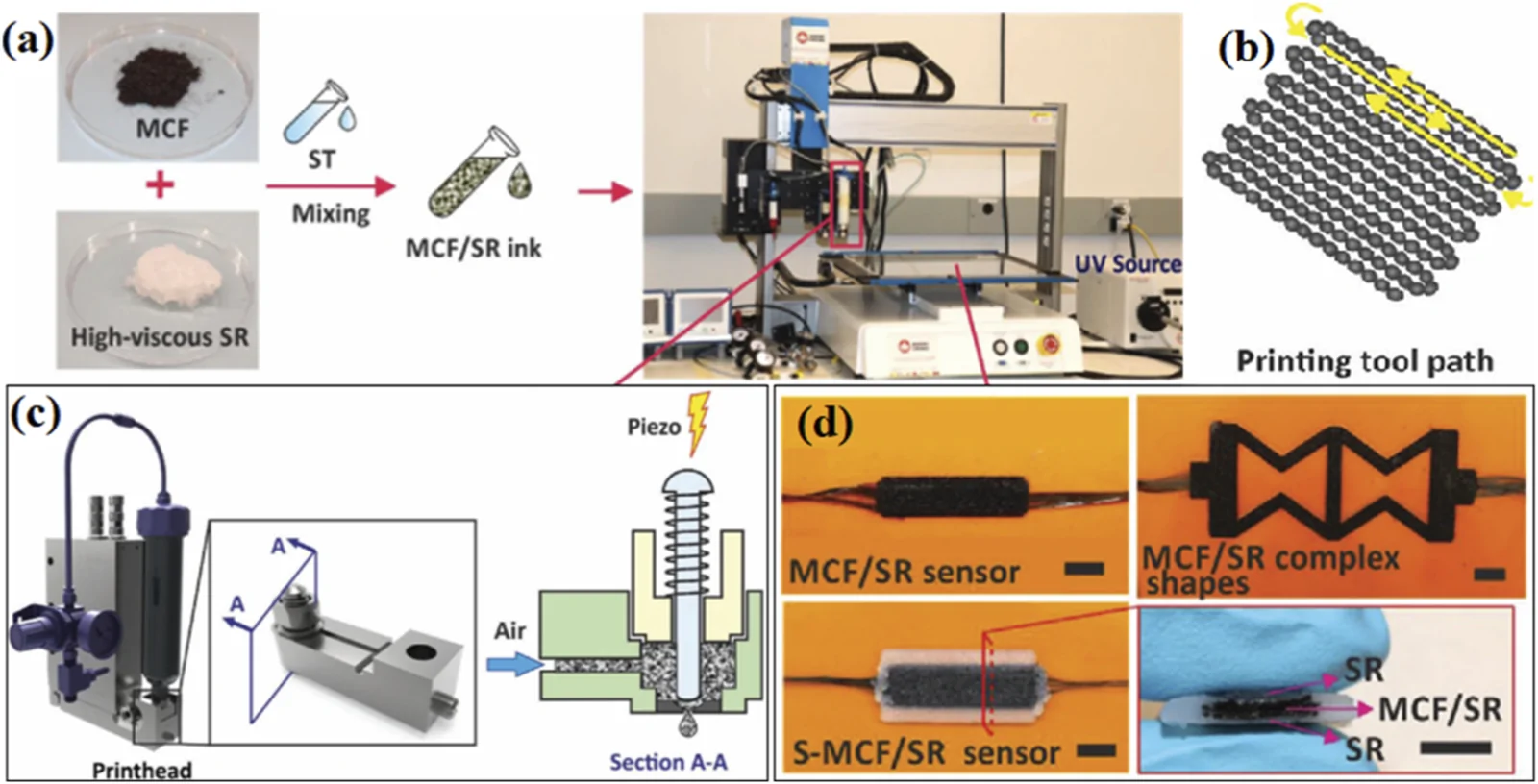
**(a)** Fabrication process of milled carbon fiber/silicone rubber (MCF/SR) composites with silicone thinner (ST) (Blachowicz et al., 2023); **(b)** printing tool path (Blachowicz et al., 2023); **(c)** piezoelectric-pneumatic material jetting printing head enables drop-on-demand jetting the droplets of high viscous ink (cross-section view of the printing head) (Blachowicz et al., 2023); **(d)** optical images of the printed MCF/SR and sandwiched sensors (S-MCF/SR). Scale bars: 7 mm (Blachowicz et al., 2023).

Extreme compression of time and energy consumption: Unlike traditional processes that require hours or even days of prolonged high temperatures, FJH reactions can be completed in milliseconds (Algozeeb et al., 2020). In theory, this speed can increase traditional production efficiency by 5–6 orders of magnitude. At the same time, because the energy is deposited almost directly and entirely into the reactants in the form of electrical energy, thermal loss is minimal. The electric energy cost to produce flash graphene is only ≈7.2 kJ g^-1^ for high-carbon feedstocks (e.g., carbon black, coal) and ≈23 kJ g^-1^ for plastic waste (Algozeeb et al., 2020; Lacerda et al., 2023b), i.e., 2–3 orders of magnitude lower than conventional CVD. Life-cycle assessment further shows that FJH graphene from biomass waste lowers carbon and water footprints by >90% relative to graphite-based graphene (≈10–16 kg CO_2_ per kg graphene versus 400–500 kg CO_2_ per kg for conventional CVD/exfoliation graphene) and is > 10× cheaper (Hosny et al., 2025). These primary data substantiate the claimed energy and CO_2_ advantages of FJH.

##### 4.1.2.2 Extreme compression of time and energy consumption

Unlike traditional processes that require hours or even days of prolonged high temperatures, FJH reactions can be completed in milliseconds (Yuan et al., 2026). In theory, this speed can increase traditional production efficiency by 5–6 orders of magnitude. At the same time, because the energy is deposited almost directly and entirely into the reactants in the form of electrical energy, thermal loss is minimal. This means the energy consumption required to produce each gram of graphene can be 2–3 orders of magnitude lower than that of conventional chemical vapor deposition methods, greatly reducing manufacturing costs (Liu F. et al., 2022; Shi et al., 2022).

##### 4.1.2.3 Potential continuous production

The FJH process is extremely simple—press the precursor into a tablet or place it in the reaction chamber, then apply electricity; it requires no complex airflow control, vacuum environment, or lengthy heating and cooling procedures (Hosny et al., 2025). This simplicity makes large-scale production possible. In theory, as long as the device is designed for drum feeding or continuous discharge, continuous production can be achieved, thereby paving the way for true industrial-scale mass production.

Therefore, FJH is not just a technical method, but a new manufacturing philosophy of ‘replacing heat with electricity and speed overcoming slowness.’ With its extremely simple operation and rapid efficiency, it breaks the constraints of traditional manufacturing on raw materials, time, and energy consumption, ushering material science from ‘precision processing’ into a new era of ‘ultrafast universality.’

### 4.2 Microwave-assisted synthesis: the unique value of selective bulk heating

Unlike direct electric energy deposition with FJH, microwave synthesis utilizes the interaction between a high-frequency electromagnetic field (typically 2.45 GHz) and matter (Table 4). Its revolutionary aspect lies in achieving selective, bulk heating, thereby overcoming the temperature gradient and thermal lag problems of conventional conductive heating (Meloni et al., 2023). Through the dielectric heating effect of the microwave field, energy can be directly deposited in specific dielectric molecules, avoiding heat-transfer losses in solid conductors (Mishra et al., 2024; Wu et al., 2023). This volumetric sensing characteristic enables the reaction system to achieve a uniform and highly efficient thermal activation state in a very short time, effectively increasing the reaction rate and reducing, to a certain extent, the reliance on inert atmospheres and high-temperature furnaces, providing a new physical driving pathway for the rapid preparation of high-performance carbon materials.

**TABLE 4:** The unique advantages of microwave synthesis compared to FJH.

Comparison dimension	FJH (direct energy deposition)	Microwave-assisted synthesis (MAS)
Mode of energy transfer	Electron transfer: Current flows inside the conductor, and kinetic energy is directly converted into heat energy (Cheng et al., 2022). (Requires contact with a good conductive network)	Electromagnetic induction: High-frequency electromagnetic fields penetrate matter, causing dielectric loss within the molecules of the medium (Kömür et al., 2025). (No physical contact needed, highly penetrative)
Heating area	Conductive phase: Only conductive networks such as graphene can be heated, while the insulating phase or matrix is difficult to heat directly (Xia et al., 2024; Zhao et al., 2022)	Dielectric phase: Any material with polar bonds or conductive pathways can be heated (Kömür et al., 2025). (such as water molecules, functional groups, and some conductive polymers)
Temperature Distribution	Limited: High temperature in the conductive network and low temperature in the insulating matrix, with a significant temperature gradient present (Zheng-De et al., 2019)	Uniform/Bulk phase: Energy is deposited inside the molecules, which in theory can achieve more uniform bulk heating (Soltane et al., 2023)
Atmosphere-dependent	High: Usually requires a vacuum or inert gas environment to prevent arcing or oxidation (Wyss et al., 2022)	Medium/Low: In some cases, microwaves can operate in air at atmospheric pressure, but the atmosphere still needs to be controlled for oxidation-sensitive materials (Soltane et al., 2023)
Reaction rate	It depends on the continuity of the conductive network: a discontinuous network will lead to low heating efficiency (Cheng et al., 2022)	Rapid: Dielectric induction heating eliminates the lag of heat conduction, allowing reactions to reach uniform activation in a very short time (Kömür et al., 2025)
Reported energy and CO_2_-footprint values	Energy: −7.2 kJ/g for gram-scale flash graphene synthesis from carbon sources (Algozeeb et al., 2020; Wyss et al., 2023); −23 kJ/g (-$125 electricity/ton of plastic feed) for graphene upcycling from plastic waste (Wyss et al., 2023). CO_2_ footprint: Near-negligible CO_2_ emissions under renewable energy assumptions in cradle-to-gate LCA (Wyss et al., 2023); 39%–84% emission reduction vs. other H_2_ production routes (Silva, 2025)	Energy: In a direct comparative LCA, microwave deconstruction required significantly more energy (4,888.3 MJ) than the FJH route for comparable waste-plastic conversion (Wyss et al., 2023). MAS generally offers energy savings vs. conventional thermal heating by accelerating reactions, though its net sustainability depends on energy input, solvent, and process factors (Luong et al., 2020). CO_2_ footprint: -3.16 kg CO_2_-eq generated per process run in the same comparative LCA study (Algozeeb et al., 2020; Zou et al., 2025)

The unique value of selective volumetric heating is primarily reflected in the revolutionary leap in energy utilization efficiency (Eze et al., 2024). Unlike traditional ‘metal heating,’ where the reactor must be heated first, and heat is conducted layer by layer, microwave fields directly ‘communicate’ with the target molecules, instantly converting electromagnetic energy into molecular kinetic energy—that is, heat (Meloni et al., 2023). Once the graphene network develops cracks, interfacial thermal resistance blocks Joule heat, causing a large amount of heat to dissipate at the graphene-matrix boundary (Shi et al., 2025). Microwaves, however, can penetrate these discontinuities and deliver energy directly to the interior of the substrate, redefining the concept of ‘heat loss’ (Wagih et al., 2022). Since heat is generated only at the reaction site, warming the surrounding environment becomes a side effect, and overall energy consumption is significantly reduced (Zhou et al., 2025).

Beyond efficiency, both the reaction rate and uniformity have also been redefined. The traditional heat conduction mode of ‘from surface to interior’ has an inherent time lag. In contrast, microwaves can bring the entire system to a uniformly thermally activated state within milliseconds, virtually eliminating temperature gradients (He et al., 2025). The temperature rises extremely quickly, and the energy is highly focused, leaving no time for side reactions or thermal decomposition, so the product purity is correspondingly improved. More importantly, the equipment threshold is significantly lowered: Flash Joule heating (FJH) requires high vacuum or a high-purity inert atmosphere to prevent graphene oxidation and stabilize the electric arc, whereas microwave synthesis can operate at normal pressure in systems like hydrothermal setups, requiring only minimal atmosphere control (Deng et al., 2025; Hosny et al., 2025). The penetration and bulk-phase induction characteristics of microwaves also allow it to easily handle complex systems such as solutions and gels containing metal ions—these ‘tough’ targets are often unmanageable in traditional FJH.

Therefore, microwave-assisted synthesis is not an alternative to FJH, but rather provides a parallel and complementary physically driven pathway: FJH excels at ‘point-to-point’ ultra-high temperatures and ultra-rapid heating, suitable for the direct sintering of bulk graphene or graphene-based composites; microwaves, on the other hand, utilize dielectric heating to rapidly, gently, and uniformly activate the entire reaction volume, making them well-suited for scenarios requiring rapid phase transitions, dehydration, or solvent evaporation. One is rigid, the other flexible, and together they push the preparation of high-performance carbon materials to a more flexible and energy-efficient new dimension.

#### 4.2.1 Mechanism: coupling effect with polar substances/catalysts

The essence of microwave heating is the coupling effect: the microwave electric field directly interacts with the microscopic structure inside the material, converting electromagnetic energy into heat (Yoshikawa, 2020). For polar molecules (such as water, alcohol, or groups containing hydroxyl or carboxyl), an alternating electric field causes their dipole moments to orient repeatedly, and molecular rotation and friction generate dielectric loss, leading to heating (Chu et al., 2025). For ions (Fe^3+^, Ni^2+^, etc.) or free electrons, an alternating electric field drives their directional migration, releasing heat due to resistive loss (Fayaz et al., 2024). This process has two significant characteristics: selectivity and instantaneity. Selectivity is reflected in the large differences in the loss factors of different substances, where only strongly absorbing components are heated while weakly absorbing components hardly increase in temperature. Therefore, the energy input area can be precisely targeted by adding a small amount of microwave sensitizer (He et al., 2026). Instantaneous heating is characterized by energy being released synchronously within the material, without the need for conduction from the surface to the interior. The heating rate is much higher than that of conventional convective heating, with minimal heat loss and low energy consumption. In the synthesis of carbon materials, the advantages of microwave heating are demonstrated through the ‘hot spot’ mechanism: microwave sensitizers (such as Fe^3+^, Ni^2+^ salts, or defective carbon) rapidly absorb energy under irradiation, forming localized high-temperature regions whose temperatures are significantly higher than the surrounding uniform temperature (Kumar and Kumar, 2025). These high-temperature zones can induce the graphitization of carbon matrices within seconds, or generate pores under millisecond thermal shocks, directly producing porous carbon with a high specific surface area (Zhang H. et al., 2024). The traditional high-temperature process, which used to take hours or even days, is thus reduced to seconds or minutes, greatly improving efficiency.

#### 4.2.2 Application in the rapid preparation of functional carbon composites

Microwave technology, with its unique rapid and selective heating characteristics, plays a pivotal role in preparing functional carbon composites. First, it enables ultra-fast graphitization: by mixing graphene oxide or amorphous carbon with a microwave sensitizer, the microwave field can efficiently complete the reduction or graphitization process within minutes, producing high-quality graphene or graphitized carbon and greatly shortening the time required for traditional heat treatment (González et al., 2021; Kumar R. et al., 2021). Secondly, microwave technology excels at one-step synthesis of composite materials. In a microwave field, when a carbon precursor (such as glucose or biomass) is mixed with a metal salt solution, the metal ions can be reduced *in situ* to nanoparticles while the carbon precursor undergoes carbonization. This “one-pot” synthesis strategy is not only fast but also uses rapid microwave heating to inhibit excessive nanoparticle aggregation, resulting in metal/carbon composites with uniform size and excellent dispersibility (Dai et al., 2020). Finally, microwaves also perform excellently in functionalization and activation: they can efficiently drive reactions between precursors containing heteroatoms such as nitrogen, sulfur, and phosphorus and the carbon framework, enabling rapid and uniform heteroatom doping (Rangraz and Heravi, 2021; Zhang C. et al., 2025). At the same time, by utilizing the strong interaction between microwaves and activators such as KOH, chemical activation can be completed within tens of minutes, producing activated carbon with a much higher specific surface area, while significantly reducing energy consumption compared to traditional methods.

### 4.3 Bottleneck analysis: the tension between efficiency and precision control

Although flash Joule heating (FJH) and microwave technology offer unparalleled efficiency, as emerging technologies, they face profound challenges on the path from lab-scale achievements to reliable industrial production, with the core issue being how to harness their extreme, rapid processes to achieve predictable, controllable products.

First is the challenge of controlling product structural uniformity. For FJH, the millisecond-level, extreme non-equilibrium process makes the reaction highly sensitive to precursor uniformity, conductivity, particle size, packing density, and fluctuations in current parameters (voltage, pulse width, waveform) (Figures 17a–d; Zhao et al., 2025). This means that to achieve stable production from laboratory to industrial scale, the FJH system must be precisely designed and strictly controlled. Figure 17a presents a detailed schematic of the core components of the FJH device, including a high-voltage power supply, energy storage capacitor bank, high-speed switch, reaction chamber, and data acquisition system (Zhao et al., 2025). The selection and matching of each component’s parameters directly affect the final current-voltage characteristics applied to the sample. Figures 17b,c present, in the form of actual photographs, the overall layout of the FJH system and the details of the reactor respectively—from the scale of the high-voltage capacitor bank to the spacing of the reactive electrodes, from the material of the sample container to the reliability of the electrical connections, these engineering details collectively determine whether it is possible to stably deliver the preset ultra-high current pulses to the sample within millisecond timescales (Zhao et al., 2025). Figure 17d shows a detailed schematic of the temperature control system (CTS), illustrating how precise temperature monitoring and feedback control ensure reproducible, safe reactions during extreme thermal cycling (Zhao et al., 2025). This series of illustrations collectively reveals a core point: the engineering success of FJH technology depends not only on understanding extreme non-equilibrium thermodynamic processes, but also on precise control of every process parameter that could affect the current-heat-matter conversion pathway. Minor disturbances can lead to local ‘overburning’ or ‘underburning,’ causing batch-to-batch variations in the product’s defect types and density, degree of graphitization, number of layers, and even phase composition. Therefore, precisely regulating pulse parameters (such as multi-pulse sequences) to ‘program’ the temperature-time profile and achieve directed synthesis of specific structures (such as specific defect concentrations) represents a major challenge at the fundamental science level. In comparison, the difficulty of microwave synthesis lies in the inherent problem of uneven microwave field distribution (hot spots and cold spots) within the reaction chamber, which easily leads to uneven heating (Mishra et al., 2024). Differences in the dielectric properties of precursors may also cause selective heating, leading to uneven distribution of components in the composite material (Tao et al., 2024; Wang et al., 2023c). Product uniformity depends heavily on reactor design and how materials are mixed.

**FIGURE 17:**
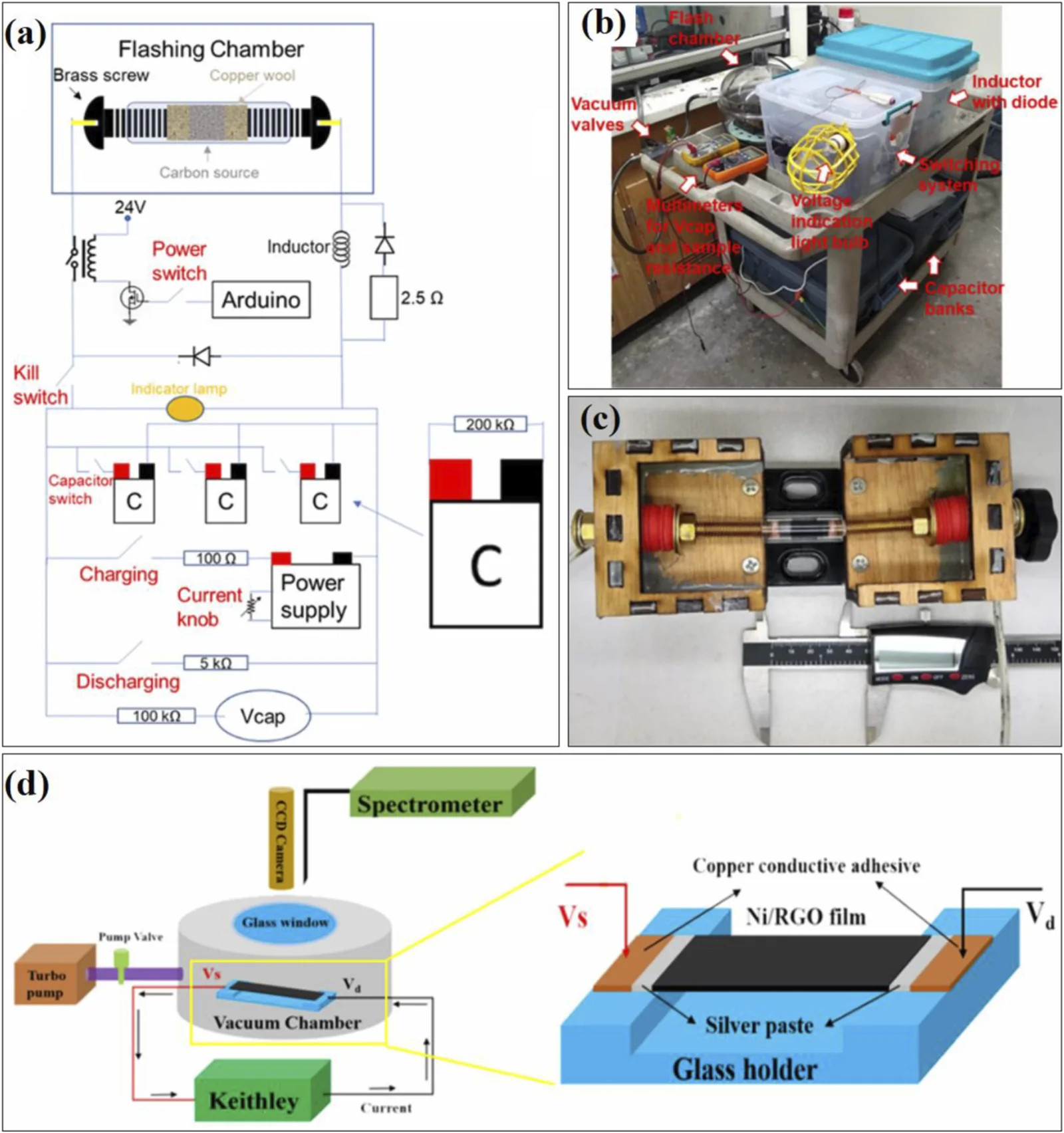
**(a)** Detailed diagram of the FJH setup (Zhao et al., 2025). **(b)** Photo of the FJH setup (Zhao et al., 2025). **(c)** Photo of the FJH reaction device (Zhao et al., 2025). **(d)** Detailed diagram of the CTS setup (Zhao et al., 2025).

Next are the challenges of reactor scale-up design and engineering. Scaling up FJH from laboratory gram-scale to industrial ton-scale requires addressing the stable and safe supply and switching of ultra-high currents (tens of thousands to hundreds of thousands of amperes), as well as achieving continuous, uniform feeding of precursors and collection of products (Deng et al., 2025). At a large scale, ensuring uniform electro-thermal conversion and avoiding unstable phenomena such as arc discharge is a severe engineering challenge. Scaling up microwaves is not simply a matter of increasing size. As scale increases, limited microwave penetration depth (skin effect) becomes more prominent, making it extremely difficult to maintain uniformity in the electromagnetic field within the cavity (Chen Z. et al., 2025). Addressing this requires multi-source, mode-stirring, or flow reactors, which greatly increases system complexity and cost.

Third is the lack of process standardization and mechanistic understanding. These two technologies, especially FJH, have extremely fast reaction processes, making *in situ* characterization very difficult and limiting our understanding of the atomic-scale dynamic evolution mechanisms occurring inside the ‘black box’ (Yuan et al., 2023). Accurate temperature measurement is a challenge in itself. The lack of a quantitative understanding of the ‘process parameters-intermediate processes-final structure’ makes process development heavily reliant on trial and error, making it difficult to establish reliable process control standards and quality prediction models, and hindering its application in high-end fields that require strict consistency (such as the semiconductor industry).

Therefore, flash Joule heating combined with microwave synthesis represents a revolutionary manufacturing philosophy: exchanging extreme energy efficiency and time compression for tremendous potential in sustainability and cost-effectiveness (Figures 18a–d; Deng et al., 2025; Hu et al., 2023). First, this logic of ‘trading time for performance’ has been intuitively validated at the material-structure level—Raman spectroscopy analysis in Figure 18a demonstrates the FJH process parameters’ ability to fine-tune the structure of flash graphene. By adjusting the pulse voltage and duration, an optimal balance between defect density and conductivity can be achieved (Hu et al., 2023). Supporting this precise control is a meticulously designed hardware system: Figures 18b,c reveal the engineering implementation of the FJH device from the perspectives of overall layout and local details—ranging from the energy storage of the high-voltage capacitor bank to the spacing adjustment of the reactive electrodes; every aspect ensures the reproducibility of reactions in extreme non-equilibrium states (Hu et al., 2023). Extending this perspective to microwave synthesis, the same philosophy appears in a different form—the schematic of the continuous-flow microwave reactor in Figure 18d shows how rapid, molecular-level heating can be achieved in a flow system. This design perfectly combines energy efficiency with production continuity, providing a new technical approach for the large-scale preparation of functional carbon materials (Hu et al., 2023). These illustrations make clear that, whether through FJH’s pulsed current or microwave dielectric heating, the common core breaks the path dependence of traditional thermodynamics through an extreme rate of energy input, ultimately realizing the promising vision of sustainable manufacturing. They are not just tools for ‘faster and more efficient’ processes, but keys to unlocking new states of carbon materials (non-equilibrium structures) and new chemistry. However, the success of this ‘process revolution’ depends not only on innovations in engineering devices but more urgently on a deep understanding of physicochemical processes under extreme non-equilibrium conditions, so that this ‘lightning-like’ miracle can be tamed into a precise, reliable, and predictable manufacturing science. Their future lies in the deep integration with advanced *in situ* characterization, multi-scale computational simulations, and intelligent control systems, thereby truly achieving the leap from ‘astonishing phenomena’ to ‘trustworthy processes’.

**FIGURE 18:**
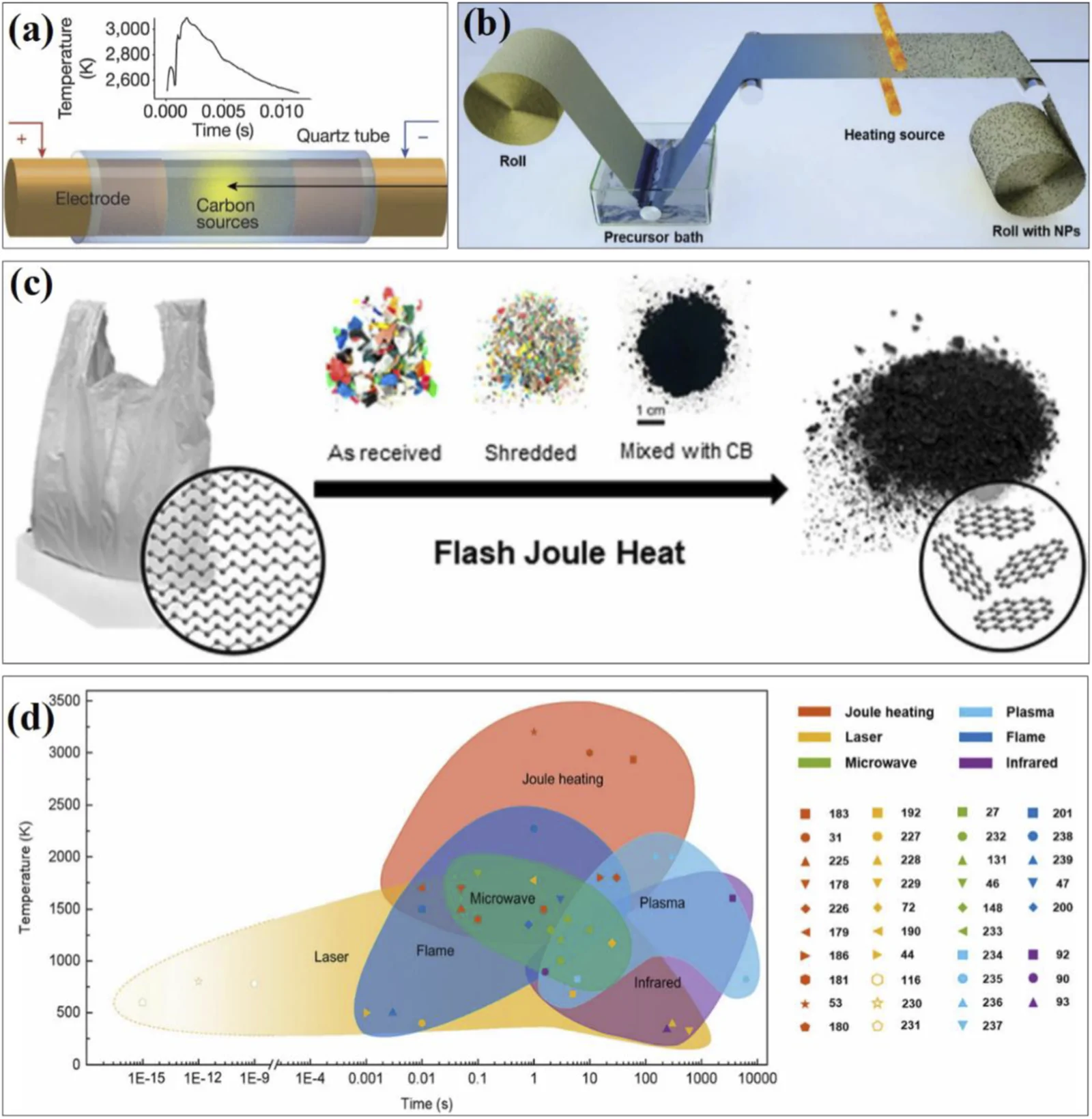
**(a)** Schematic of the flash Joule heating process and its temperature vs. time plot (inset) (Hu et al., 2023). **(b)** Schematic of roll-to-roll production of nanoparticles loaded on an arbitrary substrate (Hu et al., 2023). **(c)** Schematic of turning a plastic bag into graphene powder through flash Joule heating (Hu et al., 2023). **(d)** Diagram of synthesis conditions of ultrafast methods: temperature vs. reaction time (Hu et al., 2023).

A balanced assessment is therefore essential: the extreme energy/time efficiency of FJH and microwave synthesis is purchased at the cost of reduced deterministic control over atomic-scale structure. FJH can reach >3000 K in ∼100 m and quench at ∼10^4^ K s^-1^ (Luong et al., 2020), which is ideal for bulk turbostratic graphene but makes deliberate placement of individual dopants or defects difficult—defect density is governed by average voltage/pulse rather than by molecular design (Liu and Fan, 2023). Microwave volumetric heating gives superior field uniformity and avoids arc instabilities but is limited to lower peak temperatures and to polar/conductive matrices (Chen Z. et al., 2025; Deng et al., 2025). By contrast, slow furnace pyrolysis or bottom-up synthesis sacrifice speed yet retain far greater control over elemental composition and edge/defect structure. The pragmatic conclusion is that rapid physical methods are best positioned for high-volume, defect-tolerant applications (e.g., conductive additives, sorbents, composite fillers). In contrast, precision-critical applications (quantum devices, single-atom catalysts) still favor template or bottom-up routes. Future “process programming”—closed-loop control of pulse shape, atmosphere, and precursor formulation—is the route to narrowing this gap (Liu and Fan, 2023).

## 5 Molecular precision manufacturing: bottom-up synthesis for ultimate control of electronic structure

When we shift our perspective from the macroscopic, statistical view to the ultimate pursuit of the properties of individual carbon nanostructures, a completely different philosophy and technical approach takes center stage—this is ‘bottom-up’ organic synthesis. On this path, we no longer rely on thermodynamically driven, statistical, or overall cracking and recombination of precursors (such as pyrolysis), nor do we use external templates for morphology replication. Instead, we return to the essence of chemical synthesis. Through a series of atom-economical covalent bond reactions conducted in solution or on surfaces, we gradually and precisely ‘build’ target carbon nanostructures from structurally well-defined organic small-molecule precursors (Islam and Ghosh, 2025). The core of this method lies in atomic-level precision control—we no longer carbonize a piece of stone at high temperatures, but rather, like building with blocks, connect atoms to construct finely structured materials such as nanoscale graphene, carbon nanoribbons, and helical nanocarbon tube fragments. If the template method is ‘carving’ and new physical technologies (such as FJH and microwaves) are ‘quenching,’ then bottom-up synthesis is ‘molecular architecture.’ Its goal is to achieve complete control over every structural parameter of carbon materials with atomic-level precision (Li J. et al., 2023). This level of control allows us to achieve theoretically predictable and experimentally verifiable deterministic performance—not just average values, but precise manipulation of the quantum behavior of individual nanoscale entities. This transformation is not merely a technological evolution, but a cognitive leap: we have moved from focusing on the average properties of materials to understanding and controlling the behavior of individual nanoscale and even molecular entities, opening a new chapter in carbon materials science.

### 5.1 The essence of methodology: the ‘art of synthesis’ from identifying molecules to determining structures

This ‘bottom-up’ synthetic methodology essentially lies in perfectly integrating the ‘art’ of chemical synthesis with the ‘engineering’ of materials science. It no longer relies on the thermodynamic driving forces of chaotic systems, but returns to the fundamentals of chemistry—well-defined molecular structures and precise bond control (Alzeer, 2024). Specifically, it begins with an organic molecule whose structure and purity are completely known (typically polycyclic aromatic hydrocarbons or their derivatives). Through carefully designed synthetic routes (such as the Diels-Alder reaction, cyclodehydrogenation, Scholl reaction, or transition metal-catalyzed coupling), chemists can, much like building with LEGO bricks, assemble molecular modules according to a preset blueprint, gradually ‘growing’ nanostructures with a defined atomic arrangement by forming specific C-C bonds (Barrett, 2024; Hassan et al., 2020). This synthesis can be carried out both in solution and on the surface of a metal single crystal (such as gold, silver, or copper). The latter is more like a ‘molecular alchemy furnace,’ where cyclization and dehydrogenation are completed through surface catalysis under ultra-high vacuum and precisely controlled temperature conditions. This methodology provides unparalleled structural precision in material preparation, making it a unique bridge connecting organic chemistry, materials science, and condensed matter physics.

### 5.2 Unmatched structural precision: the causal chain of atomic-level engineering and quantum properties

At the forefront of contemporary materials science, we are experiencing a revolutionary shift from ‘macroscopic control’ to ‘atomic-level programming.’ Atomic-level control, once seemingly out of reach, has now become a rapidly maturing technology, enabling us to establish the most direct and intrinsic structure–property relationships and achieve ‘ultimate control’ over electronic structures. This precision is most evident in three areas: the design of graphene nanoribbons (GNRs), the manipulation of doping sites, and the introduction of complex topological structures.

First, in the synthesis of graphene nanoribbons, traditional ‘top-down’ methods often struggle to control precise atomic arrangements, whereas bottom-up chemical synthesis techniques offer a revolutionary solution (Palagati et al., 2024; Palagati and Reddy, 2024). Research has found that the band gap of nanoribbons is not arbitrary, but is precisely determined by their width—that is, the number of carbon atom rows (N) (Niu et al., 2022). This band gap exhibits a peculiar ‘one-third oscillation rule’ (3p, 3p + 1, 3p + 2 series), where changing the width by just one or two carbon atoms can turn a semiconductor into a metal, or *vice versa* (Zito, 2023). Furthermore, the edge shape of the nanoribbon (armchair or zigzag) determines its fundamental properties: armchair nanoribbons usually behave as semiconductors and are particularly ideal when seven carbon atoms wide (Talkachov and Babaev, 2023). The zigzag shape ideally exhibits metallic properties, but it may lead to spin polarization due to edge-localized states, displaying unique magnetic characteristics (Zhu and Wang, 2025). By carefully designing organic precursor molecules, scientists can now induce nanoribbons of specific widths and edge shapes on metal surfaces. This means we no longer unthinkingly remove excess carbon atoms, but instead ‘write’ the desired band structure at the atomic level. Furthermore, in controlling doping sites, this precision is more like a ‘microscopic scalpel.’ Traditional doping methods (such as thermal treatment or ion implantation) can only control the doping concentration, but cannot control the exact positions of individual atoms (Sethuraman and Liu, 2024). Now, by pre-embedding specific heteroatoms (such as nitrogen, boron, and sulfur) into organic precursors and designing their relative positions within the molecule, scientists can control the location, chemical state, and spatial arrangement of these heteroatoms in the final nanostructure (Mahmood et al., 2025). For example, precisely placing nitrogen atoms at the edges of graphene, rather than at the center, can greatly alter its local charge distribution and significantly enhance the material’s Lewis basicity, thereby optimizing its performance in electrocatalytic reactions (such as the oxygen reduction reaction, ORR) (Figures 19a–c; Wu et al., 2022). The microscopic origin of this edge-doping effect can be revealed through atomic-scale characterization techniques. The scanning tunneling microscope (STM) topography image in Figure 19a shows the atomic-level configuration of pyridinic nitrogen defects—nitrogen atoms located at the edges or vacancy edges of graphene, bonded to two carbon atoms (Wu et al., 2022). The simulated STM image in the figure (bias −0.1 V) closely matches experimental observations, while the scanning tunneling spectroscopy (STS) on the right shows the unique local electronic density of states at this site, providing electronic-structure evidence for understanding its catalytic activity (Wu et al., 2022). In contrast, Figure 19b shows a graphite-type nitrogen defect—in which a nitrogen atom replaces a carbon atom in the hexagonal ring of graphene, located within the lattice (Wu et al., 2022). Simulated STM images (bias 0.5 V) and experimental images together reveal its electron cloud distribution characteristics, which differ from pyridinic nitrogen. At the same time, STS spectra further confirm fundamental differences in the electronic structure between the two nitrogen species (Wu et al., 2022). Figure 19c systematically summarizes the common nitrogen species and defect configurations in carbon materials in a schematic form: 1) Pyridinic nitrogen (Pyr-N3) adjacent to a single vacancy, corresponding to the atomic structure in Figure 19a; 2) Porphyrin-type nitrogen (Por-N4) adjacent to a double vacancy, with nitrogen atoms existing in a porphyrin-like coordination environment; 3) Pyrrolic nitrogen (NPY) associated with Stone–Thrower–Wales defects, where nitrogen atoms participate in the formation of five-membered rings (Wu et al., 2022). Among these three configurations, the pyridinic nitrogen located at the edges or around vacancies often exhibits the best ORR catalytic activity due to its unique electron-donating properties. This is a typical example of how ‘site engineering’ surpasses ‘content engineering’.

**FIGURE 19:**
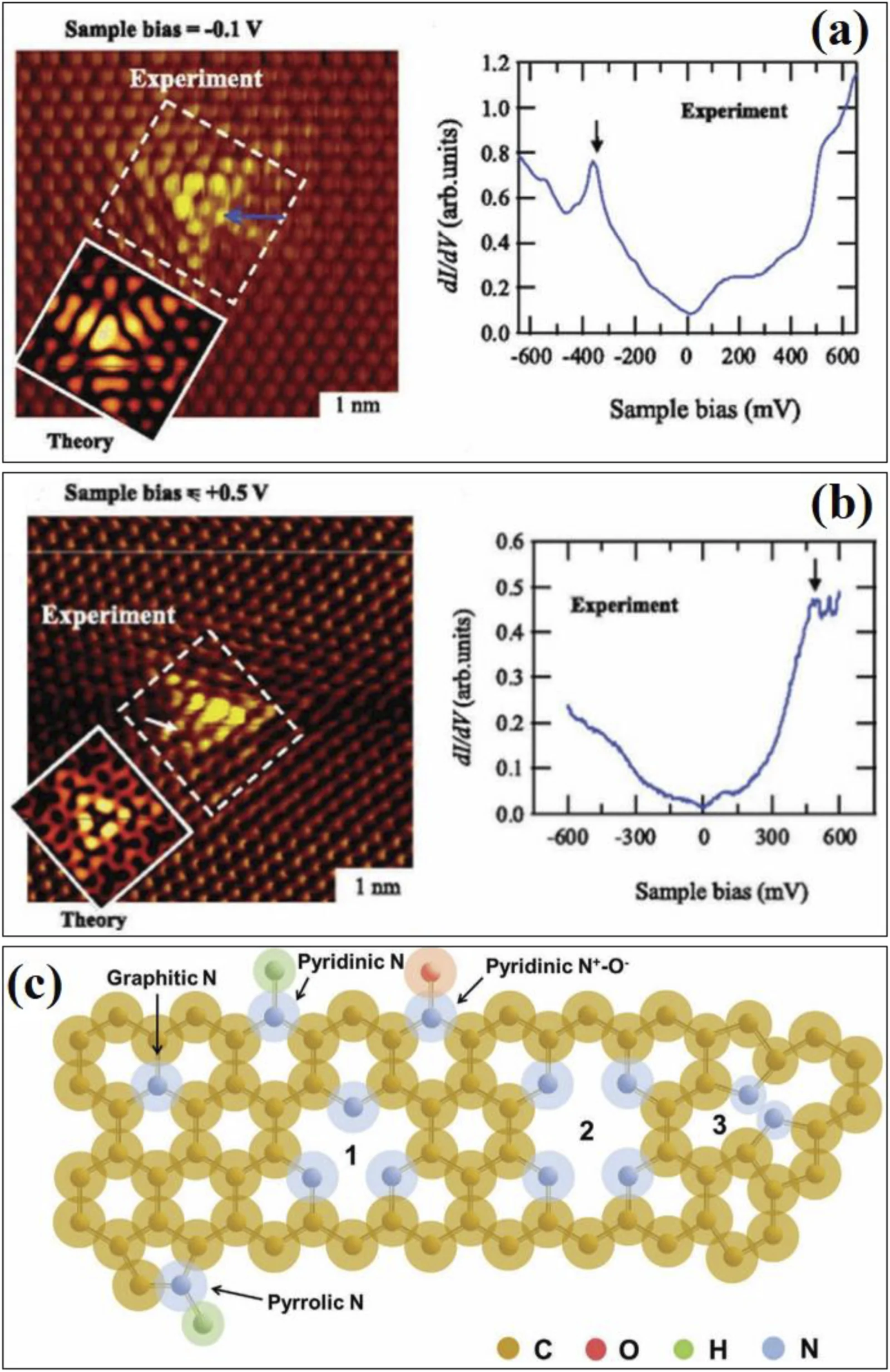
**(a)** STM topographic image of a pyridinic N defect. The simulated STM image (V = −0.1 V) is also shown for comparison. STS spectrum was measured at the position indicated by the arrow on the left side in **(a)** (Wu et al., 2022). **(b)** STM topographic image corresponding to graphitic N defects. The simulated STM image (V = +0.5 V) is also shown for comparison. STS spectrum was measured at the position indicated by the arrow on the left side in **(b)** (Wu et al., 2022). **(c)** Schematic illustration of common N species and defects in carbon materials. 1) Pyridinic N with monovacancy (Pyr-N3), 2) porphyrinic N divacancy (Por-N4), and 3) pyrrolic N (NPY) with Stone–Thrower–Wales defect (Wu et al., 2022).

Such performance improvements cannot be achieved through conventional random doping methods, reflecting a leap from ‘rough processing’ to ‘precise tuning.’ Finally, in exploring topological structures, atomic-level engineering breaks the limitations of the perfect hexagonal honeycomb lattice of traditional graphene (Abbas et al., 2025). By designing organic precursors that contain five-membered, seven-membered, or even eight-membered rings, scientists can introduce these ‘defects’ into nanocarbon structures. These non-hexagonal rings disrupt the original planar structure, introducing curvature (such as in fullerene fragments) or forming special topological edge states (Sabirov et al., 2024). Since these structures are often in thermodynamically non-equilibrium states and cannot be obtained through conventional methods such as high-temperature treatment or exfoliation, they may exhibit special electronic correlation effects (such as Mott insulator behavior) or topological properties, representing the forefront of exploring entirely new carbon allotropes in materials science.

Therefore, the precision of this ‘atomic-level engineering’ is transforming ‘materials’ from natural ‘existences’ into ‘artificial crystals.’ It allows us to discover material properties not passively, but to actively ‘write code’ to program their electronic structure. Whether designing a semiconductor with a specific bandgap, creating a material with particular magnetic properties, or exploring entirely new carbon-based states, this technology offers unprecedented possibilities.

### 5.3 Application scenario: demonstrating irreplaceability in areas where ‘precision’ surpasses ‘scale’

At the microscopic scale, the rules of the game in materials science have fundamentally changed: performance is no longer determined by the accumulation of ‘quantity’ but by the precision of ‘quality.’ Carbon nanostructures synthesized from the bottom up, with their atomic-level accuracy, demonstrate core values that macroscopic materials cannot match (Shi et al., 2023). This atomic-level precision yields distinct quantum properties, making these nanostructures irreplaceable core materials in cutting-edge fields such as high-performance molecular electronic devices, quantum information and spintronics, and single-molecule sensing and catalysis.

First, in high-performance molecular electronic devices, bottom-up synthesized nanographene or nanoribbons, due to their structural precision, offer ideal characteristics for constructing molecular transistors (Zhang J. et al., 2026). The edges of these materials can be designed to be perfectly jagged or armchair-shaped, without any defects. This atomically smooth edge can minimize carrier scattering, approaching the theoretical mobility limit and surpassing the silicon-based limit (Gupta and Pham, 2024). In addition, their band structures can be controlled at the atomic level by precisely adjusting the width and topology, providing high reproducibility and predictability (Narang et al., 2021). Macroscopic graphene sheets cannot provide this certainty, because the defects in macroscopic materials are random and uncontrollable, resulting in unpredictable device performance (Sun et al., 2020). Second, in quantum information and spintronics, these nanostructures, through precise edge or defect design, have become ideal platforms for hosting localized electron spin states (Ding et al., 2025). For example, zigzag graphene nanoribbons (ZGNRs) have theoretically predicted spin-polarized edge states, which can be used as solid-state spin qubits (Farghadan, 2024). By artificially embedding specific defects or dopant atoms (such as nitrogen-vacancy complexes), one can construct one-dimensional spin chains, providing a possible route for topological quantum computing (Keçeci, 2025). Atomic-level precision ensures that the coupling strength between spin qubits is uniform, which is crucial for achieving high-fidelity quantum gate operations (Evered et al., 2023). Finally, in single-molecule sensing and catalysis, this atomic-level precision transforms the ‘quantitative’ advantage of macroscopic materials into a ‘qualitative’ advantage at the molecular level. A structurally precise nanographene molecule itself can serve as an ultra-sensitive sensing unit, whose electronic energy levels are extremely sensitive to minute changes in the surrounding chemical environment (such as the adsorption of a single molecule), and it can even be used for single-molecule spectroscopy and DNA sequencing (Figures 20a–d; Ramoso et al., 2025). The physical basis of this ultra-sensitive sensing lies in the strong response of graphene’s electronic structure to external disturbances. Figure 20a illustrates this principle with the shift of the Dirac point in a graphene transistor: when molecules are adsorbed on the graphene surface, its Fermi level shifts, manifested as a displacement of the Dirac point in the transistor’s transport characteristics—this change in the electrical signal can be directly used for quantitative detection of molecular adsorption events (Ramoso et al., 2025). Extending this principle to the field of optical detection, graphene-based surface-enhanced Raman scattering (SERS) platforms demonstrate their unique signal amplification capabilities. Figure 20b illustrates the construction and detection process of a graphene-based signal-enhanced SERS biosensor: (i) the preparation process depicts the engineered modification steps of the graphene substrate; (ii) the working principle of label-free SERS detection reveals how graphene amplifies the Raman signals of adsorbed molecules through a dual mechanism of charge transfer and electromagnetic field enhancement, enabling highly sensitive detection of trace biomolecules (Ramoso et al., 2025). Figure 20c presents the complete workflow of a graphene-based biosensor as a flowchart: from sample pretreatment, surface functionalization, biomolecule probe immobilization, non-specific site blocking, and washing to final detection—optimizing each step directly affects the sensor’s sensitivity and selectivity (Ramoso et al., 2025). Figure 20d vividly illustrates the key molecular events in this process through a molecular-level schematic: (I) covalent modification of the graphene surface by PBSE molecules, introducing functional anchoring points; (II) covalent immobilization of antibodies, endowing the sensor with specific recognition ability; (III) blocking of unreacted sites by ethanolamine, minimizing interference from nonspecific adsorption; (IV) specific biorecognition of the target molecule MMP-9, triggering detectable electrical or optical signals (Ramoso et al., 2025). This cross-scale depiction, from macroscopic devices to molecular events, explains how structurally precise nanographene serves as a core sensing unit, converting molecular-scale chemical disturbances into measurable physical signals and laying the material foundation for precise detection at the single-molecule level.

**FIGURE 20:**
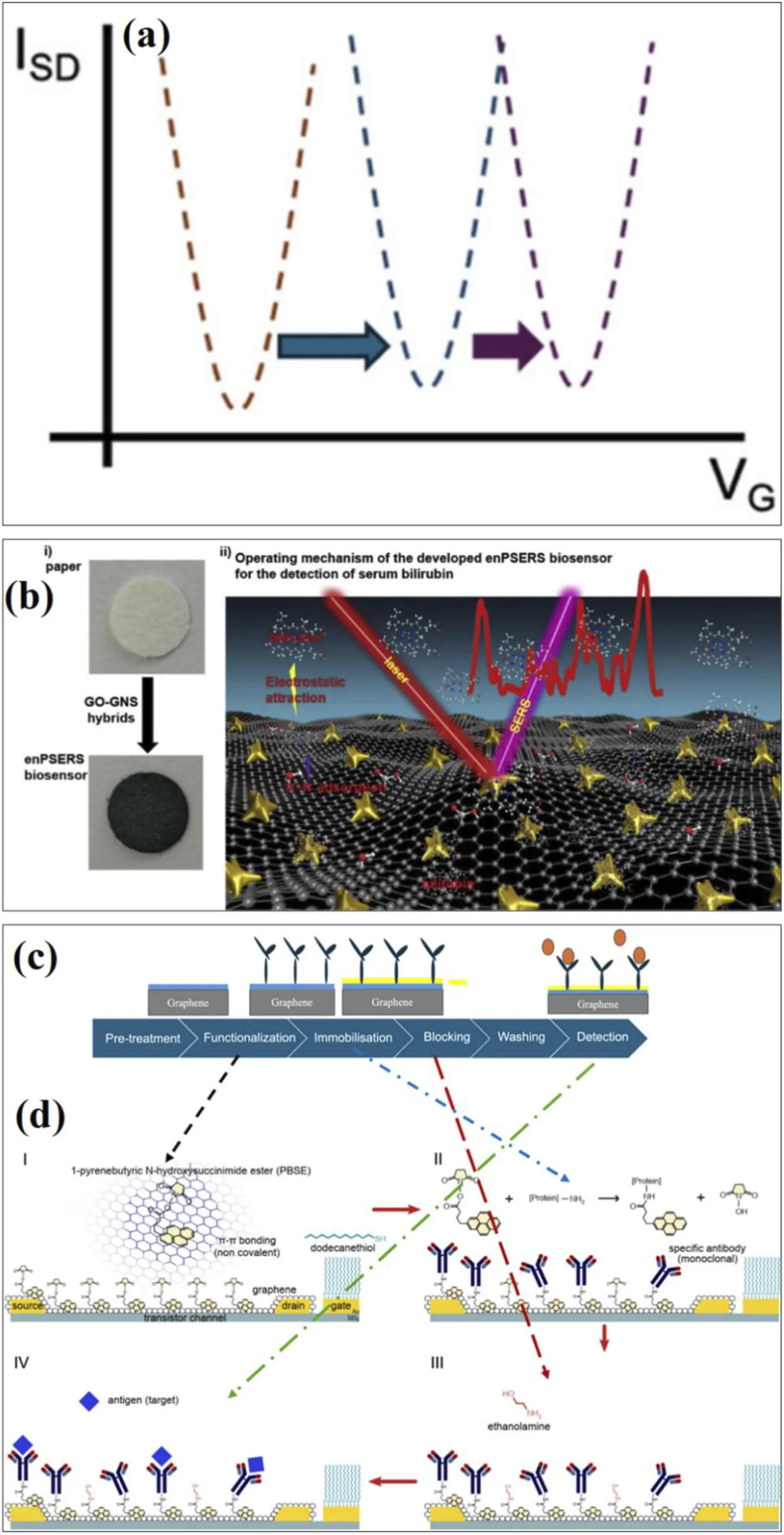
**(a)** Graphene transistors’ sample Dirac point shifts (Ramoso et al., 2025). **(b)** SERS detection platform using graphene-assisted signal enhancement: (i) preparation process of the enPSERS biosensor, and (ii) operating principle for label-free SERS detection. Overview of the biofunctionalisation process for graphene-based biosensors (Ramoso et al., 2025). **(c)** Schematic workflow showing key steps: pre-treatment, functionalisation, immobilisation, blocking, washing, and detection (designed by the authors) (Ramoso et al., 2025). **(d)** Pictorial representation of representative molecular interactions at each step: (I) Surface modification with PBSE, (II) covalent antibody immobilization, (III) blocking with ethanolamine, and (IV) biorecognition of the MMP-9 target (Ramoso et al., 2025).

On the other hand, molecules with well-defined active centers (such as specific edge carboxyl groups or precisely doped nitrogen atoms) can serve as model catalysts to study catalytic reaction mechanisms at the molecular level, without being affected by macroscopic heterogeneity (Jing et al., 2022; Li X. et al., 2025; Vogt and Weckhuysen, 2022). The determinacy of this structure allows precise control of reaction pathways and reactivity at the molecular level.

Therefore, in these fields, performance depends on the ‘quality’ of the structure. Carbon nanostructures synthesized from the bottom up, with their atomic-level precision, exhibit unique quantum effects, establishing their irreplaceable core position in cutting-edge technology.

### 5.4 The scale gap: the intrinsic tension between synthetic art and chemical production

Concretely, bottom-up molecular synthesis achieves angstrom-level edge precision (e.g., atomically defined graphene nanoribbons) but at material yields of only 10%–30% and through >5 unit operations, whereas flash Joule heating and biomass routes reach 60%–90% and 30%–60% yield, respectively, in 1–3 steps (Crista D. et al., 2020; Zhang P. et al., 2026; Zhuo and Levendis, 2014); this yield/step gap-not a vague ‘scale gap’-is the quantitative core of the tension in this section.

In the halls of science, the bottom-up synthetic approach has captivated countless researchers with its unparalleled elegance and power. This method is like a meticulous sculptor, striving to construct perfect structures at the atomic level. However, it is precisely this pursuit of extreme precision—the ‘perfectionism’—that forges its inherent ‘Achilles’ heel’: the scalability gap.

#### 5.4.1 The root of extreme complexity

To achieve atomic-level precision, the synthetic route often requires a long and winding journey. To obtain an ideal molecule, we may need to go through dozens or even hundreds of organic reactions. Each step is like a precise surgery, involving tedious procedures such as separation and purification. As the number of steps increases, the overall yield does not decrease linearly but decays exponentially—the total yield is often less than 1% (Na et al., 2026; Pyle et al., 2026; Song et al., 2021). This means that although we have successfully synthesized the target molecule in the laboratory, the final product may only be produced in milligram or even microgram quantities. Such a tiny yield not only greatly limits the scope of the research but also results in high costs, making commercial applications difficult.

These synthetic-art virtues carry hard industrial penalties that must be stated quantitatively. (i) Multi-step synthesis: preparing a well-defined graphene nanoribbon can require dozens of protections, coupling, and cyclodehydrogenation steps, so even at 80%–90% per-step yield the cumulative yield collapses below 1% (Na et al., 2026; Pyle et al., 2026; Song et al., 2021). (ii) High cost: on-surface bottom-up growth is performed under ultrahigh vacuum (<10^–9^ mbar) on noble-metal substrates (e.g., Au), making it capital- and energy-intensive and limiting throughput to milligram–microgram amounts; nickel-foil CVD variants lower substrate cost but still require controlled annealing and sonication-based exfoliation (Narita et al., 2019). (iii) Solvent/reagent consumption: each protection/deprotection and purification step consumes stoichiometric reagents and organic solvents (e.g., chlorobenzene, toluene, THF), and large nanoribbons suffer poor solubility that forces repeated chromatography (Gu et al., 2022). (iv) Industrial scalability: whereas CVD or FJH can produce grams–kilograms per run, bottom-up molecular synthesis is confined to milligram–microgram lab scale, and transferring perfect molecules into macroscopic films without losing quantum properties remains unsolved (Chaikittisilp et al., 2022; Chen H. et al., 2025). Consequently, bottom-up synthesis is presently a “probe and design guide” rather than a volume-production route.

#### 5.4.2 The ultimate challenge of separation and characterization

Even if we overcome the difficulties of multi-step reactions, we then face the dual barriers of separation and characterization. Many target molecules (especially larger nanoribbons) have very poor solubility, making traditional purification methods difficult to apply (Gu et al., 2022). For structural identification, we often rely on advanced techniques such as single-crystal X-ray diffraction (Ali et al., 2022; Wang N. et al., 2025). However, for these structurally similar and large molecules, growing single crystals large enough for precise measurement is itself a daunting task.

#### 5.4.3 From molecules to materials

What is even more challenging is the process of transforming perfect molecules into macroscopic materials (Chaikittisilp et al., 2022). Even if we hold perfect molecular structures in our hands, how can we assemble these molecules on a large scale and in an ordered manner into usable films, fibers, or bulk materials? This is a major engineering challenge. In disordered aggregated states, the original excellent properties of the molecules often mysteriously disappear, unable to realize the value they are supposed to have.

#### 5.4.4 Convergent strategy exploration

To bridge this gap, researchers are exploring two main approaches. The first is to innovate the synthesis methods themselves, aiming to develop more efficient, fewer-step ‘domino’ reactions or post-polymerization cyclization strategies, thereby shortening the ‘manufacturing time.’ The second is to explore strategic integration with bulk methods, not as a simple replacement but as a clever grafting of advantages. For example, we can use perfectly synthesized nanoscale structures from bottom-up approaches as ‘seeds’ or ‘templates’ to guide the epitaxial growth of large-area, high-quality films in subsequent CVD growth (Etafa et al., 2025; Wang and Liu, 2022). Alternatively, we can design specific, solution-processable polymer precursors that are easy to process into films or fibers on a macroscopic scale, and then ‘stitch’ them into extended graphene networks in the solid state through a single precise ‘ring-closing’ reaction (Chong et al., 2025). The core idea of these ‘convergent’ strategies is that it is no longer simply about replacement, but about combining top-down processability with bottom-up controllability, thereby bridging the gap between ‘molecular marvels’ and ‘practical materials.’ This may well be the cornerstone of future breakthroughs in materials science.

Bottom-up synthesis represents the pinnacle of human control over materials and is a key tool for moving carbon materials from ‘discovery’ to ‘design’ (Palagati and Reddy, 2024; Tripathy et al., 2023). It provides us with the ultimate model system for understanding the intrinsic physicochemical properties of carbon-based nanomaterials. It will play a decisive role in next-generation quantum technologies that have ‘zero tolerance’ for structural defects (Boretti, 2025). However, the nature of its ‘synthetic art’ inherently creates a tension with large-scale, low-cost production that is difficult to reconcile (Chen H. et al., 2025; Zhou and Haq, 2025). To bridge this gap, researchers are exploring two main approaches: innovating the synthetic methods themselves, such as developing more efficient, fewer-step ‘domino’ reactions or post-polymerization cyclization strategies (Highmoore et al., 2024). Second is to explore the strategic integration with macroscopic methods. For example, perfectly synthesized nanostructures created via a bottom-up approach can be used as ‘seeds’ or ‘templates’ to guide the epitaxial growth of large-area, high-quality films in subsequent CVD processes (Wang and Liu, 2022; Xue et al., 2024). Alternatively, design specific, solution-processable polymer precursors that are easy to process into films or fibers on a macroscopic scale, and then ‘stitch’ them into extended graphene networks in the solid state through a precise one-step ‘ring-closing’ reaction (Chong et al., 2025). The core idea of these ‘convergent’ strategies lies in combining top-down processability with bottom-up controllability, rather than simply replacing one with the other, thereby bridging the gap between ‘molecular marvels’ and ‘practical materials’ (Cook and Clemons, 2022; Palagati et al., 2024; Palagati and Reddy, 2024). This may be the cornerstone of future breakthroughs in materials science. Therefore, the future of bottom-up synthesis is not about replacing bulk synthesis, but about serving as a ‘probe’ and ‘design guide.’ On one hand, it is used to explore and verify new physical phenomena and device principles; on the other, the precise ‘structure–performance’ relationships it reveals will, in turn, guide bulk methods (such as improved CVD or FJH) to approach performance limits, thereby finding a future balance between ‘absolute precision’ and ‘practical feasibility.’

## 6 Strategy comparison and integration: towards next-generation integrated intelligent manufacturing

In the previous chapters, we have thoroughly analyzed the intrinsic logic, unique advantages, and inherent bottlenecks of the four major emerging synthesis strategies. They seem like four parallel tracks leading to the common goal of ‘sustainable, high-performance carbon materials,’ each shining on its own, yet isolated from one another. However, true breakthroughs often occur at the intersections of different thinking paradigms and technologies. The core task of this chapter is to go beyond isolated evaluations of individual technologies and instead establish a systematic comparative framework along with an active integration roadmap. We will first visualize the positioning of the four strategies using a multidimensional coordinate system to clarify their respective ‘ecological niches.’ Next, starting from the demands of end applications, we will draw up a ‘map-guided’ strategy selection guide. Finally, and most forward-looking, we will boldly conceive and argue for how proactive and creative cross-integration of strategies can give rise to a next-generation integrated intelligent manufacturing paradigm that surpasses existing paths, thereby transforming the green synthesis of carbon materials from a ‘choose one’ approach to a ‘one plus one is greater than two’ model of collaborative innovation.

### 6.1 Four-quadrant analysis chart: strategic positioning under the tension of ‘controllable precision’ and ‘sustainable scalability’

To more intuitively understand and compare the strategic positions of these four major strategies, we can place them on a two-dimensional, four-quadrant analysis chart with ‘controllable structural precision’ as the vertical axis and ‘sustainability and scalability potential’ as the horizontal axis. This approach not only reveals a pair of core and often competing objectives in modern materials manufacturing—the pursuit of performance limits (requiring precise structures) versus compromises for industrial feasibility (requiring cost and environmental viability)—but also provides clear directional guidance for future innovation paths.

First, let’s look at the ideal ‘holy grail’ area located in the first quadrant (high precision, high sustainability/expandability). Currently, no single strategy fully occupies this quadrant. Emerging physical technologies (such as FJH), due to their broad material applicability (capable of handling waste), ultra-high energy efficiency, and potential for continuous operation, score very high on the horizontal axis (sustainability and scalability) (Deng et al., 2025; Qin et al., 2025). However, its longitudinal position (structural precision) is currently centered because, while it enables rapid transformation of macroscopic products, precise control of atomic-level defects is still in the early stages of exploration, making this a direction that urgently needs improvement in the future through ‘process programming’ (Liu and Fan, 2023). The tension captured by these two axes is precisely the one highlighted in recent evaluations of green synthesis, where energy efficiency, quantified green metrics, and demonstrated scalability are the criteria that decide whether a laboratory route can be considered sustainable at all (Yadav et al., 2026).

In contrast, the representative of the second quadrant (high precision, low sustainability/scale-up potential) is bottom-up organic synthesis. This strategy is undoubtedly the ruler of this quadrant, with structural precision reaching the atomic level, at the top of the vertical axis (Antinucci et al., 2022). However, its complex multi-step synthesis, extremely low yield, and high cost firmly keep it at the left end of the horizontal axis (Jiao et al., 2021). It represents the pinnacle of scientific exploration, but it is the farthest from large-scale industrial application.

In the third quadrant (low precision, low sustainability/scale-up potential), traditional fossil-based synthesis methods and templating strategies in emerging approaches (especially hard templates and complex MOF-derived methods) occupy an awkward position. Although they score significantly higher on the vertical axis (precision) compared to biomass methods, they face similar challenges on the horizontal axis: the synthesis of the template/MOF itself can involve high energy consumption and toxic reagents, the template removal process is not environmentally friendly, and the costs of scaling up are high with low yields. Therefore, their overall sustainability and scale-up potential are limited, placing them slightly above the lower-left quadrant (Olowoyo et al., 2024; Singh, 2025).

The fourth quadrant (low precision, high sustainability/expansion potential) is clearly anchored in biomass-derived carbon strategies (Belluati et al., 2024; He et al., 2023). It has a prominent advantage on the horizontal axis: the raw materials come from waste, are low-cost, and their large-scale acquisition and processing are practically feasible (Othman and Saad, 2024). However, the heterogeneity of natural precursors leads to the poorest structural controllability of their products, located at the bottom of the longitudinal axis (Kim et al., 2025). The challenge lies in climbing along the vertical axis through craftsmanship and pre-treatment (Lei et al., 2021).

It should be noted that MOF-derived carbon is a special case, with its position near the boundary between the second and third quadrants (Chai et al., 2025; Xu et al., 2023). It inherits some ‘designability’ from bottom-up synthesis, achieving high structural precision (especially in porosity and doping uniformity), but like templating methods, it scores low on the sustainability axis because of cost and scalability issues (Kim M. et al., 2024; Shahzadi et al., 2024). This distribution chart clearly reveals a reality: no single strategy is ‘all-powerful’. Every technology makes its own trade-offs between ‘precision and controllability’ and ‘green scalability’.

To make the above positioning quantitative rather than rhetorical, Table 1 summarizes representative metrics for the four strategies, and Figure 21 plots them on the sustainability–precision–scalability map.

**FIGURE 21:**
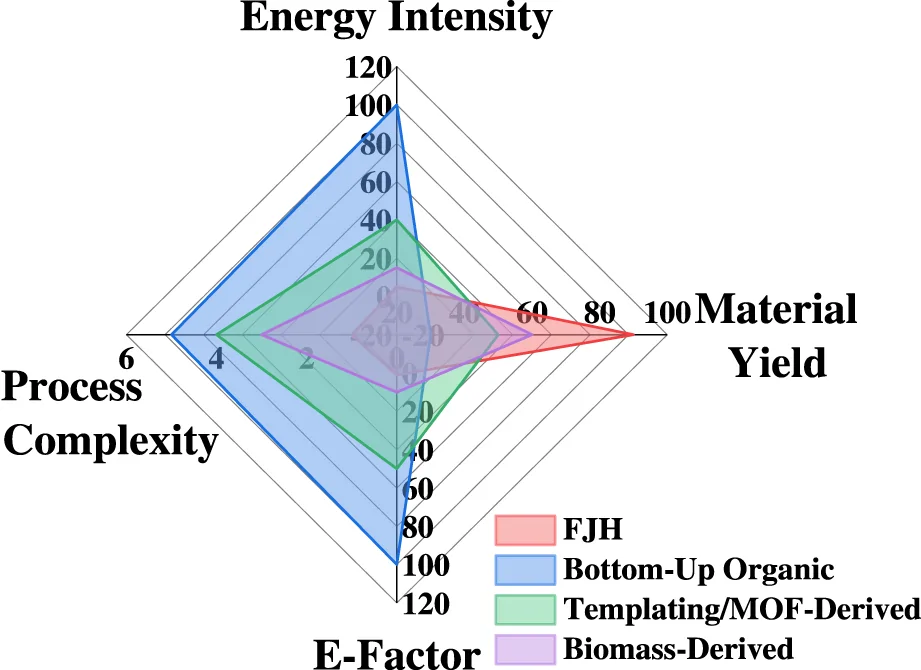
Quantitative four-quadrant strategy-comparison chart positioning the four green-synthesis strategies within the sustainability–precision–scalability framework. Each indicator reaches its maximum value in Table 1.

Table 5 complements this quantitative map with a qualitative, family-by-family audit of the seven representative carbon sources/strategies examined in Sections 2–5, contrasting for each its key advantages, main disadvantages, outstanding challenges, and most promising future direction. Read together with Table 1 and Figure 21, it makes the trade-off structure explicit: the strength of any one family maps almost one-to-one onto the weakness of another—low cost and scalability (biomass-derived) against 13–21 wt% yields and reagent/toxicity costs (MOF/template-derived), or angstrom-level precision (bottom-up) against 10–30% yields and milligram-to-gram scale—which is why the cross-fusion pathways in Section 6.3 matter more than the search for a single “best” route.

**TABLE 5:** Advantages, disadvantages, challenges, and future perspective of carbon materials from the seven representative sources/strategies discussed in this review.

Carbon family (source)	Key advantages	Main disadvantages	Outstanding challenges	Future perspective
Biomass-derived	Renewable/waste feedstock; low cost; most scalable; circular-economy value	High heterogeneity; ash (SiO2) content; broad pore distribution; batch scatter	Reproducible pre-treatment; online process control; yield optimization	ML-guided structure-property prediction; tiered utilization of feedstocks (Ali et al., 2021b; Chen et al., 2024b; Hall et al., 2025; Hamidu et al., 2025; Jiang et al., 2023; Joshi and Manjare, 2024; Razzak, 2024; Wang et al., 2023a; Yruela-Garrido et al., 2025; Zhang et al., 2025c; Zhang et al., 2025b; Zhong et al., 2025)
MOF/template-derived	Atomic-level precision; uniform heteroatom doping; ordered inheritable porosity	Low yield (13–21 wt%); expensive linkers/metal salts; energy-intensive pyrolysis	Cost of ligands/metals; metal-residue removal; gram-scale limit	Low-cost ligands; continuous MOF synthesis; metal recycling (Biswal et al., 2024; Ding et al., 2022)
Hard-template	Ultra-high fidelity ordered mesopores; narrow PSD	HF etching (toxic); low yield; costly template synthesis	Template recovery; green etchants	Silica recycling; non-HF removal (Ghaedi and Zhao, 2022; Nath et al., 2025)
Soft-template	Simple; low cost; flexible pore-size tuning	Less ordered than hard template; surfactant removal	Order control at scale	Block-copolymer design; scalable co-assembly (Chauhan, 2023; Chen et al., 2024c; Di Spirito et al., 2024; Hernik et al., 2025; Huang et al., 2023a; Kontogiannis et al., 2023; Robertson et al., 2022; Suresh et al., 2025)
Flash Joule heating (FJH)	Seconds-scale; low/net-negative energy; waste upcycling	Needs conductive network; arcing risk	Uniformity; generality to insulators	Continuous reactors; hybrid heating (Algozeeb et al., 2020; Cheng et al., 2022; Wyss and Silva, 2023)
Microwave-assisted	Selective bulk heating; rapid	High energy in some cases; penetration limits	Scale-up uniformity	Hybrid/hollow heating; process coupling (Kmür et al., 2025; Luong et al., 2020; Zou et al., 2025)
Bottom-up (organic)	Angstrom-level edge/structure precision	Low yield (10%–30%); >5 unit ops; high cost	Scalability; automation	Flow chemistry; automated synthesis (Crista et al., 2020b)

Future development will either involve pushing a particular strategy toward the ideal first quadrant (such as improving the accuracy of FJH or reducing the cost of bottom-up synthesis), or taking a more creative path—integrating strategies from different quadrants to complement each other’s strengths.

### 6.2 Application-oriented strategy selection guide: inferring synthetic routes from performance requirements

In engineering practice, the choice of the optimal synthesis route should be based on the core performance requirements of the end application.

For supercapacitor or battery electrode materials, the core requirement is a high specific surface area and appropriate pore-size distribution to ensure rapid ion transport, while maintaining high conductivity and low cost (Guo et al., 2021). In this context, the preferred strategy should lean toward biomass pyrolysis combined with chemical activation to maximize specific surface area and cost advantages; if the project focuses more on production efficiency and conductivity, new physical technologies (such as FJH or microwave methods) can be considered (Hosny et al., 2025; Obiora et al., 2026). Although template methods (such as ordered mesoporous carbon) can provide very high-performance models, their cost sensitivity makes them better suited for high-performance model research than for large-scale industrial applications.

In electrocatalysis (including ORR, HER, and OER), the core demand has shifted toward high-density active sites with specific electronic structures (such as M-Nx-C or certain heteroatom doping), while maintaining high conductivity and good stability (Miao et al., 2022). At this point, the preferred strategy should focus on MOF-derived carbon materials to take advantage of their ideal platform for achieving atomically dispersed metal sites and uniform doping (Chai et al., 2025; Zhang H. et al., 2025). If resources are limited, one can use carefully designed biomass-derived doped carbon (especially using nitrogen-rich biomass), while model molecules synthesized from the bottom up are mainly used for mechanistic studies (Khandaker et al., 2025).

For adsorption and separation applications, the core requirements are specific pore sizes for target molecules, abundant surface functional groups, and high adsorption capacity, while also considering regenerability and low cost (Akhtar et al., 2024; Hao et al., 2021). Under such demand, biomass-derived activated carbon is the preferred first choice for bulk adsorption applications; if the application scenario requires unconventional or uniform pore-precision separation, template carbon is generally the more reliable option, although carefully engineered biomass-derived carbons can approach similar uniformity for certain separations (Kim et al., 2025; Wang Q. et al., 2024).

In high-end electronics, optoelectronic devices, and quantum technology, the core demands are highly concentrated on atomically perfect lattice structures, well-defined energy bands and spin states, and extremely low defect densities (Lee et al., 2026; Saraswat et al., 2021). Given the stringent requirements for structural consistency in such applications, bottom-up organic synthesis is currently the most suitable option because alternative methods still fall short of the atomic-level defect control required; however, advances in template-guided CVD are progressively narrowing this gap for selected electronic applications (Lee et al., 2026; Saraswat et al., 2021).

### 6.3 An innovative path of cross-fusion: creating the next-generation of ‘hybrid’ advantages

Future breakthroughs are most likely to arise from the conscious integration of strategies. This is not a simple accumulation, but a ‘chemical grafting’ based on a deep understanding of each mechanism, aimed at creating new technological pathways that combine the advantages of the parent technologies. This marks a brand-new paradigm shift in materials science: from the linear evolution of a single technology to the systemic integration and co-evolution across disciplines and scales.

First, Pathway A (green, precise) represents the deep upgrading of biomass utilization. The traditional biomass carbonization process is often likened to ‘taking everything as it is,’ meaning that the chaotic natural structures are directly converted into carbonized products, making it difficult to control their microscopic structure precisely (Wu et al., 2024). In the future green pathway, scientists have proposed a new concept based on ‘molecular engineering’ and ‘structured empowerment’ (Zimmerman et al., 2020). Scheme A1 advocates depolymerizing macromolecules such as lignin and cellulose into relatively uniform platform compounds (such as vanillin and furfural), which have highly predictable and designable chemical structures (Vaz, 2024). Subsequently, these depolymerized products are converted into polymer precursors with preset mesostructures through controlled polymerization or co-assembly techniques, such as the soft template method (Kumar L. et al., 2021). This strategy is equivalent to introducing the precise control capabilities of polymer science upstream of biomass carbonization, enabling the final carbonized product not only to retain the green attributes of biomass but also to achieve a precise pore-size distribution and structural order (Wang R. et al., 2024). Plan A2 explores using nano-biochar (partially carbonized and partially homogenized) as a ‘carbon-based building material,’ filling it into removable green templates (such as thermally decomposable biomass-based templates, ice templates, or recyclable salt templates) (Rajput and Minkina, 2024). This “secondary shaping” strategy leverages the excellent mechanical strength and thermal stability of biochar, combined with the shape-control advantages of the template method, enabling large-scale preparation of carbon blocks with macroscopic engineered pores/mesoporous structures and providing a new structural basis for energy storage and sensing applications.

Secondly, Path B (precise and rapid) aims to address the bottleneck in the large-scale preparation of high-precision structures. Traditional bottom-up molecular synthesis is flawless in microscopic precision, but it is often limited in macroscopic yield and has low production efficiency (Palagati and Reddy, 2024). Plan B1 proposes a cutting-edge idea of combining ‘designed molecules’ with ‘flash processing.’ Scientists achieve this by precisely synthesizing linear or network polymers with specific aromatic structures that are easy to graphitize (Choi et al., 2021). Subsequently, using Flash Joule Heating (FJH) technology, ‘flash graphitization’ of these designed polymer precursors is carried out under millisecond-scale ultrafast physical processes (Wang C. et al., 2024). These non-equilibrium extreme conditions can ‘freeze’ the ideal polymer topology, effectively preventing the random coiling of polymer segments and the introduction of defects that occur during conventional thermal processing (Zhu et al., 2024). As a result, it is possible to mass-produce graphene or graphene-like materials with defect structures close to the theoretical design, perfectly inheriting the high-precision characteristics of molecular design (Liu Y. et al., 2025). Plan B2 utilizes microwave-assisted chemical vapor deposition (CVD) technology, introducing precisely synthesized nano-graphene molecules as ‘structural seeds’ or ‘nucleating agents’ into the growth substrate (Asif and Saha, 2023; Dutta et al., 2022). The rapid, uniform heating provided by microwaves, combined with high-quality initial nucleation from the seeds, can guide the epitaxial growth of large-area, uniformly oriented graphene or carbon nanotube films, maximizing the preservation of microscopic structural order and integrity while maintaining macroscopic dimensions (Arun et al., 2025).

Furthermore, Path C (Engineered Green) focuses on addressing the environmental footprint issues of the templating method. While the templating method offers significant advantages in morphology control, traditional inorganic templates such as silicone and quartz are expensive, non-degradable, or require high-temperature and high-pressure processing (Valencia et al., 2022). Plan C1 advocates developing bio-based templates, such as using liquid crystal phases or gel networks formed by the self-assembly of natural polymers like cellulose nanocrystals, chitosan, and bacterial cellulose as soft templates (Lacerda et al., 2023a). Alternatively, carved wood, rice husk silica, and other biogenic minerals can be used as hard templates. These natural templates can be removed after carbonization through mild chemical or physical methods, or partially retained to form composite materials, greatly reducing environmental impact (He et al., 2023). Plan C2 proposes recyclable/convertible templates, such as low-melting-point alloys or ionic liquids. These materials can be easily recovered and reused through physical methods (such as melting or dissolving) after carbonization, or the templates themselves can be carbon precursors (such as specific polymers), directly converting into part of the carbon framework after carbonization, achieving an ‘integrated template and precursor’ approach, similar to a more cost-effective metal-organic framework (MOF) strategy.

To avoid overstating readiness, the cross-fusion pathways above should be graded by level of evidence. Experimentally demonstrated today: (i) depolymerization of lignin/cellulose into platform molecules followed by soft-templated reassembly has been realized for porous carbons (Kumar L. et al., 2021; Wang R. et al., 2024); (ii) bio-based and recyclable templates (cellulose nanocrystals, salt, ice) have shaped carbons without HF (He et al., 2023); (iii) flash graphitization of designed polymer precursors into graphene-like materials has been demonstrated (Algozeeb et al., 2020; Liu Y. et al., 2025; Wang C. et al., 2024). Still largely conceptual/future: (i) coupling microwave-CVD “seeds” for wafer-scale single-crystal graphene remains at the prototype stage (Arun et al., 2025; Asif and Saha, 2023); (ii) closed-loop “integrated template + precursor” convertible templates and full digital-twin control of hybrid lines are forward-looking proposals requiring pilot validation. The demonstrated routes can be recommended now for near-term adoption, whereas the conceptual ones define the research agenda rather than present practice.

Therefore, future material manufacturing will no longer be the isolated development of a single technology, but a system integration process full of dynamic interactions. Through cross-scale strategy integration and digital process control, we will achieve coordinated green biomass supply, precise molecular synthesis, structural design through template engineering, and efficient production using novel physical technologies. This not only reconciles the historical conflicts between ‘precision’ and ‘scale’ and between ‘performance’ and ‘sustainability,’ but also opens a new era of carbon material manufacturing characterized by on-demand design and intelligent production.

## 7 Conclusion

This paper systematically examines the profound changes underway in carbon material synthesis. The traditional “extensive manufacturing” paradigm, which relies on fossil precursors, consumes high energy, and lacks precise structural control, can no longer meet the dual needs of sustainable development and high-end technology applications. In this regard, four emerging strategies represented by biomass-derived carbon, template-assisted synthesis, new physical technologies (flash joule heating, microwave synthesis) and bottom-up organic synthesis have launched impacts from different dimensions such as precursor sources, morphological engineering, process intensification and atomic manufacturing, respectively, and jointly promote the evolution of the field to a new paradigm of “green intelligent manufacturing” with raw material recycling, process low-carbonization and structural precision. The biomass path shifts the synthetic foundation from geological mining to the biosphere cycle, reflecting the sustainability of the source. The template method and MOF-derived carbon realize the “according to the diagram” control of pores and composition, highlighting the structure’s designability. Technologies such as flash Joule heating have reshaped process economics with extreme energy and time efficiency. At the same time, bottom-up synthesis has reached the scientific peak of atomic-precision control, revealing the intrinsic logic of structure determining performance. However, each strategy faces unique challenges in the “impossible triangle” composed of “structural controllable precision”, “sustainability” and “scalability”: the biomass path is limited by precursor heterogeneity, the template method is trapped by cost and environmental footprint, new physical technologies urgently need to solve the problems of uniformity and process control, and bottom-up synthesis is deeply trapped in the scale gap. This leads to a core conclusion: no single strategy can conquer the field, and future breakthroughs will increasingly rely on deep understanding, rational comparison, and creative cross-integration of these strategies.

Looking ahead, to advance this paradigm shift and ultimately achieve the on-demand design and intelligent manufacturing of sustainable carbon materials, breakthroughs are urgently needed in the following key research directions:

Whether it is the complex reactions of biomass in subcritical water, the framework evolution of MOFs during pyrolysis, or the millisecond-scale non-equilibrium transformations during flash Joule heating, the underlying microscopic mechanisms are largely still a ‘black box.’ Vigorously developing *in situ*/operando characterization techniques suitable for extreme conditions (such as high-temperature *in situ* transmission electron microscopy, synchrotron X-ray absorption spectroscopy, and millisecond time-resolved spectroscopy) is essential to capture the true distribution of reaction intermediates, local structural evolution, and temperature fields in real time. At the same time, multi-scale computational simulations (ranging from molecular dynamics to first-principles calculations) are needed to reveal the energy pathways and kinetic competition of carbon ring formation, defect generation, and heteroatom migration at the atomic level. Only by clarifying the causal chain between ‘process parameters—dynamic pathways—final structure’ can we shift from trial-and-error experimentation to rational prediction, laying the scientific foundation for precise regulation.

Facing the ‘curse of dimensionality’ caused by the vast synthetic parameter space and the complex structure–performance relationships, data-driven approaches will be key to breaking through. Efforts should focus on building standardized materials databases that encompass precursor information, process parameters, characterization data, and performance indicators. On this basis, machine learning and artificial intelligence algorithms can uncover hidden structure–property relationships and construct high-precision performance prediction models. Furthermore, by integrating high-throughput experimental platforms with automated synthesis robots, an ‘active learning’ closed loop can be realized: models guide experimental design, and experimental data feedback optimizes the models, ultimately achieving ‘inverse design’ for specific target performances—i.e., given a target performance, the algorithm recommends the optimal precursors and synthesis schemes. This will significantly accelerate the discovery of new materials and new processes. Recent surveys of artificial intelligence and data science in green and sustainable chemistry confirm that this workflow is already delivering measurable benefits: machine-learning surrogate models combined with Bayesian optimisation and active learning routinely reduce the number of experiments needed to optimise yield, selectivity and energy demand by roughly an order of magnitude, while improving batch-to-batch controllability and enabling closed-loop, self-optimising synthesis.

Concrete, evidence-based take-home messages from this analysis are as follows. (1) Biomass routes offer the lowest feedstock cost and inherent circularity but only 20–35 wt% carbon retention and batch variability, making them ideal for bulk sorbents and conductive fillers rather than precision parts. (2) MOF/template routes deliver unmatched porosity yet at 13–21 wt% carbon productivity and high reagent/toxicity cost, limiting them to model and high-value catalysts. (3) FJH and microwave compress time/energy by 2–3 orders versus furnaces—FJH graphene needs only ≈7.2 kJ g^-1^ and emits ≈10–16 kg CO_2_ kg^-1^, >90% below conventional graphene—but cede atomic-level control. (4) Bottom-up synthesis alone reaches atomic precision, at <1% yield and milligram scale. Translating these trade-offs into practice requires the four cross-cutting enablers emphasized above—quantitative life-cycle assessment (LCA) as a mandatory “green” benchmark, AI/data-driven inverse design built on standardized materials databases, engineered scale-up of the most promising routes, and community process standardization—so that strategy selection becomes evidence-based rather than assertion-based.

Currently, claims that a synthetic method is “green” or “sustainable” often lack unified, quantifiable benchmarks, which can easily lead to subjective assertions. Therefore, establishing universal, quantifiable life cycle assessment standards is crucial. This requires systematically and transparently accounting for comprehensive indicators throughout the entire chain—from raw material acquisition, transportation, and synthesis to product use and even disposal and recycling—including energy consumption, carbon emissions, water resource use, toxic substance emissions, and economic costs. Only through such a standardized “ecological ledger” can we objectively compare the carbon sequestration benefits of biomass pathways with potential agricultural water consumption, or assess whether the high performance of MOF-derived carbon is sufficient to offset the high energy consumption in synthesizing its precursors, thereby guiding resources in the field toward genuinely environmentally friendly technologies that offer advantages across the full life cycle. This is reinforced by recent reviews of green synthesis methodology, which call for routine reporting of energy intensity, E-factor, process mass intensity, and input renewability alongside performance data, so green claims remain falsifiable and comparable between studies.

The ultimate vision of green and intelligent manufacturing of carbon materials requires breaking down disciplinary barriers. Future leading research teams will need to deeply integrate the knowledge and methods of synthetic chemistry (designing molecules and understanding reactions), materials engineering (controlling structure and performance), process engineering (scaling up and optimizing processes), and data science (analysis and prediction). Specifically, this is reflected in the fact that precursors designed by chemists need to match the continuous-flow reactors developed by process engineers; predictions from models trained by data scientists need to directly guide process adjustments by materials engineers. We call for the establishment of more substantial interdisciplinary collaboration platforms to cultivate versatile talents with ‘chemical intuition, engineering thinking, and data literacy.’ Through such deep convergence, we can master the aforementioned cross-disciplinary integration paths (such as green and precise, precise and rapid) and elevate the production of sustainable carbon materials from a fragmented field dependent on individual technological breakthroughs to an intelligent systems-engineering approach that integrates design, manufacturing, evaluation, and optimization.

Ultimately, this article depicts not merely a collection of new technologies, but a revolution in how we think about creating matter. The goal is to shift from being a passive ‘discoverer’ and ‘user’ of materials to becoming an active ‘designer’ and ‘maker’ who can thoughtfully consider both the Earth’s boundaries and human needs. The path toward a sustainable future is fraught with challenges. Still, through relentless exploration of scientific mechanisms, rational integration of technological pathways, and sincere embrace of interdisciplinary wisdom, we have reason to believe that carbon materials—this ancient carrier of elements—will, under the new paradigm of green intelligent manufacturing, unleash a renewed vitality that supports the sustainable development of future civilization.

## References

[B1] Abbas W. Chaudhry F. Farooq U. Baber M. Z. Sulaiman T. A. (2025). Exploring the metric dimensions of graphene: unveiling the intricacies of its atomic structure. Mod. Phys. Lett. B 39, 2550007. 10.1142/s0217984925500071

[B2] Abdi G. Tendulkar R. Thatte C. Mishra S. Desai B. Surve S. (2024). “Scaling up nature’s chemistry: a guide to industrial production of valuable metabolites,” in Advances in Metabolomics (Springer), 331–375.

[B3] Adeoye A. O. Lawal O. S. Quadri R. O. Malomo D. Aliyu M. T. Dang G. E. (2023). “Sustainable energy via thermochemical and biochemical conversion of biomass wastes for biofuel production,” in Transportation Energy and Dynamics (Springer), 245–306.

[B4] Advincula P. B. A. (2022). Synthesis and Applications of Flash Graphene and Flash Joule Heating. Rice University ProQuest Dissertations & Theses, 31533142.

[B5] Afridi A. M. Aktary M. Shaheen Shah S. Mitu Sheikh S. I. Jahirul Islam G. Nasiruzzaman Shaikh M. (2024). Advancing electrical engineering with biomass-derived carbon materials: applications, innovations, and future directions. Chem. Rec. 24, e202400144. 10.1002/tcr.202400144 39529417

[B6] Aggarwal P. Umar F. Paul A. (2025). Review of ordered pores in nanomaterials for energy applications ranging from energy storage to catalysis. ACS Appl. Nano Mater. 8, 17780–17818. 10.1021/acsanm.5c03047

[B7] Ahmad R. Khan U. A. Iqbal N. Noor T. (2020). Zeolitic imidazolate framework (ZIF)-Derived porous carbon materials for supercapacitors: an overview. RSC Advances 10, 43733–43750. 10.1039/d0ra08560j PMC9058430 35519688

[B8] Ahmed Y. W. Loukanov A. Tsai H. C. (2025). State-of-the-art synthesis of porous polymer materials and their several fantastic biomedical applications: a review. Adv. Healthc. Mater. 14, 2403743. 10.1002/adhm.202403743 39723689

[B9] Akande O. Okolie J. A. (2024). Metal-Organic Frameworks (MOFs) Water Splitting for Heavy Metal Extraction: Opportunities and Challenges. *SSRN 4898528* .

[B10] Akhtar M. S. Ali S. Zaman W. (2024). Innovative adsorbents for pollutant removal: exploring the latest research and applications. Molecules 29, 4317. 10.3390/molecules29184317 PMC11433758 39339312

[B11] Algozeeb W. A. Savas P. E. Luong D. X. Chen W. Kittrell C. Bhat M. (2020). Flash graphene from plastic waste. ACS Nano 14, 15595–15604. 10.1021/acsnano.0c06328 33119255

[B12] Ali S. H. Emran M. Y. Gomaa H. (2021a). “Rice husk-derived nanomaterials for potential applications,” in Waste Recycling Technologies for Nanomaterials Manufacturing (Springer), 541–588.

[B13] Ali S. H. Emran M. Y. Gomaa H. (2021b). Rice Husk-Derived Nanomaterials for Potential Applications.

[B14] Ali A. Chiang Y. W. Santos R. M. (2022). X-ray diffraction techniques for mineral characterization: a review for engineers of the fundamentals, applications, and research directions. Minerals 12, 205. 10.3390/min12020205

[B15] Alzeer J. (2024). Beyond disorder: a new perspective on entropy in chemistry. Am. J. Med. Chemos 5, 1–5. 10.31487/j.AJMC.2024.01.01

[B16] Antinucci G. Dereli B. Vittoria A. Budzelaar P. H. Cipullo R. Goryunov G. P. (2022). Selection of low-dimensional 3-D geometric descriptors for accurate enantioselectivity prediction. ACS Catalysis 12, 6934–6945. 10.1021/acscatal.2c00976

[B17] Arun A. P. Sreenivasan N. Patil J. H. Kusanur R. Ramachandraiah H. L. Ramakrishna M. (2025). Thin films for next generation technologies: a comprehensive review of fundamentals, growth, deposition strategies, applications, and emerging frontiers. Processes 13, 3846. 10.3390/pr13123846

[B18] Asasian-Kolur N. Sharifian S. Haddadi B. Jordan C. Harasek M. (2024). Ordered porous carbon preparation by hard templating approach for hydrogen adsorption application. Biomass Convers. Biorefinery 14, 18381–18416. 10.1007/s13399-023-04282-x

[B19] Asgari M. Albacete P. Menon D. Lyu Y. Chen X. Fairen-Jimenez D. (2025). The structuring of porous reticular materials for energy applications at industrial scales. Chem. Soc. Rev. 54, 4701–4744. 10.1039/d5cs00166h 40195939 PMC11976391

[B20] Asif F. C. Saha G. C. (2023). Graphene-like carbon structure synthesis from biomass pyrolysis: a critical review on feedstock–process–properties relationship. C 9, 31. 10.3390/c9010031

[B21] Ayanda O. S. Mmuoegbulam A. O. Okezie O. Durumin Iya N. I. Mohammed S. E. James P. H. (2024). Recent progress in carbon-based nanomaterials: critical review. J. Nanoparticle Res. 26, 106. 10.1007/s11051-024-06006-2

[B22] Bafqi M. S. S. Aliyeva N. Baskan-Bayrak H. Dogan S. Okan B. S. (2024). Turning CO2 into sustainable graphene: a comprehensive review of recent synthesis techniques and developments. Nano Futur. 8, 022002. 10.1088/2399-1984/ad4fd5

[B23] Barrett G. (2024). “Development of high throughput metabolomics to aid the synthetic biology,” in Design-Build-Test-Learn’Cycle.

[B24] Basha O. M. Gamwo I. K. Wang R. Morsi B. I. (2026). Non-catalytic direct Methane-to-Methanol synthesis in an intensified helical-coil reactor. Energy and Fuels 40, 18639–18666. 10.1021/acs.energyfuels.6c02261

[B25] Bello R. S. Olorunnisola A. O. Omoniyi T. E. Onilude M. A. (2024). Effects of feedstock pretreatment and binder selection on briquette characterization. Nat. Resour. Conservation Res. 7, 5292. 10.24294/nrcr.v7i1.5292

[B26] Belluati M. Tabasso S. Gaudino E. C. Cravotto G. Manzoli M. (2024). Biomass-derived carbon-based catalysts for lignocellulosic biomass and waste valorisation: a circular approach. Green Chem. 26, 8642–8668. 10.1039/d4gc00606b

[B27] Biswal B. K. Zhang B. Thi M. T. P. Zhang J. Balasubramanian R. (2024). Recycling of spent lithium-ion batteries for a sustainable future: recent advancements. Chem. Soc. Rev. 53, 5552–5592. 10.1039/d3cs00898c 38644694

[B28] Blachowicz T. Ehrmann G. Ehrmann A. (2023). Recent developments in additive manufacturing of conductive polymer composites. Macromol. Mater. Eng. 308, 2200692. 10.1002/mame.202200692

[B29] Boretti A. (2025). A narrative review of breakthroughs and future horizons in zero-dimensional nanomaterials. Opt. Quantum Electron. 57, 370. 10.1007/s11082-025-08288-9

[B30] Cai J. Wu Z. Wang S. Guo J. Fan M. Xu W. (2024). Exploring advanced microwave strategy for the synthesis of two-dimensional energy materials. Appl. Phys. Rev. 11, 041320. 10.1063/5.0231081

[B31] Cao K. Zhang S. Shi Y. Diao X. Wei R. Ji N. (2025). Catalytic upgrading of plastic wastes into high-value carbon nanomaterials: synthesis and applications. ACS Nano 19, 12734–12761. 10.1021/acsnano.5c03391 40156546

[B32] Carrasco Díaz A. Abdelouahed L. Brodu N. Montes-Jiménez V. Taouk B. (2024). Upgrading of pyrolysis bio-oil by catalytic hydrodeoxygenation, a review focused on catalysts, model molecules, deactivation, and reaction routes. Molecules 29, 4325. 10.3390/molecules29184325 39339320 PMC11433775

[B33] Chai L. Li R. Sun Y. Zhou K. Pan J. (2025). MOF‐derived carbon‐based materials for energy‐related applications. Adv. Mater. 37, 2413658. 10.1002/adma.202413658 39791306

[B34] Chaikittisilp W. Yamauchi Y. Ariga K. (2022). Material evolution with nanotechnology, nanoarchitectonics, and materials informatics: what will be the next paradigm shift in nanoporous materials? Adv. Mater. 34, 2107212. 10.1002/adma.202107212 34637159

[B35] Chauhan S. (2023). Synthesis of ordered mesoporous carbon by soft template method. Mater. Today Proc. 81, 842–847. 10.1016/j.matpr.2021.04.257

[B36] Chen J. Qian J. (2024). Insights on MOF-derived metal–carbon nanostructures for oxygen evolution. Dalton Trans. 53, 2903–2916. 10.1039/d3dt04263d 38269643

[B37] Chen Z. Guo Y. Luo L. Liu Z. Miao W. Xia Y. (2024a). A critical review of hydrochar based photocatalysts by hydrothermal carbonization: synthesis, mechanisms, and applications. Biochar 6, 74. 10.1007/s42773-024-00364-9

[B38] Chen J. Ma X. Liang M. Guo Z. Cai Y. Zhu C. (2024b). “Physical–chemical–biological pretreatment for biomass degradation and industrial applications: a review,” in Waste (MDPI), 451–473.

[B39] Chen C. Zhang S. Huang B. Peng Y. Zhang Y. Wang L. (2024c). Block copolymers-enabled organic–organic assembly for unique carbonaceous Micro-/Nano-Structures. Adv. Funct. Mater. 34, 2410248. 10.1002/adfm.202410248

[B40] Chen F. Mu X. Zhou J. Wang S. Liu Z. Zhou D. (2024d). Engineering the active sites of MOF-derived catalysts: from oxygen activation to activate Metalair batteries. Chin. J. Chem. 42, 2520–2535. 10.1002/cjoc.202400332

[B41] Chen L. Cao G. Li Y. Zu G. Duan R. Bai Y. (2024e). A review on engineering transition metal compound catalysts to accelerate the redox kinetics of sulfur cathodes for lithium–sulfur batteries. Nano-Micro Lett. 16, 97. 10.1007/s40820-023-01299-9 PMC10825111 38285078

[B42] Chen Z. Cao Y. He Y. (2025a). A comprehensive review of surface roughness effects on microwave performance of transmission lines. IEEE Trans. Microw. Theory Tech. 73 (10), 7141–7163.

[B43] Chen H. Wei J. Pan F. Yuan T. Fang Y. Wang Q. (2025b). Advances in photonic crystal research for structural color. Adv. Mater. Technol. 10, 2400865. 10.1002/admt.202400865

[B44] Cheng Y. Cui G. Liu C. Liu Z. Yan L. Liu B. (2022). Electric current aligning component units during graphene fiber joule heating. Adv. Funct. Mater. 32, 2103493. 10.1002/adfm.202103493

[B45] Choi G. H. Nam M. G. Kwak S. J. Kim S. H. Chang H. Shin C.-S. (2021). Modularly aromatic-knit graphitizable phenolic network as a tailored platform for electrochemical applications. Energy and Environ. Sci. 14, 3203–3215. 10.1039/d1ee00402f

[B46] Chong K. C. Tam T. L. D. Tao R. Chin K. L. O. Chua M. H. Xu J. (2025). Conjugated ladder polymers: Unique organic π-Conjugated materials for optoelectronics, transistors, and energy harvesting and storage. Energy Mater. Adv. 6, 0134. 10.34133/energymatadv.0134

[B47] Chu Y. Liu F. Jian B. Hou Y. Huo F. (2025). Molecular dynamics for nanoconfined ion and water transport: mechanisms and applications in functional nanofluidic devices. ACS Appl. Mater. and Interfaces 17, 68841–68868. 10.1021/acsami.5c15216 41385335

[B48] Cook A. B. Clemons T. D. (2022). Bottom-up *versus* top-down strategies for morphology control in polymer-based biomedical materials. Adv. NanoBiomed Res. 2, 2100087. 10.1002/anbr.202100087

[B49] Crista D. Esteves da Silva J. Pinto da Silva L. (2020a). Evaluation of different bottom-up routes for the fabrication of carbon dots. Nanomaterials 10 (7), 1316. 10.3390/nano10071316 32635483 PMC7407658

[B50] Crista D. M. A. Silva JCGED Silva L. P. D. (2020b). Evaluation of different Bottom-up routes for the fabrication of carbon dots. Nanomaterials 73 (10), 7141–7163. 10.1039/d1ee00402f PMC7407658 32635483

[B51] Cui J. Su P. Li W. Wang X. Zhang Y. Xiao Z. (2025). Advanced cellulose-derived hard carbon as anode for sodium-ion batteries: mechanisms, optimization, and challenges. Adv. Energy Mater. 15, 2404604. 10.1002/aenm.202404604

[B52] Dai L. Zeng Z. Yang Q. Yang S. Wang Y. Liu Y. (2020). Synthesis of iron nanoparticles-based hydrochar catalyst for *ex-situ* catalytic microwave-assisted pyrolysis of lignocellulosic biomass to renewable phenols. Fuel 279, 118532. 10.1016/j.fuel.2020.118532

[B53] Deng B. Eddy L. Wyss K. M. Tiwary C. S. Tour J. M. (2025). Flash joule heating for synthesis, upcycling and remediation. Nat. Rev. Clean. Technol. 1, 32–54. 10.1038/s44359-024-00002-4

[B54] Derakhshandeh S. H. Nousiainen J. Piekkari M. Karvo J. Auer A. Luukkanen S. (2026). Characterization of the sedimentation and drying processes of complex mining tailings materials using NMR. Phys. Chem. Chem. Phys. 28, 52–66. 10.1039/d5cp03347k 41347442

[B55] Di Spirito N. A. Grizzuti N. Pasquino R. (2024). Self-assembly of pluronics: a critical review and relevant applications. Phys. Fluids 36, 111302. 10.1063/5.0238690

[B56] Ding J. Tang Y. Zheng S. Zhang S. Xue H. Kong Q. (2022). The synthesis of MOF derived carbon and its application in water treatment. Nano Res. 15, 6793–6818. 10.1007/s12274-022-4327-1

[B57] Ding P. Chen D. Ko P. K. Qammar M. Geng P. Guo L. (2025). Nanomaterials for spin-based quantum information. Nanoscale. 10.1039/d4nr04012k 39620885

[B58] Divyashree A. Hegde G. (2015). Activated carbon nanospheres derived from bio-waste materials for supercapacitor applications – a review. RSC Adv. 5, 88339–88352. 10.1039/c5ra19392c

[B59] Durak H. (2023). Comprehensive assessment of thermochemical processes for sustainable waste management and resource recovery. Processes 11, 2092. 10.3390/pr11072092

[B60] Dutta V. Verma R. Gopalkrishnan C. Yuan M.-H. Batoo K. M. Jayavel R. (2022). Bio-inspired synthesis of carbon-based nanomaterials and their potential environmental applications: a state-of-the-art review. Inorganics 10, 169. 10.3390/inorganics10100169

[B61] Etafa Y. Elangovan S. Legesse K. Chewaka M. (2025). “Synthesis and fabrication techniques for nanomaterials,” in Exploring Nanomaterial Synthesis, Characterization, and Applications (IGI Global), 241–266.

[B62] Evered S. J. Bluvstein D. Kalinowski M. Ebadi S. Manovitz T. Zhou H. (2023). High-fidelity parallel entangling gates on a neutral-atom quantum computer. Nature 622, 268–272. 10.1038/s41586-023-06481-y 37821591 PMC10567572

[B63] Eze V. H. U. Tamball J. S. Uzoma O. F. Sarah I. Robert O. Okafor W. (2024). Advancements in energy efficiency technologies for thermal systems: a comprehensive review. INOSR Appl. Sci. 12, 1–20. 10.59298/inosras/2024/1.1.1010

[B64] Fahad Halim A. Poinern G. E. J. Fawcett D. Sharma R. Surendran S. R R. (2025). Biomass-derived carbon nanomaterials: synthesis and applications in textile wastewater treatment, sensors, energy storage, and conversion technologies. CleanMat 2, 4–58. 10.1002/clem.15

[B65] Farghadan R. (2024). Mechanical spin splitting in zigzag graphene nanoribbons. Phys. Rev. B 110, 195411. 10.1103/physrevb.110.195411

[B66] Fatima M. Ahmad F. Noor S. Shafique S. Ibrahim A. B. Habib M. A. (2025). Application of carbon-based electrocatalysts derived from waste materials in fuel cells and electrolyzers. Sustain. Energy and Fuels.

[B67] Fayaz M. U. Wang Q. Liang S. Bai H. Pan F. Song C. (2024). Electric field induced ion migration and property tuning in functional oxides. Accounts Mater. Res. 5, 648–660. 10.1021/accountsmr.4c00067

[B68] Fonseca J. Meng L. Imaz I. Maspoch D. (2023). Self-assembly of colloidal metal–organic framework (MOF) particles. Chem. Soc. Rev. 52, 2528–2543. 10.1039/d2cs00858k 36930224

[B69] Gao X. Li Q. Liu F. Zhang S. Ko F. Wang H. (2026). Single-step lignin-derived ultramicroporous carbon membranes eradicating spontaneous combustion for efficient Pt single-atom catalysis in ambient formaldehyde oxidation. Carbon 253, 121378. 10.1016/j.carbon.2026.121378

[B70] Ghaedi H. Zhao M. (2022). Review on template removal techniques for synthesis of mesoporous silica materials. Energy and Fuels 36, 2424–2446. 10.1021/acs.energyfuels.1c04435

[B71] Ghamarpoor R. Fallah A. Fard N. E. Salehfekr S. Hossieini S. Moradi V. (2025). Synthesis and design of a multifunctional nanocomposite of TiO2 nanoparticles decorated on graphitic carbon derived from ZIF-12: towards enhanced photocatalytic degradation and supercapacitor performance. Surfaces Interfaces 76, 107846. 10.1016/j.surfin.2025.107846

[B72] Goldie S. J. Coleman K. S. (2023). Graphitization by metal particles. ACS Omega 8, 3278–3285. 10.1021/acsomega.2c06848 36713730 PMC9878637

[B73] Gong W. Chen Z. Dong J. Liu Y. Cui Y. (2022). Chiral metal–organic frameworks. Chem. Rev. 122, 9078–9144. 10.1021/acs.chemrev.1c00740 35344663

[B74] Gong H. Chen S. Tok J. B.-H. Bao Z. (2023). An emerging class of carbon materials: synthesis and applications of carbon flowers. Matter 6, 2206–2234. 10.1016/j.matt.2023.04.027

[B75] González Z. Acevedo B. Predeanu G. Axinte S. M. Drăgoescu M.-F. Slăvescu V. (2021). Graphene materials from microwave-derived carbon precursors. Fuel Process. Technol. 217, 106803. 10.1016/j.fuproc.2021.106803

[B76] Gu Y. Qiu Z. Müllen K. (2022). Nanographenes and graphene nanoribbons as multitalents of present and future materials science. J. Am. Chem. Soc. 144, 11499–11524. 10.1021/jacs.2c02491 35671225 PMC9264366

[B77] Gul S. Nasim F. Nadeem M. A. (2025). Single-atom metal-based electrocatalysts (fe, Ni, and co) for CO2-to-CO conversion: a comprehensive review. ACS Appl. Energy Mater. 8, 11786–11812. 10.1021/acsaem.5c01419

[B78] Guo W. Yu C. Li S. Qiu J. (2021). Toward commercial-level mass-loading electrodes for supercapacitors: opportunities, challenges and perspectives. Energy and Environ. Sci. 14, 576–601. 10.1039/d0ee02649b

[B79] Guo J. Yao Y. Yan X. Meng X. Wang Q. Zhang Y. (2024). Emerging carbon-based catalysts for the oxygen reduction reaction: insights into mechanisms and applications. Inorganics 12, 303. 10.3390/inorganics12120303

[B80] Gupta R. K. Pham P. V. (2024). Art etching of graphene. Nanoscale Horizons 9, 1230–1249. 38958571 10.1039/d4nh00077c

[B81] Gutiérrez M. Zhang Y. Tan J.-C. (2022). Confinement of luminescent guests in metal–organic frameworks: understanding pathways from synthesis and multimodal characterization to potential applications of LG@ MOF systems. Chem. Rev. 122, 10438–10483. 10.1021/acs.chemrev.1c00980 35427119 PMC9185685

[B82] Hall J. N. Miller J. H. Assary R. S. Baddour F. G. Dagle R. Dagle V. (2025). State of the art in thermal catalytic upgrading of biomass and biomass-derived intermediates. Annu. Rev. Chem. Biomol. Eng. 16, 371–408. 10.1146/annurev-chembioeng-082323-122317 40489304

[B83] Hamidu I. Afotey B. Kwakye-Awuah B. Anang D. A. (2025). Synthesis of silica and silicon from rice husk feedstock: a review. Heliyon 11, e42491. 10.1016/j.heliyon.2025.e42491 PMC11872444 40028602

[B84] Han Y.-C. Liu M.-L. Sun L. Li X.-C. Yao Y. Zhang C. (2022). A general strategy for overcoming the trade-off between ultrasmall size and high loading of MOF-Derived metal nanoparticles by millisecond pyrolysis. Nano Energy 97, 107125. 10.1016/j.nanoen.2022.107125

[B85] Hao S. Jia Z. Wen J. Li S. Peng W. Huang R. (2021). Progress in adsorptive membranes for separation–A review. Sep. Purif. Technol. 255, 117772. 10.1016/j.seppur.2020.117772

[B86] Hassan Z. Matt Y. Begum S. Tsotsalas M. Bräse S. (2020). Assembly of molecular building blocks into integrated complex functional molecular systems: structuring matter made to order. Adv. Funct. Mater. 30, 1907625. 10.1002/adfm.201907625

[B87] Hayder G. Naim R. M. (2025). Biochar-based nanocomposites from waste biomass: a sustainable approach for wastewater treatment and renewable bioenergy. Front. Agric. Sci. and Eng. 12.

[B88] He H. Zhang R. Zhang P. Wang P. Chen N. Qian B. (2023). Functional carbon from nature: biomass‐derived carbon materials and the recent progress of their applications. Adv. Science 10, 2205557. 10.1002/advs.202205557 PMC10238227 36988448

[B89] He Y. Wang B. Zeng S. Yang L. Weiqiao L. Xiao H. (2025). Physical field-assisted freeze drying for fresh foods: advances, challenges, and prospects. Food Rev. Int. 42, 1–30. 10.1080/87559129.2025.2602900

[B90] He C.-Y. Xu X.-Y. Wu Y.-Y. Sun G.-T. Wang Q.-S. Zhang Y.-Z. (2026). Radiative cooling materials and strategies for suppressing ice melting and enabling passive cold-chain management. J. Mater. Chem. A.

[B91] Hernik A. Smets J. Wang Z. Semenova Y. Tan J. C. Ameloot R. (2025). Optical detection of volatile organic compounds: a review of methods and functionalized sensing materials. Adv. Opt. Mater. 13, e00369. 10.1002/adom.202500369

[B92] Highmoore J. F. Kariyawasam L. S. Trenor S. R. Yang Y. (2024). Design of depolymerizable polymers toward a circular economy. Green Chem. 26, 2384–2420. 10.1039/d3gc04215d

[B93] Hosny M. Elbay A. S. Abdelfatah A. M. El-Maghrabi N. Fawzy M. (2025). Recent trends in transforming different waste materials into graphene via flash joule heating. Environ. Res. 270, 121033. 10.1016/j.envres.2025.121033 39914718

[B94] Hsu C.-Y. Rheima A. M. Mohammed M. S. Kadhim M. M. Mohammed S. H. Abbas F. H. (2023). Application of carbon nanotubes and graphene-based nanoadsorbents in water treatment. BioNanoScience 13, 1418–1436. 10.1007/s12668-023-01175-1

[B95] Hu X. Zuo D. Cheng S. Chen S. Liu Y. Bao W. (2023). Ultrafast materials synthesis and manufacturing techniques for emerging energy and environmental applications. Chem. Soc. Rev. 52, 1103–1128. 10.1039/d2cs00322h 36651148

[B96] Huang J. Liu C. Jin Y. Chen J. (2023a). Hierarchical porous carbon synthesis by carbonized polymer dots-based sacrificial template for high-performance supercapacitors. Chem. Eng. J. 461, 141930. 10.1016/j.cej.2023.141930

[B97] Huang Y. Chen Y. Xu M. Ly A. Gili A. Murphy E. (2023b). Catalysts by pyrolysis: transforming metal-organic frameworks (MOFs) precursors into metal-nitrogen-carbon (MNC) materials. Mater. Today 69, 66–78. 10.1016/j.mattod.2023.08.007

[B98] Iliescu A. Oppenheim J. J. Sun C. Dincǎ M. (2023). Conceptual and practical aspects of metal–organic frameworks for solid–gas reactions. Chem. Rev. 123, 6197–6232. 10.1021/acs.chemrev.2c00537 36802581

[B99] Islam A. Ghosh P. (2025). Frontiers in bond construction: innovative approaches to C–C and carbon–heteroatom (C–S, C–N, C–O, C–Se, C–P) transformations-a review. RSC Advances 15, 23704–23759. 10.1039/d5ra03124a 40642459 PMC12242412

[B100] Iurchenkova A. (2025). Sustainable carbon materials from biomass: pyrolysis and laser-induced carbonisation for energy storage applications. Acta Univ. Ups.

[B101] Jansen J. J. Heavey C. Mom T. J. Simsek Z. Zahra S. A. (2023). Scaling-up: building, leading and sustaining rapid growth over time. J. Manag. Stud. 60, 581–604. 10.1111/joms.12910

[B102] Jiang B. Jiao H. Guo X. Chen G. Guo J. Wu W. (2023). Lignin-based materials for additive manufacturing: chemistry, processing, structures, properties, and applications. Adv. Sci. 10, 2206055. 10.1002/advs.202206055 PMC10037990 36658694

[B103] Jiao J. Nie W. Yu T. Yang F. Zhang Q. Aihemaiti F. (2021). Multi-step continuous-flow organic synthesis: opportunities and challenges. Chemistry–A Eur. J. 27, 4817–4838. 10.1002/chem.202004477 33034923

[B104] Jiao S. Li C. Zhang Y. Gao J. Li Z. Liu K. (2023). ZIF-8-templated synthesis of core-shell structured IPOP@ MOF hybrid-derived nitrogen-doped porous carbon for efficient oxygen reduction electrocatalysis and supercapacitor. Electrochimica Acta 441, 141817. 10.1016/j.electacta.2023.141817

[B105] Jimenez Forero J. A. (2024). Valorisation of Waste Biomass and Tyres Through Thermochemical Processing: Upgrading Strategies and Carbon Functionalisation for Sustainable Resource Recovery. Queensland University of Technology.

[B106] Jing W. Shen H. Qin R. Wu Q. Liu K. Zheng N. (2022). Surface and interface coordination chemistry learned from model heterogeneous metal nanocatalysts: from atomically dispersed catalysts to atomically precise clusters. Chem. Rev. 123, 5948–6002. 10.1021/acs.chemrev.2c00569 36574336

[B107] Joshi M. Manjare S. (2024). Chemical approaches for the biomass valorisation: a comprehensive review of pretreatment strategies. Environ. Sci. Pollut. Res. 31, 48928–48954. 10.1007/s11356-024-34473-6 39083176

[B108] Keçeci M. (2025). From Majorana Fermions to Quantum Devices: The Role of Nanomaterials in the Second Quantum Era. 10.5281/zenodo.15331067

[B109] Khandaker T. Islam T. Nandi A. Anik MAAM Hossain M. S. Hasan M. K. (2025). Biomass-derived carbon materials for sustainable energy applications: a comprehensive review. Sustain. Energy and Fuels 9, 693–723. 10.1039/d4se01393j

[B110] Kim H. (2017). ACID FERMENTATION OF FOOD WASTE LECAHTE FOR ON-SITE PRODUCTION OF DENITRIFICATION CARBON SOURCE.

[B111] Kim M. Xin R. Earnshaw J. Tang J. Hill J. P. Ashok A. (2022). MOF-Derived nanoporous carbons with diverse tunable nanoarchitectures. Nat. Protoc. 17, 2990–3027. 10.1038/s41596-022-00718-2 36064756

[B112] Kim H. S. Hyun J. C. Choi Y. Ha S. Kang D. H. Heo Y. H. (2024a). A new perspective for potassium intercalation chemistry in graphitic carbon materials. Energy Storage Mater. 70, 103514. 10.1016/j.ensm.2024.103514

[B113] Kim M. Leong K. K. Amiralian N. Bando Y. Ahamad T. Alshehri S. M. (2024b). Nanoarchitectured MOF-derived porous carbons: road to future carbon materials. Appl. Phys. Rev. 11, 041317. 10.1063/5.0213150

[B114] Kim S.-J. Maligal-Ganesh R. V. Mahmood J. Babar P. Yavuz C. T. (2025). Structural control over single-crystalline oxides for heterogeneous catalysis. Nat. Rev. Chem. 9, 1–18. 10.1038/s41570-025-00715-5 40295894

[B115] Kmür Ah Kzl A. Karaman C. (2025). Nature’s blueprint for energy: biomass-derived heteroatom-doped graphene materials for advanced energy applications. Carbon Lett. 35, 919–961.

[B116] Kömür A. İ. Kızıl Ç. Karaman C. (2025). Nature’s blueprint for energy: biomass-derived heteroatom-doped graphene materials for advanced energy applications. Carbon Lett. 35, 1–43. 10.1007/s42823-025-00892-9

[B117] Kontogiannis O. Selianitis D. Lagopati N. Pippa N. Pispas S. Gazouli M. (2023). Surfactant and block copolymer nanostructures: from design and development to nanomedicine preclinical studies. Pharmaceutics 15, 501. 10.3390/pharmaceutics15020501 PMC9963006 36839826

[B118] Kumar P. Choudhury D. (2025). “Green technologies: theories, systems, principles and applications,” in Evaluating Environmental Processes and Technologies (Springer), 149–195.

[B119] Kumar P. Kumar A. (2025). Review of morphology and size-biased microwave absorbing nanomaterials. ACS Appl. Nano Mater.

[B120] Kumar R. Sahoo S. Joanni E. Singh R. K. Kar K. K. (2021a). Microwave as a tool for synthesis of carbon-based electrodes for energy storage. ACS Appl. Mater. and Interfaces 14, 20306–20325. 10.1021/acsami.1c15934 34702030

[B121] Kumar L. Singh S. Horechyy A. Fery A. Nandan B. (2021b). Block copolymer template-directed catalytic systems: recent progress and perspectives. Membranes 11, 318. 10.3390/membranes11050318 PMC8146702 33925335

[B122] Kumar S. Ganaie M. M. Nagashima K. Biswas K. Tiwary C. S. Kumar M. (2026). High-entropy materials: design principles, microstructure engineering, synthesis, and applications. J. Mater. Sci., 1–41. 10.1007/s10853-026-13452-x

[B123] Lacerda T. M. Marcelino P. R. F. Silva J. A. C. Gandini A. (2023a). “Bio-based templates at the service of nanotechnology: a promising approach for a sustainable future,” in Biopolymers (Elsevier), 233–262.

[B124] Lacerda T. M. Marcelino P. R. F. Silva J. A. C. Gandini A. (2023b). Chapter 8 - Bio-Based Templates at the Service of Nanotechnology: A Promising Approach for a Sustainable Future. Biopolymers.

[B125] Lee J. H. Kim J. Y. Lee H.-J. Choi S.-J. Lee Y. J. Jang H. W. (2026). Beyond the silicon Plateau: a convergence of novel materials for transistor evolution. Nano-Micro Lett. 18, 69. 10.1007/s40820-025-01898-8 PMC12436270 40952566

[B126] Lei C. Xie Z. Wu K. Fu Q. (2021). Controlled vertically aligned structures in polymer composites: natural inspiration, structural processing, and functional application. Adv. Mater. 33, 2103495. 10.1002/adma.202103495 34590751

[B127] Li Z. Liu L. Wang Z. Gao P. Li G. K. (2023a). Synthesis and application of mesoporous materials: process status, technical problems, and development prospects: a mini-review. Energy and Fuels 37, 3413–3427. 10.1021/acs.energyfuels.2c03882

[B128] Li J. Yin D. Qin Y. (2023b). Carbon materials: structures, properties, synthesis and applications. Manuf. Rev. 10, 13. 10.1051/mfreview/2023011

[B129] Li X. Ding Y. Zhang H. He T. Hao J. Wu J. (2024). Pine sawdust derived ultra-high specific surface area activated carbon: towards high-performance hydrogen storage and supercapacitors. Int. J. Hydrogen Energy 84, 623–633. 10.1016/j.ijhydene.2024.08.225

[B130] Li W. Xu Y. Wang G. Xu T. Wang K. Zhai S. (2025a). Sustainable carbon-based catalyst materials derived from lignocellulosic biomass for energy storage and conversion: atomic modulation and properties improvement. Carbon Energy 7, e708. 10.1002/cey2.708

[B131] Li X. Guan X. Zhu L. Li H. Yin X. Sun S. (2025b). Electron transfer in catalysis: from fundamentals to strategies. Chem. Soc. Rev. 54, 11423–11467. 10.1039/d4cs00600c 41196661

[B132] Liu F. Fan Z. (2023). Defect engineering of two-dimensional materials for advanced energy conversion and storage. Chem. Soc. Reviews. 10.1039/d2cs00931e 36779475

[B133] Liu Z. Ling Q. Cai Y. Xu L. Su J. Yu K. (2022a). Synthesis of carbon-based nanomaterials and their application in pollution management. Nanoscale Adv. 4, 1246–1262. 10.1039/d1na00843a PMC9419251 36133685

[B134] Liu F. Li P. An H. Peng P. McLean B. Ding F. (2022b). Achievements and challenges of graphene chemical vapor deposition growth. Adv. Funct. Mater. 32, 2203191. 10.1002/adfm.202203191

[B135] Liu Z. Liu X. Wei P. Mo L. Chen J. Liu Z. (2025a). Novel biomass enhances supercapacitor performance by precisely controlling pore structure, nitrogen/oxygen (N/O) atom content, and functional group distribution. Industrial Crops Prod. 232, 121327. 10.1016/j.indcrop.2025.121327

[B136] Liu Y. Zhou J. Li C. Zhang H. Wang Y. Yan Y. (2025b). Interfacial coupling effects in two-dimensional ordered arrays for microwave attenuation. Nat. Commun. 16, 202. 10.1038/s41467-024-55776-9 PMC11696289 39747194

[B137] Liu W. Wu F. Fan M. Guo Y. Cai Y. Zhang S. (2026). Synergistic effects of lewis-brønsted dual acids on pore development of activated carbon in activation of cotton for enhanced adsorption of organic pollutants. Biomass Bioenergy 206, 108709. 10.1016/j.biombioe.2025.108709

[B138] López D. Zapata K. Ramírez-Valencia L. D. Bailón-García E. Carrasco-Marín F. Pérez-Cadenas A. F. (2025). Hydrochar from carbon quantum dots (CQDs) synthesis for photocatalytic and decontamination applications in presence of TiO2. Int. J. Mol. Sci. 26, 4958. 10.3390/ijms26104958 40430097 PMC12112227

[B139] Luo Z. Zhou J. (2025). “Thermal conversion of biomass,” in Handbook of Climate Change Mitigation and Adaptation (Springer), 1233–1289.

[B140] Luong D. X. Bets K. V. Algozeeb W. A. Stanford M. G. Kittrell C. Chen W. (2020). Gram-scale bottom-up flash graphene synthesis. Nature 577, 647–651. 10.1038/s41586-020-1938-0 31988511

[B141] Ma Z. Wang W. Xiong Y. Long Y. Shao Q. Wu L. (2025a). Carbon micro/nano machining toward miniaturized device: structural engineering, large-scale fabrication, and performance optimization. Small 21, 2400179. 10.1002/smll.202400179 39031523

[B142] Ma X. Zhang P. Feng X. Yang C. Li Y. Wu F. (2025b). Metal ion catalysis within hard carbon synthesis and electrode interface engineering for sodium-ion batteries. Adv. Funct. Mater. 35, 2504373. 10.1002/adfm.202504373

[B143] Mahmood A. Bai Z. Wang T. Lei Y. Wang S. Sun B. (2025). Enabling high-performance multivalent metal-ion batteries: current advances and future prospects. Chem. Soc. Rev. 10.1039/d4cs00929k 39887968

[B144] Majumdar A. Tran K. D. Malhotra D. Tran D. T. Kim N. H. Lee J. H. (2024). “Heteroatom-doped carbon allotropes in water-splitting application,” in Heteroatom-Doped Carbon Allotropes: Progress in Synthesis, Characterization, and Applications, 177–222.

[B145] Mandal M. Sahoo M. R. Mahanandia P. Prochowicz D. Mahapatra A. (2026). Biomassderived activated carbon for next-generation supercapacitors: from sustainable precursors to flexible device-level advances. Adv. Sustain. Syst. 10, e70566. 10.1002/adsu.70566

[B146] Maniscalco M. P. Volpe M. Messineo A. (2020). Hydrothermal carbonization as a valuable tool for energy and environmental applications: a review. Energies 13, 4098. 10.3390/en13164098

[B147] Martins M. Oliveira M. Borges A. D. (2026). Life cycle assessment of renewable hydrogen and bioenergy pathways transitioning from conventional to sustainable power generation. Sci. Rep. 10.1038/s41598-026-63845-w

[B148] Martins-Vieira J. C. Torres-Mayanga P. C. Lachos-Perez D. (2023). Hydrothermal processing of lignocellulosic biomass: an overview of subcritical and supercritical water hydrolysis. BioEnergy Res. 16, 1296–1317. 10.1007/s12155-022-10553-8

[B149] Mehdi R. Khoja A. H. Naqvi S. R. Gao N. Amin N. A. S. (2022). A review on production and surface modifications of biochar materials via biomass pyrolysis process for supercapacitor applications. Catalysts 12, 798. 10.3390/catal12070798

[B150] Meloni E. Iervolino G. Palma V. (2023). Basics of Microwave Heating and Recent Advances.

[B151] Mennani M. Ait Benhamou A. Mekkaoui A. A. El Bachraoui F. El Achaby M. Moubarik A. (2024). Probing the evolution in catalytic graphitization of biomass-based materials for enduring energetic applications. J. Mater. Chem. A 12, 6797–6825. 10.1039/d3ta07449h

[B152] Miao Z. Li S. Priest C. Wang T. Wu G. Li Q. (2022). Effective approaches for designing stable M–Nx/C oxygen-reduction catalysts for proton-exchange-membrane fuel cells. Adv. Materials 34, 2200595. 10.1002/adma.202200595 35338536

[B153] Mishra R. K. Yadav A. Mishra V. Mishra S. N. Singh D. S. Verma D. K. (2024). Fundamental theory of electromagnetic spectrum, dielectric and magnetic properties, molecular rotation, and the green chemistry of microwave heating equipment. Green Chem. Synthesis Microwaves Ultrasound, 21–67.

[B154] Modugno P. (2023). Influence of Reaction Conditions on Hydrothermal Carbonization of Monosaccharides. 10.1002/cssc.202101348 34542237

[B155] Mori R. (2023). Replacing all petroleum-based chemical products with natural biomass-based chemical products: a tutorial review. RSC Sustain. 1, 179–212. 10.1039/d2su00014h

[B156] Muñoz M. Flores D. Morillo G. Narváez R. Marcilla A. Rosero M. (2025). Comprehensive review of SBA-15 mesoporous silica: functionalization strategies, diffusion mechanisms, and emerging applications. Sustain. Chem. 6, 42. 10.3390/suschem6040042

[B157] Muzyka R. Misztal E. Hrabak J. Banks S. W. Sajdak M. (2023). Various biomass pyrolysis conditions influence the porosity and pore size distribution of biochar. Energy 263, 126128. 10.1016/j.energy.2022.126128

[B158] Na Z. Lin X. Li J. Liang Z. Chen S. Scriven M. (2026). Bottom-Up synthesis of pyrene-based polycyclic aromatic hydrocarbons with tunable emission properties. J. Org. Chem. 91, 10615–10624. 10.1021/acs.joc.6c00677 42503800

[B159] Nagappan S. Duraivel M. Hira S. A. Prabakar K. Ha C.-S. Joo S. H. (2022). Heteroatom-doped nanomaterials/core–shell nanostructure based electrocatalysts for the oxygen reduction reaction. J. Mater. Chem. A 10, 987–1021. 10.1039/d1ta09861f

[B160] Nanda A. S. Kadja G. T. (2024). Bio-based templates for generating hierarchical zeolites: an overview for greener synthesis pathway. J. Porous Mater. 31, 1155–1173. 10.1007/s10934-024-01586-9

[B161] Nandi R. Jha M. K. Guchhait S. K. Sutradhar D. Yadav S. (2023). Impact of KOH activation on rice husk derived porous activated carbon for carbon capture at flue gas alike temperatures with high CO2/N2 selectivity. ACS Omega 8, 4802–4812. 10.1021/acsomega.2c06955 36777600 PMC9910064

[B162] Narang P. Garcia C. A. Felser C. (2021). The topology of electronic band structures. Nat. Mater. 20, 293–300. 33139890 10.1038/s41563-020-00820-4

[B163] Narita A. Chen Z. Chen Q. Müllen K. (2019). Solution and on-surface synthesis of structurally defined graphene nanoribbons as a new family of semiconductors. Chem. Science 10, 964–975. 10.1039/c8sc03780a 30774890 PMC6349060

[B164] Natali M. Rallini M. Torre L. Puglia D. (2022). High temperature composites from renewable resources: a perspective on current technological challenges for the manufacturing of non-oil based high char yield matrices and carbon fibers. Front. Mater. 9, 805131. 10.3389/fmats.2022.805131

[B165] Nath S. Naha A. Saikia K. Choudhury C. P. Venkatramanan V. (2025). Degradation of organic pollutants using lignin-derived carbon materials as a sustainable approach to environmental remediation. Biotechnol. Sustain. Mater. 2, 11. 10.1186/s44316-025-00036-z

[B166] Ng Z. W. Yeoh K. Y. Hafyan R. H. Putranto A. Horri B. A. Zein S. H. (2025). Techno-economic analysis of production of octane booster components derived from lignin. Biomass Convers. Biorefinery 15, 3845–3859. 10.1007/s13399-023-05255-w

[B167] Nguyen M. K. Do H. H. Tran N. T. Lee Y.-C. (2025). Recent advances in porous graphitic frameworks: a critical mini-review. RSC Advances 15, 50019–50035. 10.1039/d5ra07821k 41409735 PMC12706579

[B168] Nisa Fu Tahir M. Khalid S. Amin N. Yin H. Long Y. (2025). Revolutionizing micro-scale energy storage by 0D carbon nanostructures: synthesis, integration, performance optimization mechanisms and sustainable applications. Adv. Funct. Mater. 35, 2418053. 10.1002/adfm.202418053

[B169] Niu W. Ma J. Feng X. (2022). Precise structural regulation and band-gap engineering of curved graphene nanoribbons. Accounts Chem. Res. 55, 3322–3333. 10.1021/acs.accounts.2c00550 36378659

[B170] Nobrega G. Cardoso B. Souza R. Pereira J. Pontes P. Catarino S. O. (2024). A review of novel heat transfer materials and fluids for aerospace applications. Aerospace 11, 275. 10.3390/aerospace11040275

[B171] Obiora N. K. Ujah C. O. Ekwueme B. N. Asadu C. O. Olubambi P. A. (2026). Production of sustainable graphene and its derivatives through flash joule heating: a systemic review. Mater. Sci. Energy Technol.

[B172] Ojewumi M. E. Chen G. (2024). Hydrochar production by hydrothermal carbonization: microwave versus supercritical water treatment. Biomass 4, 574–598. 10.3390/biomass4020031

[B173] Olowoyo J. O. Gharahshiran V. S. Zeng Y. Zhao Y. Zheng Y. (2024). Atomic/molecular layer deposition strategies for enhanced CO 2 capture, utilisation and storage materials. Chem. Soc. Rev. 53, 5428–5488. 10.1039/d3cs00759f 38682880

[B174] Othman A. A. E. Saad A. S. M. (2024). A 4-Dimensional strategy for managing construction waste originating during the design process within architectural design firms in Egypt. Int. J. Eng. Res. Afr. 70, 95–116. 10.4028/p-aa0qds

[B175] Palagati S. Reddy J. (2024). Synthesis by top-down and Bottom-Up. Adv. Mat. Prod. Char. Multidiscip. App 201.

[B176] Palagati S. Kudamala R. R. Satyanarayana S. V. Veeram J. R. Garg T. Parihar A. (2024). “Advanced materials synthesis by top-down and Bottom-Up approaches,” in Advanced Materials (CRC Press), 201–221.

[B177] Pan X. Wu S. Chen J. Zhou X. Chen X. Xin Z. (2024). Toward efficient biofuel production: a review of online upgrading methods for biomass pyrolysis. Energy and Fuels 38, 19414–19441. 10.1021/acs.energyfuels.4c04131

[B178] Panwar N. Pawar A. (2022). Influence of activation conditions on the physicochemical properties of activated biochar: a review. Biomass Convers. Biorefinery 12, 925–947. 10.1007/s13399-020-00870-3

[B179] Patrisia Y. Roy D. Gunasekara C. Fernando D. Law D. Setunge S. (2027). Life-cycle environmental and economic assessment of low-carbon roof purlin systems: implications for sustainable material selection. Environ. Impact Assess. Rev. 122, 108673. 10.1016/j.eiar.2026.108673

[B180] Peng J. Kang X. Zhao S. Zhao P. Ragauskas A. J. Si C. (2023). Growth mechanism of glucose-based hydrochar under the effects of acid and temperature regulation. J. Colloid Interface Science 630, 654–665. 10.1016/j.jcis.2022.10.044 36274402

[B181] Peng L. Peng H. Yuan Y. Song Y. Yang X. Sun P. (2026). Nanoemulsion modular assembly for the synthesis of functional mesoporous nanomaterials. Nat. Synth. 5, 162–179. 10.1038/s44160-025-00973-7

[B182] Pérez-Mayoral E. Godino-Ojer M. Matos I. Bernardo M. (2023). Opportunities from metal organic frameworks to develop porous carbons catalysts involved in fine chemical synthesis. Catalysts 13, 541. 10.3390/catal13030541

[B183] Petrović J. Ercegović M. Simić M. Koprivica M. Dimitrijević J. Jovanović A. (2024). Hydrothermal carbonization of waste biomass: a review of hydrochar preparation and environmental application. Processes 12, 207. 10.3390/pr12010207

[B184] Pyle D. Xiang Y. Li X. Wang R. Dong G. Yin J. (2026). Length-controlled synthesis of graphene nanoribbons. Chem. Mater. 38, 1596–1612. 10.1021/acs.chemmater.5c02756

[B185] Qin X. Yu C. Zhou W. Xu Z. Chang J. Wang X. (2025). Joule heating: a versatile and sustainable heating strategy with diverse applications in materials science and waste management. J. Mater. Chem. A 13, 12828–12854. 10.1039/d4ta09092f

[B186] Rajput V. D. Minkina T. M. (2024). Nanomaterials and nano-biochar in Reducing Soil Stress: An Integrated Approach for Sustainable Agriculture. CRC Press.

[B187] Ramoso J. P. Rasekh M. Balachandran W. (2025). Graphene-based biosensors: enabling the next generation of diagnostic technologies—A review. Biosensors 15, 586. 10.3390/bios15090586 41002326 PMC12467994

[B188] Rangraz Y. Heravi M. M. (2021). Recent advances in metal-free heteroatom-doped carbon heterogonous catalysts. RSC Advances 11, 23725–23778. 10.1039/d1ra03446d 35479780 PMC9036543

[B189] Rasaq W. A. Okpala C. O. R. Igwegbe C. A. Białowiec A. (2024). Navigating pyrolysis implementation—A tutorial review on consideration factors and thermochemical operating methods for biomass conversion. Materials 17, 725. 10.3390/ma17030725 38591602 PMC10856175

[B190] Razzak S. A. (2024). Municipal solid and plastic waste co-pyrolysis towards sustainable renewable fuel and carbon materials: a comprehensive review. Chemistry–An Asian J. 19, e202400307. 10.1002/asia.202400307 38880993

[B191] Ren Y. Fan J.-s. Fu Y.-z. (2023). Recent strategies for improving the performances of rechargeable lithium batteries with sulfur-and oxygen-based conversion cathodes. Energy Mater. 3. N/A-N/A. 10.20517/energymater.2022.78

[B192] Reveshti A. M. Mansoub F. H. Reveshti J. N. Bonab K. F. (2026). Evaluation of thermal performance of innovative insulation materials for energy-efficient buildings in cold climates. Int. J. Thermofluids 32, 101544. 10.1016/j.ijft.2025.101544

[B193] Robertson M. Zagho M. M. Nazarenko S. Qiang Z. (2022). Mesoporous carbons from self-assembled polymers. J. Polym. Sci. 60, 2015–2042. 10.1002/pol.20220122

[B194] Roy J. J. Phuong D. M. Verma V. Chaudhary R. Carboni M. Meyer D. (2024). Direct recycling of li-ion batteries from cell to pack level: challenges and prospects on technology, scalability, sustainability, and economics. Carbon Energy 6, e492. 10.1002/cey2.492

[B195] Sabirov D. Ori O. Cataldo F. Putz M. V. (2024). Moving heptagons on fullerenes: topology, entangled stone–wales rotation groups, chemistry and beyond (+). Fullerenes, Nanotub. Carbon Nanostructures 32, 791–798. 10.1080/1536383x.2024.2312188

[B196] Saraswat V. Jacobberger R. M. Arnold M. S. (2021). Materials science challenges to graphene nanoribbon electronics. ACS Nano 15, 3674–3708. 10.1021/acsnano.0c07835 33656860

[B197] Sethuraman D. B. S. Liu C.-J. (2024). “Doping methods and their effects,” in Handbook of Semiconductors (CRC Press), 54–67.

[B198] Setiarto R. H. B. Afiati F. Setiyoningrum F. Priadi G. Octaviana S. (2026). Bioflavor production by fermentation and microbial biosynthesis mechanisms. Rev. Agric. Sci. 14, 37–63. 10.7831/ras.14.3_37

[B199] Sevilla M. Mokaya R. (2014). Energy storage applications of activated carbons: supercapacitors and hydrogen storage. Energy and Environ. Sci. 7, 1250–1280. 10.1039/c3ee43525c

[B200] Shahzadi S. Akhtar M. Arshad M. Ijaz M. H. Janjua MRSA (2024). A review on synthesis of MOF-derived carbon composites: innovations in electrochemical, environmental and electrocatalytic technologies. RSC Advances 14, 27575–27607. 10.1039/d4ra05183a 39228752 PMC11369977

[B201] Shakeel R. Ahmad A. Mushtaq H. Haider T. Javeria M. Asif A. (2025). Multifunctional nanomaterials in materials chemistry: a cross-disciplinary review of applications in energy, environment, catalysis, and biomedicine. Scholars J. Eng. Technol. 8, 673–707. 10.36347/sjet.2025.v13i08.007

[B202] Shi Z. Ci H. Yang X. Liu Z. Sun J. (2022). Direct-chemical vapor deposition-enabled graphene for emerging energy storage: versatility, essentiality, and possibility. ACS Nano 16, 11646–11675. 10.1021/acsnano.2c05745 35926221

[B203] Shi L. Wang B. Lu S. (2023). Efficient bottom-up synthesis of graphene quantum dots at an atomically precise level. Matter 6, 728–760. 10.1016/j.matt.2023.01.003

[B204] Shi H. Zhao J. Sun Z. Wang X. Hu X. Li X. (2025). Enhanced mechanical and electromagnetic shielding properties of Mg matrix layered composites reinforced with hybrid graphene nanosheet (GNS)–Carbon nanotube (CNT) networks. Materials 18, 3455. 10.3390/ma18153455 40805333 PMC12347964

[B205] Shi Y. Li H. Hua Q. Zhai J. Hu W. (2026). Electrically reconfigurable dual-mode synapses based on topological insulator nanowires for high-accuracy biometric recognition. Chem. Eng. J. 547, 180619. 10.1016/j.cej.2026.180619

[B206] Sielicki K. Matlak A. Chen X. Mijowska E. (2024). AlP compound and P-doping for promotion of electrocatalytic activity of N-doped carbon derived from metal-organic framework. J. Alloys Compd. 970, 172534. 10.1016/j.jallcom.2023.172534

[B207] Silva W. A. (2025). Microwave-assisted enantioselective synthesis of (2R,5S)-theaspirane: a green chemistry approach. Molecules 30. 10.3390/molecules30071519 PMC11990196 40286099

[B208] Singh B. (2025). Lanthanide-based catalysts for electrochemical water splitting: unraveling the interplay of structure, properties, and performance. Chem. Commun. 10.1039/d5cc02785c 40521993

[B209] Singh A. B. Yadav M. D. (2025). Advances and perspectives in ZIF-67 synthesis for hydrogen applications: a review. Energy and Fuels 39, 4610–4647. 10.1021/acs.energyfuels.4c05867

[B210] Singh A. Bin Abu Sofian A. D. A. Chan Y. J. Chakrabarty A. Selvarajoo A. Abakr Y. A. (2024). Hydrothermal carbonization: sustainable pathways for waste-to-energy conversion and biocoal production. GCB Bioenergy 16, e13150. 10.1111/gcbb.13150

[B211] Singh P. Panwar S. Dave P. N. (2025). Advancing environmental remediation with metal–organic frameworks: perspectives on green synthesis, scale-up strategies, techno-economic analysis, and life cycle assessment. Adv. Mater. Technol. 11, e01309. 10.1002/admt.202501309

[B212] Soltane H. B. Akkari F. C. Gallas B. Kanzari M. (2023). Structural, morphological, and optical properties of AgxO thin films deposited via obliquely angle deposition. Mater. Sci. Pol. 41, 27–41. 10.2478/msp-2023-0002

[B213] Song S. Su J. Telychko M. Li J. Li G. Li Y. (2021). On-surface synthesis of graphene nanostructures with π-magnetism. Chem. Soc. Rev. 10.1039/d0cs01060j 33481981

[B214] Song G. Shi Y. Jiang S. Pang H. (2023). Recent progress in MOF-derived porous materials as electrodes for high-performance lithium-ion batteries. Adv. Funct. Mater. 33, 2303121. 10.1002/adfm.202303121

[B215] Sriram G. Kurkuri M. Oh T. H. (2023). Recent trends in highly porous structured carbon electrodes for supercapacitor applications: a review. Energies 16, 4641. 10.3390/en16124641

[B216] Sun Z. Fang S. Hu Y. H. (2020). 3D graphene materials: from understanding to design and synthesis control. Chem. Rev. 120, 10336–10453. 10.1021/acs.chemrev.0c00083 32852197

[B217] Suresh A. Campos M. Xie K. Makris E. Lovejoy J. Shamsy M. E. (2025). Designing thermally compatible template-coating pairs toward dimensionally stable 3D porous carbons with tunable density. Adv. Funct. Mater. 36, e15814. 10.1002/adfm.202515814

[B218] Talkachov A. Babaev E. (2023). Wave functions and edge states in rectangular honeycomb lattices revisited: nanoflakes, armchair and zigzag nanoribbons, and nanotubes. Phys. Rev. B 107, 045419. 10.1103/physrevb.107.045419

[B219] Talwar P. Agudelo M. A. Nanda S. (2025). Pyrolysis process, reactors, products, and applications: a review. Energies 18, 2979. 10.3390/en18112979

[B220] Tan L. Jiao N. Bai X. Wang H. Wang J. Wang H. (2023). Methods for preparing hierarchical zeolites by chemical etching and their catalytic applications: a review. Eur. J. Inorg. Chem. 26, e202300314. 10.1002/ejic.202300314

[B221] Tan Z. Mahmood F. Tian M. Li Y. Zhang Q. Ma Z. (2026). Review of flash joule heating for the synthesis of graphene and other functional carbon materials. Carbon Energy 8, e70119. 10.1002/cey2.70119

[B222] Tang X. Xue H. Li J. Wang S. Yu J. Zeng T. (2024). Degradation of bisphenol A by nitrogen-rich ZIF-8-derived carbon materials-activated peroxymonosulfate. Toxics 12, 359. 10.3390/toxics12050359 38787138 PMC11125605

[B223] Tao H. Yan M. Zhang L. Zou Z. Han B. Dong H. (2024). Progress in preparation, processing, and application of high dielectric polypropylene matrix composite materials. Polym. Compos. 45, 4819–4838. 10.1002/pc.28111

[B224] Thakur A. Kumar A. Somya A. (2024). “Forestry and agricultural residues-based wastes: fundamentals, classification, properties, and applications,” in Biomass Wastes for Sustainable Industrial Applications (CRC Press), 95–139.

[B225] Tian X. Wang Y. Zeng Z. Dai L. Xu J. Cobb K. (2022). Research progress on the role of common metal catalysts in biomass pyrolysis: a state-of-the-art review. Green Chem. 24, 3922–3942. 10.1039/d1gc04537g

[B226] Tian X.-J. Guo S.-J. Wu Z.-Y. Xu N. Han G. Chai Y.-M. (2025). Advances and challenges of metal organic frameworks (MOFs) and derivatives in photoelectrocatalytic water splitting. Mater. Horizons. 10.1039/d5mh01457c 40990526

[B227] Tripathy S. Rodrigues J. Shimpi N. G. (2023). Top-down and Bottom-up approaches for synthesis of nanoparticles. Nanobiomaterials Perspect. Med. Appl. Diagn. Treat. Dis. 145, 92–130.

[B228] Valencia L. Handa R. Monti S. Jasso-Salcedo A. B. Georgouvelas D. Magaña I. (2022). On the mineralization of nanocellulose to produce functional hybrid materials. J. Mater. Chem. A 10, 9248–9276. 10.1039/d2ta00457g

[B229] van Blitterswijk R. H. (2024). “Adaptive model predictive control for microstructure control,” in Laser Material Processing (University of Waterloo).

[B230] Vaz Jr S. (2024). The Lignin Macromolecule: A Compendium of Sustainable Technologies. Springer.

[B231] Verma R. Nguyen A.-G. (2024). “Preparation and synthesis of rice husk-based activated carbon,” in Recent Advances in Activated Carbon (CRC Press), 157–169.

[B232] Veselov G. B. Vedyagin A. A. (2023). Resorcinol–formaldehyde-derived carbon xerogels: preparation, functionalization, and application aspects. Materials 16, 6566. 10.3390/ma16196566 37834703 PMC10573919

[B233] Vogt C. Weckhuysen B. M. (2022). The concept of active site in heterogeneous catalysis. Nat. Rev. Chem. 6, 89–111. 10.1038/s41570-021-00340-y 37117296

[B234] Wagih M. Balocchi L. Benassi F. Carvalho N. B. Chiao J.-C. Correia R. (2022). Microwave-enabled wearables: underpinning technologies, integration platforms, and next-generation roadmap. IEEE J. Microwaves 3, 193–226. 10.1109/jmw.2022.3223254

[B235] Wang X. Liu J. L. (2022). “Bottom-up synthesis of nanomaterials,” in Nanochemistry: From Theory to Application for In-Depth Understanding of Nanomaterials, 239.

[B236] Wang Z. Wei H. Huang Y. Wei Y. Chen J. (2023a). Naturally sourced hydrogels: emerging fundamental materials for next-generation healthcare sensing. Chem. Soc. Rev. 52, 2992–3034. 10.1039/d2cs00813k 37017633

[B237] Wang Q. Sun H. Wu S. Pan S. Cui D. Wu D. (2023b). Production of biomass-based carbon materials in hydrothermal media: a review of process parameters, activation treatments and practical applications. J. Energy Inst. 110, 101357. 10.1016/j.joei.2023.101357

[B238] Wang Q. Che J. Wu W. Hu Z. Liu X. Ren T. (2023c). Contributing factors of dielectric properties for polymer matrix composites. Polymers 15, 590. 10.3390/polym15030590 PMC9921423 36771891

[B239] Wang Q. Luo B. Wang Z. Hu Y. Du M. (2024a). Pore engineering in biomass-derived carbon materials for enhanced energy, catalysis, and environmental applications. Molecules 29, 5172. 10.3390/molecules29215172 PMC11547597 39519813

[B240] Wang R. Feng Y. Li D. Li K. Yan Y. (2024b). Towards the sustainable production of biomass-derived materials with smart functionality: a tutorial review. Green Chem. 26, 9075–9103. 10.1039/d4gc01771d

[B241] Wang C. Wang B. Su X. He R. (2024c). Synthesis of graphene from waste rubber powder based on flash joule heating method and its influence on the performance of cement mortar. Constr. Build. Mater. 451, 138871. 10.1016/j.conbuildmat.2024.138871

[B242] Wang J. Sun Z. Wu S. You X. Yang C. Xiong M. (2025a). Synergistic catalytic graphitization and pore orientation design in biomass-derived porous carbons for electrochemical applications. Biomass Bioenergy 200, 108021. 10.1016/j.biombioe.2025.108021

[B243] Wang N. Zhang X. Tan S. Lee S. Hu E. (2025b). X-ray diffraction studies of single-crystal materials for broad battery applications. Chem. Rev. 125, 9834–9874. 10.1021/acs.chemrev.5c00394 41041686

[B244] Wang W. Ding H. Bian J. Li W. Sun S. Chen D. (2026). Recent advances in non-noble metal-based metal-organic frameworks as electrocatalysts for water splitting: challenges and future prospects. J. Mater. Chem. A.

[B245] Wen X. Chi M. Bai L. Liu J. Sun B. (2025). Controllable synthesis of hierarchical pores of carboxyl-functionalized coconut shell activated carbon and its formaldehyde adsorption properties. J. Environ. Chem. Eng. 14, 120878. 10.1016/j.jece.2025.120878

[B246] Williams N. E. Oba O. A. Aydinlik N. P. (2022). Modification, production, and methods of KOH-activated carbon. ChemBioEng Rev. 9, 164–189. 10.1002/cben.202100030

[B247] Wu B. Meng H. Morales D. M. Zeng F. Zhu J. Wang B. (2022). Nitrogen-rich carbonaceous materials for advanced oxygen electrocatalysis: synthesis, characterization, and activity of nitrogen sites. Adv. Funct. Mater. 32, 2204137. 10.1002/adfm.202204137

[B248] Wu F. Xie A. Jiang L. Mukherjee S. Gao H. Shi J. (2023). Inorganic–organic hybrid dielectrics for energy conversion: mechanism, strategy, and applications. Adv. Funct. Mater. 33, 2212861. 10.1002/adfm.202212861

[B249] Wu Z. Sun J. Hu P. Wang W. (2024). “Preparation of biomass carbon materials and their several typical applications,” in Biomass Based Products (IntechOpen).

[B250] Wu P. Ma S. Zhu W. (2026). Green pathways to low-sulfur diesel: advances and challenges in desulfurization technologies. Green Chem.

[B251] Wyss K. M. Silva K. J. (2023). Tour KVBAAKHTHCCLBIYM: Synshesis of clean hydrogen gas from waste plastic at zero net Cost. Adv. Mater. 35, 2306763. 10.1002/adma.202306763 37694496

[B252] Wyss K. M. Luong D. X. Tour J. M. (2022). Large‐scale syntheses of 2D materials: flash joule heating and other methods. Adv. Materials 34, 2106970. 10.1002/adma.202106970 34695282

[B253] Wyss K. M. Silva K. J. Bets K. V. Algozeeb W. A. Kittrell C. Teng C. H. (2023). Synthesis of clean hydrogen gas from waste plastic at zero net cost. Adv. Mater. 35, 2306763. 10.1002/adma.202306763 37694496

[B254] Xia D. Li H. Huang P. Zhou W. Kulak A. Luo N. (2024). Flash Joule-heating synthesis of MoO2 nanocatalysts in graphene aerogel for deep catalytic oxidative desulfurization. AIChE J. 70, e18357. 10.1002/aic.18357

[B255] Xiao W. Cheng M. Liu Y. Wang J. Zhang G. Wei Z. (2023). Functional metal/carbon composites derived from metal–organic frameworks: insight into structures, properties, performances, and mechanisms. ACS Catal. 13, 1759–1790. 10.1021/acscatal.2c04807

[B256] Xiao J. Chen Y. Cheng L. Wu H. Hou M. Ma L. (2025). Flash joule heating: a transformative non-equilibrium strategy for next‐generation advanced materials. Small Methods 9, e01678. 10.1002/smtd.202501678 41263218

[B257] Xie X.-C. Huang K.-J. Wu X. (2018). Metal–organic framework derived hollow materials for electrochemical energy storage. J. Mater. Chem. A 6, 6754–6771. 10.1039/c8ta00612a

[B258] Xing H. Arif M. He G. Xiao H. Chen Y. Chen H. (2023). Insights into the critical roles of γ N atoms in understanding the oxygen reduction on MOF-Derived single-atom catalysts. Sci. China Mater. 66, 3200–3212. 10.1007/s40843-023-2447-4

[B259] Xing H. Liu Y. Deng X. Chi X. Zong Y. Feng J. (2026). *In situ* observation Zn volatilization and microstructural resolution in ZIF-8-derived porous carbon for supercapacitors. Nano Res. 19, 94908497. 10.26599/nr.2026.94908497

[B260] Xu Z.-X. Ma X.-Q. Shan Y.-Q. Hu X. Osman S. M. Liao J.-J. (2022). Artificial coal: facile and green production method via low-temperature hydrothermal carbonization of lignocellulose. ACS Sustain. Chem. and Eng. 10, 3335–3345. 10.1021/acssuschemeng.1c08338

[B261] Xu S. Dong A. Hu Y. Yang Z. Huang S. Qian J. (2023). Multidimensional MOF-derived carbon nanomaterials for multifunctional applications. J. Mater. Chem. A 11, 9721–9747. 10.1039/d3ta00239j

[B262] Xue G. Qin B. Ma C. Yin P. Liu C. Liu K. (2024). Large-area epitaxial growth of transition metal dichalcogenides. Chem. Rev. 124, 9785–9865. 10.1021/acs.chemrev.3c00851 39132950

[B263] Yadav S. Chandra H. Tiwari K. S. Bharty M. K. Chandravanshi R. Chauhan D. S. (2025). A review on the role of artificial intelligence and data science in green and sustainable chemistry: current trends and futuristic pathways. Discov. Chem. Eng. 6, 1. 10.1007/s43938-025-00104-w

[B264] Yadav S. Singh G. Mishra S. Prajapati H. C. Chauhan D. S. Quraishi M. A. (2026). Green synthesis methods: advances, challenges, and future perspectives. Sustain. Chem. One World 10, 100228. 10.1016/j.scowo.2026.100228

[B265] Yang W. Zhao Q. Zhou Y. Cui Z. Liu Y. (2022). Research progress of metal organic Frameworks/Carbon‐Based composites for microwave absorption. Adv. Eng. Mater. 24, 2100964. 10.1002/adem.202100964

[B266] Yin Y. Liu Q. Zhao Y. Chen T. Wang J. Gui L. (2023). Recent progress and future directions of biomass-derived hierarchical porous carbon: designing, preparation, and supercapacitor applications. Energy and Fuels 37, 3523–3554. 10.1021/acs.energyfuels.2c04093

[B267] Yoshikawa N. (2020). “Mechanism of microwave heating of matter,” in RF Power Semiconductor Generator Application in Heating and Energy Utilization (Springer), 71–89.

[B268] Yruela-Garrido M. Campos-Castellanos E. Morales M. V. Rodríguez-Ramos I. (2025). Guerrero-Ruiz A: Boron nitride-supported metal catalysts for the synthesis and decomposition of ammonia and formic acid. Nanomaterials 15, 212. 10.3390/nano15030212 39940188 PMC11819914

[B269] Yu H. Cao Y. (2024). “The synthesis methods of typical carbon materials,” in Carbon Catalysis (CRC Press), 502–532.

[B270] Yuan R. Jiao H. Zhu H. Fang D. Jiao S. (2023). *In situ* characterization techniques and methodologies for high-temperature electrochemistry. Chem 9, 2481–2508. 10.1016/j.chempr.2023.06.018

[B271] Yuan X. Yan C. Zhang S. Yang Y. Zhou S. Huang P. (2026). Flash joule-heating technology for material manufacturing, processing, and emerging applications. AIChE J. 72, e70215. 10.1002/aic.70215

[B272] Zeng D. Xiong H. Wu L. Zhang Y. Qi K. Guo X. (2022). High-energy graphite microcrystalline carbon for high-performance lithium-ion capacitor: diffusion kinetics and lithium-storage mechanism. J. Colloid Interface Sci. 623, 1190–1199. 10.1016/j.jcis.2022.05.111

[B273] Zhang Y. (2025). Play the City: Reconstructing Game Urban Design and Narrative Architecture Based on Spatial Poetics Theory. Politecnico di Torino.

[B274] Zhang D. Lu J. Pei C. Ni S. (2022a). Electrochemical activation, sintering, and reconstruction in energy-storage technologies: origin, development, and prospects. Adv. Energy Mater. 12, 2103689. 10.1002/aenm.202103689

[B275] Zhang H. Jin X. Lee J.-M. Wang X. (2022b). Tailoring of active sites from single to dual atom sites for highly efficient electrocatalysis. ACS Nano 16, 17572–17592. 10.1021/acsnano.2c06827 PMC9706812 36331385

[B276] Zhang K. Huang Z. Yang M. Liu M. Zhou Y. Zhan J. (2023). Recent progress in melt pyrolysis: fabrication and applications of high‐value carbon materials from abundant sources. SusMat 3, 558–580. 10.1002/sus2.165

[B277] Zhang X. Lv X. Qian Z. Chen C. Mao S. Lu J. (2024a). Template evolution induced relay self-assembly for mesoporous carbonaceous materials via hydrothermal carbonization. ACS Nano 18, 17826–17836. 10.1021/acsnano.4c03744 38935973

[B278] Zhang W. Kang K. Duan J. Li P. Zhang R. Wang J. (2024b). High content pyridine nitrogen-doped carbon nanosheets derived from ZIF-L as anode materials of lithium-ion batteries with excellent capacity and rate performance. J. Alloys Compd. 992, 174591. 10.1016/j.jallcom.2024.174591

[B279] Zhang H. Shen Z. Zeng R. Liang Q. Liu H. (2024c). Insights into pyrolysis product characteristics and carbon structure evolution of bituminous coal under high-temperature thermal shock. Fuel 371, 132096. 10.1016/j.fuel.2024.132096

[B280] Zhang Z. Zhang N. Zhang Z. (2025a). High-performance carbon nanotube electronic devices: progress and challenges. Micromachines 16, 554. 10.3390/mi16050554 PMC12113874 40428680

[B281] Zhang F. Yang X. Guan J. Qiu J. (2025b). Biomass-based single-atom catalysts: synthesis, structure regulation, and applications in energy conversion. Nano Res. 18, 94907865. 10.26599/nr.2025.94907865

[B282] Zhang J. Xia M. Wang J. Wu C. Li S. Liu L. (2025c). High-content graphitic-N self-doped porous carbon catalyst derived from seaweed for efficient oxygen reduction reaction. Ionics 31, 4643–4660. 10.1007/s11581-025-06196-2

[B283] Zhang G. Li H. Wang Q. Su Y. Sun Y. Pang H. (2025d). Metal-organic framework derivatives with complex architectures: controllable synthesis and applications. Nano Res. 18, 94907229. 10.26599/nr.2025.94907229

[B284] Zhang H. Wang S. Lv E. Qi M. He C. Dong X. (2025e). Porous organic material-based atomically dispersed metal electrocatalysts. Energy and Environ. Sci. 18, 3986–4009. 10.1039/d5ee00273g

[B285] Zhang C. Zheng K. Yang D. Ye X. Zhou L. Yun J. (2025f). Construction of metal-free heteroatoms doped carbon catalysts with certain structure and properties: strategies and methods. Carbon Lett. 35, 1–23. 10.1007/s42823-025-00903-9

[B286] Zhang J. Ghawri B. Dutta D. Fasel R. Calame M. Borin Barin G. (2026a). Bottom–up-synthesized graphene nanoribbons for nanoelectronics. Nat. Rev. Mater. 11, 1–19. 10.1038/s41578-025-00880-5

[B287] Zhang P. Shen Z. Jia L. Qiu J. (2026b). Biomass-derived carbon for boosting photocatalysis. Small 22, e12210. 10.1002/smll.202512210 41450254

[B288] Zhao H.-Y. Yu M.-Y. Liu J. Li X. Min P. Yu Z.-Z. (2022). Efficient preconstruction of three-dimensional graphene networks for thermally conductive polymer composites. Nano-micro Letters 14, 129. 10.1007/s40820-022-00878-6 35699797 PMC9198159

[B289] Zhao Z. Wu T. Li X. Chen Y. Meng X. (2025). Progress and perspectives of rapid joule heating for the preparation of highly efficient catalysts. Mater. Horizons 12, 734–759. 10.1039/d4mh01180e 39569835

[B290] Zheng-De L. Sheng-Cheng S. Ao L. Ming-Liang W. Ming-Yang Y. Yu H. (2019). Preparation and mechanical property of graphene-reinforced copper matrix composites. J. Inorg. Mater. 34, 469–477.

[B291] Zhong L. Zhu J. Liu S. Yang J. Lu X. Li J. (2025). Biochar-driven green separation and catalytic upgrading of bio-oil: Mechanisms, applications, and future perspectives. Energy and Fuels 39, 19977–19997. 10.1021/acs.energyfuels.5c02568

[B292] Zhou Y. Haq M. Z. U. (2025). Engineering of synthetic microbial consortia for sustainable management of wastewater and polyethylene terephthalate: a comprehensive review. Int. J. Mol. Sci. 26, 11623. 10.3390/ijms262311623 41373776 PMC12692444

[B293] Zhou C. Wang Y. (2020). Recent progress in the conversion of biomass wastes into functional materials for value-added applications. Sci. Technol. Adv. Mater. 21, 787–804. 10.1080/14686996.2020.1848213 33354165 PMC7738282

[B294] Zhou R. Xu B. Zheng T. Wang H. Yang M. Huang S. (2025). Sustainable resource utilization of industrial by-product gypsum: low-Energy rapid joule heating for calcium sulfide production. Chem. Eng. J. 520, 166210. 10.1016/j.cej.2025.166210

[B295] Zhu S.-B. Wang X.-F. (2025). Robust half-metallicity in edge-passivated zigzag tin carbide nanoribbons. Appl. Surf. Sci. 715, 164607. 10.1016/j.apsusc.2025.164607

[B296] Zhu G. Gao L. Sun Y. Wei W. Yan L.-T. (2024). Non-equilibrium structural and dynamic behaviors of active polymers in complex and crowded environments. Rep. Prog. Phys. 87, 054601. 10.1088/1361-6633/ad3e11 38608453

[B297] Zhuo C. Levendis Y. A. (2014). Upcycling waste plastics into carbon nanomaterials: a review. J. Appl. Polym. Sci. 131, app.39931. 10.1002/app.39931

[B298] Zimmerman J. B. Anastas P. T. Erythropel H. C. Leitner W. (2020). Designing for a green chemistry future. Science 367, 397–400. 10.1126/science.aay3060 31974246

[B299] Zito J. (2023). Colloidal Nanocrystals by Design.

[B300] Zou J. Tang L. Kang L. (2025). Innovative heating for the nano age: exploring the potentials of carbothermal shock. ACS Nano 19, 152–186. 10.1021/acsnano.4c12350 39745711

[B301] Zourou A. Ntziouni A. Karagianni A. Alizadeh N. Argirusis N. Antoniadou M. (2025). Recent advances in carbon dots-based photocatalysts for water treatment applications. Inorganics 13, 286. 10.3390/inorganics13090286

[B302] Zulhaimi H. I. Gopinath S. C. Kasim F. H. (2025). Active site in single atom catalyst: an update on dynamic behavior and rational design strategy. Crit. Rev. Solid State Mater. Sci., 1–29. 10.1080/10408436.2025.2592941

